# Standardization of *Berberis aristata* DC and *Nigella sativa* L. Using HPTLC and GCMS and Their Antineoplasia Activity in 7,12-Dimethylbenz[a]anthracene-Induced Mouse Models

**DOI:** 10.3389/fphar.2021.642067

**Published:** 2021-11-30

**Authors:** Mohd Mazhar, S S Agrawal

**Affiliations:** Department of Pharmacology, School of Pharmaceutical Sciences, Delhi Pharmaceutical Sciences & Research University, New Delhi, India

**Keywords:** antineoplasia, GCMS, HPTLC, molecular docking, phytopharmaceuticals

## Abstract

*Berberis aristata* DC and *Nigella sativa* L. are officially listed in various Indian Pharmacopoeia and AYUSH official documents. Prescribed for different ailments for proven medicinal activities, they thus became part of polyherbal medications. With reverse pharmacology and scientific validation, more than 30 patents are filed on different formulations of *B. aristata* and granted. *Nigella sativa* L. has been broadly studied for its therapeutic potential and wide range of activities against cardiovascular, diabetic, cancer, and life style disorders. Thus, this study is aimed at standardizing *B. aristata* and *N. sativa* and their antineoplasia activity in 7, 12-dimethylbenz[a]anthracene (DMBA)-induced mouse models. Molecular docking was done using the Schrodinger program Maestro 9.0. Herbal extracts and essential oil (*B. aristata* and *N. sativa*) were standardized and quantified using high-performance thin-layer chromatography (HPTLC) (CAMAG) and gas chromatography–mass spectrometry (GCMS) (Agilent 2010GC System) with validated methods. DMBA was administered orally once a week (1mg/200 µL) to each animal except the normal control. Hematology, histopathology, and immunoassays were performed, and data were analyzed and depicted with GraphPad and SPSS. In molecular docking, thymoquinone showed the highest docking score (9.519, 9.211, and 9.042, respectively) in the active site pockets of IL6 (PDB ID: 4CNI and 5FCU), TNF (PDB ID: 2AZ5), and VEGF (PDB ID: 4KZN). Out of all four target sites, thymoquinone and berberine showed good binding affinity with IL6 (PDB ID: 4CNI) compared to α- and β-pinenes. HPTLC analysis of the hydroalcoholic extract showed the presence of berberine both qualitatively and quantitatively (5.4% berberine), and thymoquinone detected 0.17% in the *N. sativa* extract. GCMS for essential oil showed 26 compounds including ±pinene. Leukocytes and erythrocytes of *N. sativa* and *B. aristata* were analyzed, and significant improvements were recorded (P < 0.05) and graphically presented. Mean survival time was calculated by the Kaplan Meier method (119 days). Immunoassay analyses were conducted, namely, TNF-*α* and VEGF, and interpreted and marked.

## Introduction

Certain chemotherapeutic agents have been aborted for use in the treatment of malignancy because of their toxicity. Over these years, there has been an increase in medical research in a broad range of therapeutics, many of which use plant-derived lead compounds (Vincristine and Paclitaxel) and are under extensive research for cancer cure. When the world is moving toward organic products, certain plants are thought to have enormous potential of cure against incurable diseases like cancer and COVID 19, and thereby, these create a pool of natural chemicals that may provide a therapeutic effect against cancer and other malignancies. Herbal medicines are in practice and considered the second method to treat cancer in developing nations (Kennedy, 2005; Ezeome and Anarado, 2007). *B. aristata* DC (family: Berberidaceae) is the main constituent of rasaut, darvyadi (kvatha, leha, taila), rasanjana, and Dashanga lepa. Aromoline, oxyberberine, oxyacanthine, berbamine, berberine chloride, 1-O-methylpakistanine, and pseudopalmatine chloride are the main chemical constituents of *B. aristata* (Pasrija et al., 2010). Antidiarrheal activity, cardiotonic hepatoprotective activity, antidiabetic activity, and anticancer activity are reported. Being potential therapeutic agents, more than 30 patents are filed on the different formulations granted and marketed (4, 5). *Nigella sativa* L. (Ranunculaceae) has been researched for its pharmacological potential and shown to possess a wide spectrum of activities, namely, diuretic, cardioprotective, antihyperglycemic, antineoplastic, analgesic, antimicrobial, anti-inflammatory, spasmolytic, hepatoprotective, nephroprotective, and antioxidant properties (6). Most of the therapeutic properties of this plant are due to the presence of thymoquinone (TQ), which is a major active chemical component of the essential oil. α-Pinene and β-pinene are other biomarkers that possess biological activity (6). In silico methods have been designed, developed, and applied to pharmacology hypothesis development and testing using software, for example, FoldX. In silico methods are primarily used alongside the generation of *in vitro* data and their correlation, with both to create the model and to test it. Such models have seen frequent use in the drug discovery and optimization of novel molecules with affinity to a target, the clarification of pharmacokinetics and pharmacodynamics, and physicochemical characterization. With this background, we aimed at standardizing *B. aristata* (bark) and *N. sativa* L. (black seed) using high-performance thin-layer chromatography (HPTLC) and gas chromatography–mass spectrometry (GCMS) and their antineoplasia activity in 7,12-dimethylbenz[a]anthracene (DMBA)-induced mouse models.

## Materials and Methodology

### Molecular Docking

Docking studies were performed to study the molecular binding energy and patterns of some natural derivatives with the active site pockets of crystal structures of IL6 (PDB ID: 4CNI and 5FCU), IL10 (PDB ID: 1WQ8), TNF (PDB ID: 2AZ5), and VEGF (PDB ID: 4KZN) using the Schrodinger program Maestro 9.0. The binding energies and their respective docking scores of all synthesized molecules are summarized in a tabular form and represented in Table 1.

**TABLE 1:** Docking and gliding with their respective PDB codes.

PDB	Compound	Docking score	Glide score
TNF-2AZ5	Thymoquinone	4.468	4.468
	Berberine	4.426	4.426
	α-Pinene	4.150	4.150
	β-Pinene	4.173	4.173
VEGF-4KZN	Thymoquinone	4.525	4.525
	Berberine	2.948	2.948
	α-Pinene	2.963	2.963
	β-Pinene	3.978	3.978

#### Collection and Authentication

*B. aristata* (bark) and *N. sativa* (seeds) were collected from Delhi, and both were authenticated by NISCAIR (Reference No. NISCAIR/RHMD/Consult/2018/3136-85-1/2).

#### Chemicals and Materials

Berberine chloride was procured from Prolab Marketing Pvt. Ltd. (India), and thymoquinone and α- and β-pinene were procured from TCI Chemicals (India).

All the described chemicals and reagents used in the present study were HPLC-grade-certified.

#### Extraction

1) *B. aristata* Bark: The bark (2 kg) was air-dried, crushed to smaller pieces, re-dried, and coarsely powdered and was then exhaustively extracted with water:alcohol (95%, methanol) (1:1) in Soxhlet’s apparatus for 72 h. The extract was dried using an Allied lyophilizer with a co-solvent system (the temperature at −80°C and the pressure at 760 Pa), and a dark-brown mass (2.5%) was obtained. 2) *Nigella sativa* L.: Continuous hot solvent extraction was performed with Soxhlet’s apparatus for 6 h at 40°C using a coarse powdered crude drug to hydroalcoholic methanol ratio of 1:10 gmL^−1^ (Alhaj et al., 2008), and the percentage yield obtained was 5.16%. 3) Essential oil extraction: In a Clevenger apparatus, each plant material was separately soaked in distilled water (1 L) at 90°C for 6 h (Nair et al., 2018). Using hydrodistillation, the volatile components were collected by the addition of equal volumes of n-hexane, dried over anhydrous sodium sulfate, stored in an amber-colored bottle (−20°C) with a label. The extract yield was calculated as 0.50 to 0.60% with repetitions.

### Preparation of Standard Solutions

The berberine Cl working standard was accurately weighed and transferred to 10 mg/10 ml and dissolved in methanol to obtain a solution concentration of 1 mcg/µL (for HPTLC), and in the same way, three sets of controls for berberine were prepared from a separate stock so as to lie in the lowest, middle, and highest regions of the calibration curves. Similarly, 1 ppm thymoquinone standard solution was prepared for the calibration curve.

### DPPH Assay

The 2,2-diphenyl-1-picryl-hydrazyl-hydrate (DPPH) assay was performed as per Brand-Williams et al. (1995). The reagent is prepared by dissolving DPPH (11.82 mg) in 500 ml of ethanol to meet 6 × 10^−5^ mol/L solution. α-Tocopherol acetate is treated as a standard antioxidant (1000 μg/ml with ethanol). The stock solution is further diluted to make concentrations of 20, 40, 60, 80, and 100 µg/ml with methanol. Each herbal extract test solution is prepared by dissolving 1000 μg/ml with ethanol. Methanol was used as a blank solution and control. 0.2 ml of methanol is mixed with 7.8 ml of DPPH, as shown in Table 2.

**TABLE 2:** Absorbance versus concentrations of α-tocopherol, *B. aristata*, and *N. sativa*.

Conc. (µg/ml)	% Inhibition of tocopherol	% Inhibition of *B. aristata*	% Inhibition of *N. sativa*
100	38	29	34
80	32	21	30
60	28	17	25
40	21	13	21.9
20	18.8	4	17.7
Mean	22.6	11.33333	25.72
SD	±7.13	±8.84	±6.44
Pearson correlation	0.991*	0.988*	0.988*
Sig. (two-tailed)	0 0.001	0.002	0.000
N	5	5	5

*Correlation is significant at the 0.01 level (two-tailed).

#### *In vitro* Activity (MTT Assay)

1 mg/10 ml of anastrazole used as a positive control and different concentrations of anastrazole (1, 10,100, 200, and 500 μM) were used. DMSO at 0.04% was used as a solvent control, whereas a negative control (medium) and positive control (anastrazole) were used as treatments. The cell line was purchased from the National Center for Cell Sciences, Pune, with a seeding density of 2.0 × 104 cells/well stored in liquid nitrogen for further testing purposes. Before the 3-(4,5-dimethylthiazol-2-yl)-2,5-diphenyltetrazolium bromide (MTT) assay, MCF-7 cells were thawed at 37 ± 1°C in a gaseous environment of 5 ± 1% carbon dioxide in a humid environment in tissue culture flasks containing the medium, Dulbecco’s modified Eagle’s medium (DMEM) (Genetix Biotech Asia) supplemented with 10% fetal bovine serum (Genetix Biotech Asia) and penicillin (100 units) and streptomycin (100 μg) antibiotics (Genetix Biotech Asia) to obtain the subconfluence of cells (70–90% confluent). Test solution concentrations are presented in Table 3.

**TABLE 3:** Final stock concentration in the study for MTT Assay.

S. No.	Test name	Final stock in DMEM	Concentration in RPMI
1	Thymoquinone	12.5 μg/ml	(2.5 ,5 ,7.5 ,10 ,12.5) µg/ml
2	Berberine chloride	1 µM	1 µM(10 ,100 ,200 ,500) nm
3	α-Pinene	100 μg/ml	(5, 10 ,20 ,30 ,40) µg/ml
4	β-Pinene	25 μg/ml	Aruna and Sivaramakrishnan (1990); Banchrof et al. (1996); Bower et al. (2002); Organization (2007); Saied et al. (2007)
5	*Berberis aristata* extract	500 μg/ml	(1, 10, 50, 300, 500) µg/ml
3	*Nigella sativa* extract	250 μg/ml	(250, 200, 170, 110, 45) µg/ml
4	*Nigella sativa* oil	1%	(0.60, 0.50, 0.40, 0.30, 0.20)%

### Instrumentation: (I) High-Performance Thin-Layer Chromatography

CAMAG Automatic TLC Sampler 4 (New ATS4) is used for the identification using a D_2_ lamp at a wavelength of 254 nm, with a PM high voltage (286 V). Methanol is used as the sample solvent type having a dosage speed of 150 nL/, an inert gas (N_2_) is used as the spray gas, and Merck silica gel 60F 254 is used as the stationary phase. The bond length is kept at 8.0 mm with a syringe size of 25 μL in all tracks. n-Butanol:acetic acid:water (3:1:1) (v/v/v/ ) and C_6_H_14_: CH3COOC2H5 (8:2) v/v are used as mobile phases in a twin trough chamber (20 × 10 cm) for *B. aristata* and *N. sativa* L., respectively, and drying is done at room temperature. Scanning is performed at a speed of 20 mm/s with slit dimensions of 6.00 × 0.45 mm.

2) GCMS analysis: Filtered through 0.25 μM PTFE; then, a volume of 2 μL was injected at a split ratio of 1:40 into an Agilent 2010 Gas Chromat System and a CTPAL autosampler with a silica column (30 m × 0.25 mm, film thickness 0.25 μm) and interfaced with an electron ion spray mass detector (m/z 40–650 at 1250 s/scan). The interface was set up at 290°C, and the ion source was adjusted to 220°C with the carrier gas (helium at 2 mLmin^−1^). After a 2 min solvent setback time at 70°C, the oven temperature was increased to 5°C/min–280°C with a solvent cut time of 4.50 min. The pressure was kept at 69.0 kPa, and a total flow of 52.6 mLmin^−1^, a column flow of 1.21 mLmin^−1^, and a linear velocity of 39.9 cm s^−1^ were maintained. The volatile constituents were identified by matching the retention index and fragmentation pattern data with those of the standards using WILEY and NIST(12).

### Animal Study

Female *Swiss albino* mice from the institute animal house, 14–15 weeks old, weighing 24–28 g, were arranged into 10 groups (*n*= 6 animals per group), and DMBA was administered orally once a week (1 mg/200 μL olive oil) to each animal except the normal control up to 5 weeks and classified as Group I: normal control, Group II: disease control, vehicle-treated Group III: marketed standard Anastrazole, 1 mg/kg, Group IV: thymoquinone, 10 mg/kg, Group V: α-pinene, 0.50 ml/kg, Group VI: β-pinene, 200 mg/kg, Group VII: *Nigella sativa* L. extract, 200 mg, Group VIII: *N. sativa* oil, 50 μl, Group IX: berberine Cl, 30 mg, Group X *B. aristata* extract, 400 mg). The standard and validated conditions maintained were a temperature of 20 ± 1 C and a relative humidity (RH) of 50 ± 5%, and mice were fed a standard rodent diet and water ad libitum and sustained at a 12 h light/dark cycle. All experimental procedures were carried out in compliance with the Declaration of Helsinki and the protocol approved by the Ethical Committee of DIPSAR (IAEC/2016-I-Prot.No-17), India. The blood samples were collected using a Retro-orbital, stored in a preheparinized vacutainer, stored under standard conditions, and sent to the lab, and doses are calculated as per OECD guidelines, 2014.

Tissue specimens from the mammary gland of female mice of each experimental groups were fixed in neutral buffered formalin 10% and processed by a conventional verified method, embedded in paraffin, sectioned at 4–5 μm, and stained with dyes, namely, hematoxylin and eosin (10).

Immnuhistochemistry: The procedure for immunohistochemical analysis was performed as described previously (Liu et al., 2009). Tumor tissues were sectioned at a thickness of 4 μm and incubated in methanol (0.3% H_2_O_2_) for 30 min and treated with 0.1% Triton X-100 for 10 min. The sections were washed with PBS three times and blocked with 1% bovine serum albumin and 0.01% Triton X-100 for 1 h at room temperature. The sections were incubated with the VEGF antibody (1:100) or TNF α (1:50) or the TGF β antibody overnight at 4°C. Negative controls were analyzed by staining with isotype-matched IgG or PBS. Following rinsing in PBS, the sections were incubated with anti-mouse biotinylated monoclonal secondary antibodies for 1 h at room temperature. The sections were washed three times with PBS and then incubated in avidin–biotin, and IHC slides were examined under a microscope (Olympus CX31; Olympus Corporation, Tokyo, Japan) and analyzed by Advance Histo lab.

## Results and Discussion

### Molecular Docking

**FIGURE 1:**
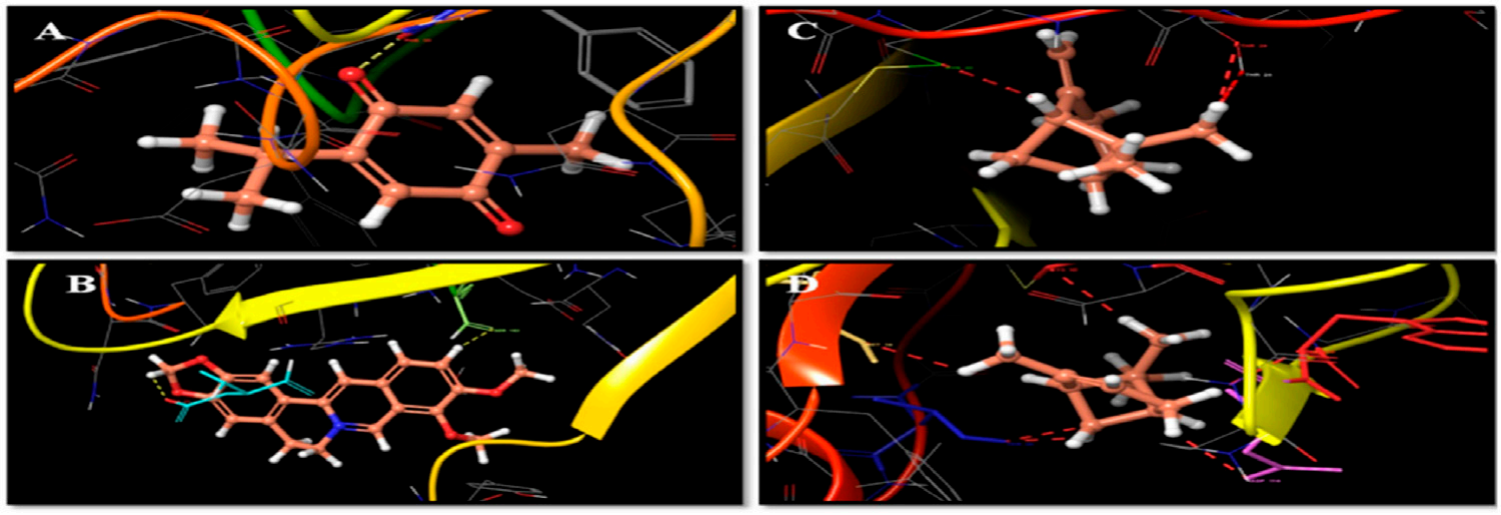
**(A)** Thymoquinone and amino acid PHE 33 interactions. **(B)** Berberine, amino acid ASP 31, and hydrogen bond interactions. **(C)** β-Pinene interaction with amino acid cysteine. **(D)** α-Pinene interaction with amino aspartate.

Docking studies were carried out to study the molecular binding pattern of natural derivatives with the active site pockets of crystal structures of IL6 (PDB ID: 4CNI and 5FCU), IL10 (PDB ID: 1WQ8), TNF (PDB ID: 2AZ5), and VEGF (PDB ID: 4KZN), and adaptable resolution (<2 A) and the docking score are the criteria. The binding energies and docking scores of all synthesized molecules are represented in Table 1. Docking of the compounds thymoquinone, berberine, α-pinene, and β-pinene into the active sites of enzymes revealed that several molecular interactions (hydrogen bonds, π-interactions, and hydrophobic interactions) were considered to be responsible for the observed affinity of the compound. Thymoquinone formed only one hydrogen bond between oxygen of the –C=O group of thymoquinone and amino acid PHE 33. Berberine formed one hydrogen bond between hydrogen of the dioxane ring of berberine and amino acid ASP 31, and another hydrogen bond is formed between hydrogen of the phenyl ring of berberine with SER 102 amino acid. α-Pinene and β-pinene showed no hydrogen bond; they only show bad contact with amino acids (Figures 1A,B,C,D) and showed the details of binding modes of the docked compounds thymoquinone, berberine, α-pinene, and β-pinene, thus exhibiting the antitumor properties in the targeted herbal drug extract.

### Anti-oxidant Activity (DPPH Assay)

**FIGURE 2:**
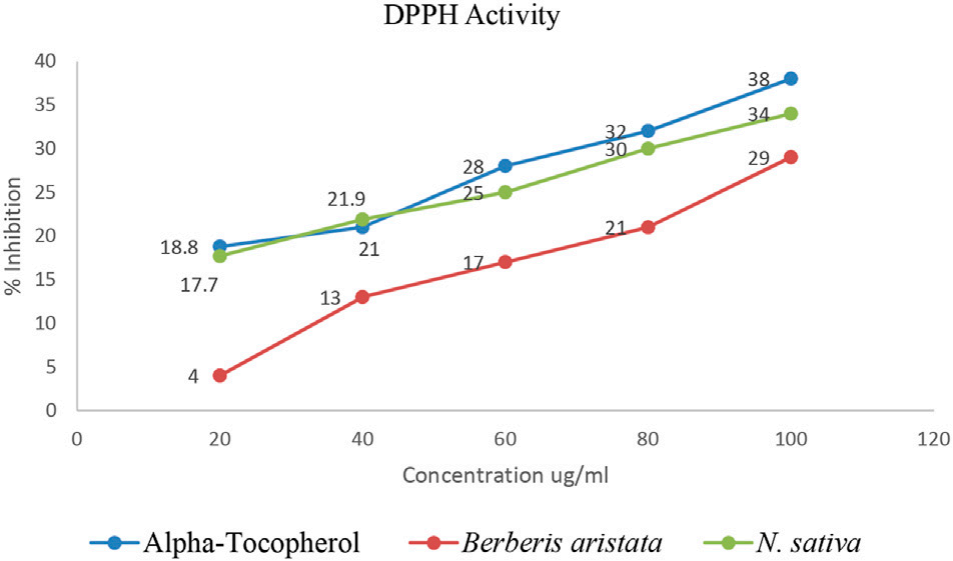
Percentage inhibition vs concentrations.

The antiradical activities of *B. aristata* and *N. sativa* and their radical forms are studied and results are presented in Table 2. DPPH has an absorption band at 515 nm which disappears upon reduction by an antiradical compound (Brand-Williams et al., 1995). Phytochemistry explained (Bors, 1996) that more phenolic groups were believed to produce more antioxidant activity. Many plant constituents scavenge free radicals. Figure 2 explains the potential of the extract in determining the significant antioxidant activity from natural sources. Demand for the natural antioxidants is shooting up nowadays as neutraceuticals, biopharmaceuticals, and food additives due to consumer preference. Dietary intake of antioxidant foods decreases the incidence of human diseases. Also, plant-based antioxidants are preferred against the synthetic ones because of their multiple mechanisms of actions and non-toxic nature. Our study used the bark of *B. aristata and N. sativa* that contains the berberine and thymoquinone active constituents, respectively, and other phytoconstituents including phenolic compounds.

**TABLE 4:** Inhibitory concentration (IC_50_) values of standards and test compounds.

S. No.	Name	IC_50_
1	Anastrazole	412.28 nM (±47.74)
2	Berberine Cl	686.49 nM (±161.46)
3	Thymoquinone	7.5 μg/ml (±0.202)
4	α-Pinene	6.72 μg/ml (±1.34)
5	β-Pinene	11.08 μg/ml (±0.63)
6	*B. aristata* extract	103.72 μg/ml (±54.17)
7	*Nigella sativa* extract	107.89 mg/ml (±12.05)
8	*Nigella sativa* oil	0.33% (±0.026)

Values are expressed in mean *n* = 3, SD (±).

### MTT Assay

**FIGURE 3:**
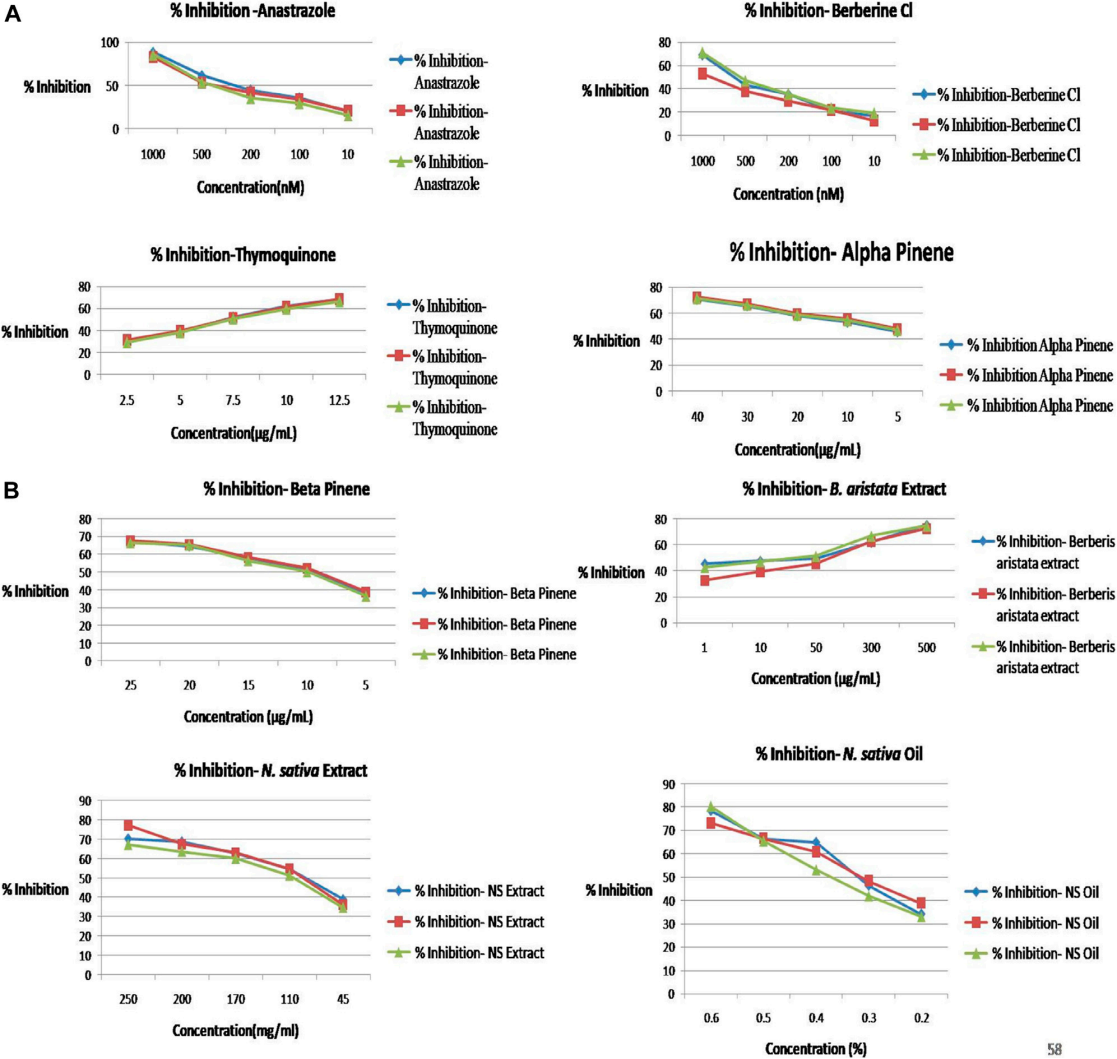
**(A)** Percentage inhibition vs concentration graphs. **(B)** Percentage inhibition vs concentration graphs.

As per the objective of the study, MTT assay were performed to find out the inhibitory concentration of various standards and test samples (*B. aristata* and *N. sativa* Extracts). The results are presented into graphical data Figure 3 and respective IC_50_ values expressed in Table 4 (sample *n* = 3 and standard deviation).

## Chromatography

### *B. aristata* Characterization

**FIGURE 4:**
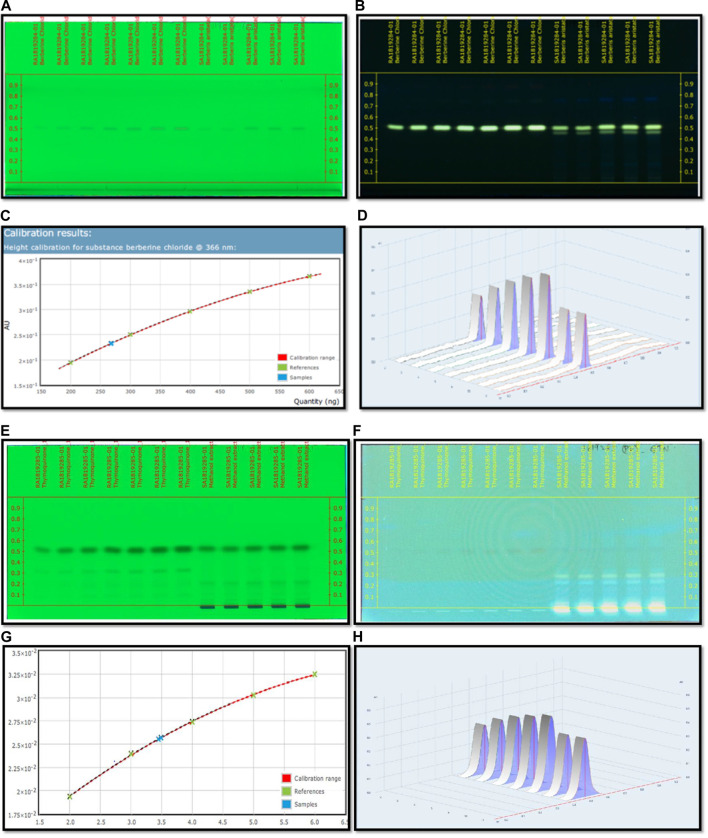
**(A)** HPTLC fingerprinting chromatogram of *Berberis aristata* at 254 nm. **(B)** HPTLC fingerprinting chromatogram of *Berberis aristata* at 366 nm. **(C)** Standard curve of thymoquinone using the polynomial regression mode. **(D)** Area under the curve in 3D of thymoquinone. **(E)** HPTLC fingerprinting chromatogram of *Nigella sativa* at 254 nm. **(F)** HPTLC fingerprinting chromatogram of *Nigella sativa* at 366 nm. **(G)** Standard curve of thymoquinone using the polynomial regression mode. **(H)** Area under the curve in 3D of thymoquinone.

Optimization of the HPTLC solvent system for quantitative analysis was done using a combination of solvent systems of varying polarity, and the most suitable solvent system was found to be n butanol:acetic acid:water (3:1:1) v/v/v. Quantitative analysis for berberine was performed through HPTLC techniques, and the result showed that the content of berberine in *B. aristata* was found to be 53.5 µg in 1 mg through HPTLC techniques compared to standard berberine Cl. The respective HPTLC chromatograms of bark extracts are presented in Figures 4A,B. In the present analysis, the calibration curve of standard berberine was found to be linear (y= −4.097 × 10^−13^ x^2^ + 7.5666 × 10^−7^x+5.943 × 10^−2^, R = 99.99), which is presented in Figure 4C. The interpretation of results suggests that the sample contained a considerable amount of berberine of 53.5 µg in 1 mg of the solution, which is approximately 5.4%.

### *Nigella sativa* L. Characterization

C_6_H_14_: CH_3_COOC_2_H_5_ (8:2)v/v used as a mobile phase for quantitative and qualitative analysis of thymoquinone in the *Nigella sativa* L. extract done using HPTLC methods, and the result showed that the presence of thymoquinone in *Nigella sativa* was found to be 17.3 mg per 10 g compared to the pure compound thymoquinone. The respective HPTLC chromatograms of thymoquinone and the herbal extract are shown in Figures 4E–F. During the present study, the calibration curve of standard thymoquinone was found to be linear (y = −3.761 × 10^−16^ x^2^ + 6.272 × 10^−9^x + 8.375 × 10^−3^, R= 99.98%), which is presented in Figure 4G. Graphical data of standard thymoquinone and the *N. sativa* extract are presented in Figure 4H. The percentage of thymoquinone detected was 0.17% w/w.

Results revealed the presence of several constituents in the extracts as evidenced with the chromatogram of the methanolic extract of *B. aristata* and *Nigella sativa*, which showed 10 peaks at different Rf values and the peak area at 366 nm in n butanol:acetic acid:water (3:1:1) v/v/v and n hexane: ethyl acetate (Ezeome and Anarado, 2007; Alhaj et al., 2008) v/v solvent systems, whereas peaks were present in the HPTLC fingerprinting chromatogram at 254 nm of both extracts. The number of constituents (No. of peaks-11) in the extract and their retention factors (Rf) and chromatographic profiles are shown in Figures 4A–D. The chromatogram of 26 compounds was obtained by GCMS analysis of NS (Table 5).

**TABLE 5:** Composition of *N. sativa* oil: integration peak, R time, area, and % area.

Peak	R. Time	Area	Area%	Name	CAS No. (FFNSC 2 Lib)
1	6.532	3,008,277	0.36	Thujene α	2867-05-2 (FFNSC 2 Lib)
2	6.813	75,532,375	8.93	Bicyclo[3.1.1]hept-2-ene, 2,6,6-trimethyl-80-56-8 (FFNSC 2 Lib)	
3	7.308	1,063,045	0.13	Camphene α	79-92-5 (FFNSC 2 Lib)
4	8.414	115,567,825	13.66	Pinene oxide β	6931-54-0 (FFNSC 2 Lib)
5	10.244	9,042,970	1.07	Cymene <para->	99-87-6 (FFNSC 2 Lib)
6	10.408	342,467	0.04	Cyclohexene, 1-methyl-4-(1-methylethenyl)-,(S)-	5989-54-8 (FFNSC 2 Lib)
7	13.368	4,033,463	0.48	3-Oxatricyclo[4.1.1.0(2,4)]octane, 2,7,7-trimethyl-	1686-14-2 (FFNSC 2 Lib)
8	13.904	2,122,686	0.25	Pinene oxide α	1686-14-2 (FFNSC 2 Lib)
9	14.261	1,263,376	0.15	(1R,4R,5S)-1-Isopropyl-4-methoxy-4-methylbicyclo[3.1.0]hexane	11001111-06-5(FFNSC 2 Lib) 11
10	15.158	365,669	0.04	Nopinone	24,903-95-5 (FFNSC 2 Lib)
11	15.330	2,293,068	0.27	Pinocarveol	5947-36-4 (FFNSC 2 Lib)
12	16.227	1,444,460	0.17	Pinocarvone	30,460-92-5 (FFNSC 2 Lib)
13	17.736	2,646,046	0.31	Myrtenal	564-94-3 (FFNSC 2 Lib)
14	18.319	835,255	0.10	Verbenone	80-57-9 (FFNSC 2 Lib)
15	20.305	6,423,268	0.76	Thymoquinone	6617-34-1 (NIST14 Lib)
16	22.575	568,936	0.07	Thujyl acetate <neoiso-3-> DB5-1081 (Classical name = neo-3-)	0-00-0(SZTERP Lib)
17	22.869	876,235	0.10	Pinocarveol <trans-> DB5-724	0-00-0(SZTERP Lib)
18	26.892	1,176,740	0.14	(+)-Longifolene	475-20-70(NIST14s Lib)
19	34.853	462,953	0.05	Dodecane	112-40-3 (FFNSC 2 Lib)
20	41.091	397,933	0.05	Tetradecanoic acid	544-63-8 (FFNSC 2 Lib)
21	48.341	48,096,480	5.69	Hexadecanoic acid	57-10-3 (FFNSC 2 Lib)
22	51.439	2,139,770	0.25	9,12-Octadecadienoic acid (Z,Z)-, methyl ester	56,599-58-7 (NIST14 Lib)
23	51.653	5,090,837	0.60	9-Undecenal, 2,10-dimethyl-	0—00-0 (NIST14 Lib)
24	54.211	560,235,582	66.24	Linoleic acid	506—21-8 (NIST14 Lib)
25	61.160	478,053	0.06	3-Cyclopentylpropionic acid, 2-dimethylaminoethyl ester	0-00-0 (NIST14 Lib)
26	65.858	278,309	0.03	Squalene	1111-02-4 (NIST14 Lib)
	Total	845,786,078	100.00		

The major compounds that could be identified were linoleic acid (66.2%), β-pinene (13.66%), hexadecanoic acid (5.69%), thymoquinone (0.76%), and α-pinene (0.25%). Until 2018, 39 clinical trials are recorded to berberine, and some of them are in phases II and III. The thymoquinone molecule has registered with 17 clinical trials, and nine have been completed with promising results; however, two new clinical trials were recruited until 2019. Toxicological and reference standards both together may evolve the new paradigm in treating the illness with herbal drugs. With the advancement of technology, it is evident that the herbal industry needs to follow strict guidelines, and such regulations are necessary for quality control and assurance to bring potential evidenced research on phytopharmaceuticals. Herbal drug regulations and their harmonization in Asia, Europe, and the United States may stimulate and strengthen phytopharmaceuticals by using the modern analytical techniques for standardization of marker compounds (Choudhary and Sekhon, 2011). As per WHO guidelines, 2007, quality of the herbal product needs to be standardized with respect to safety before releasing it into the market (15).

#### Mean Survival Time

**FIGURE 5:**
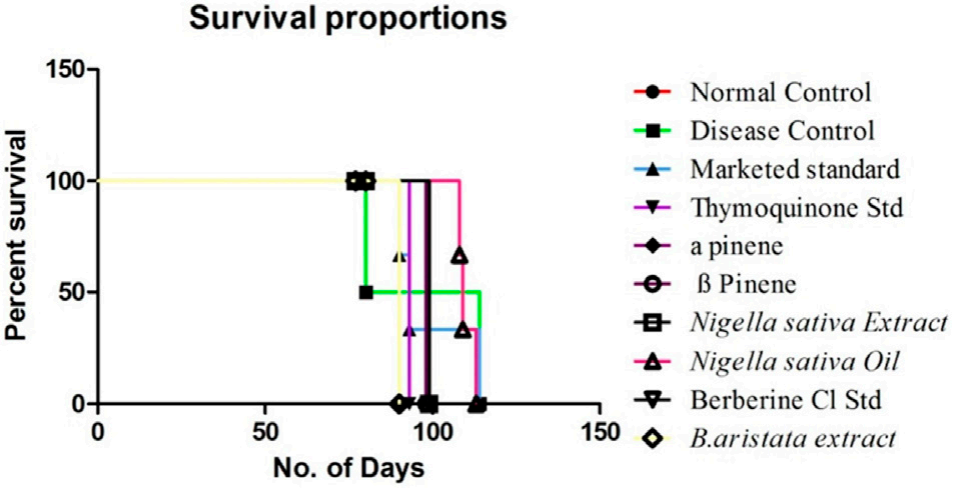
Effect of treatment on the MST using Kaplan Meier’s method.

A reliable criterion for judging the value of any anticancer drug is prolongation of the life span. Anastrazole showed the highest increase in life span of tumor-bearing animals and significantly increased the mean survival time (MST) to 112 days. Neither the BA extract nor the NS extract was not as effective as anastrazole and increased the MST to only 107 and 108 days; however, their marker compounds thymoquinone and berberine showed potential increment in MST. The higher rate of reduction in tumor volume indicated that *B. aristata* and *N. sativa* played a direct role in killing tumor cells and enhanced the curative effect of tumor chemotherapy. A decrease in tumor volume mentioned above reduced the tumor burden and might have enhanced the life span of the tumor-bearing mice (Figure 5) and increased the MST (16).

### Hematology

**FIGURE 6:**
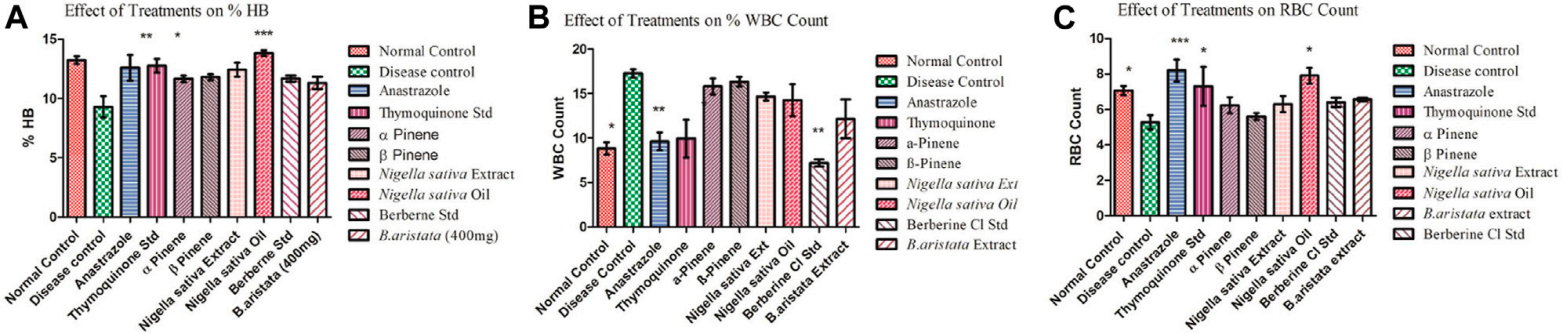
**(A–C)** Effect of *treatments* on the Hb count and WBC and RBC count (disease vs treatments *).

The elevated white blood cell (WBC) count in tumor-bearing mice was significantly reduced by anastrazole, thymoquinone, and berberine and relatively less reduced by the *B. aristata* and *N. sativa* extract treatments, which were significant (*p* < 0.05) and potentially reversed the tumor-induced rise in total counts of WBC (Figure 6B). However, the extracts were not as efficacious as anastrazole in reversing the tumor-induced total counts. The anastrazole, thymoquinone, and berberine treatments inhibit tumor cell growth, enhance the survival of treated mice, and restore the hematological parameters. Thus, the present study suggested that *B. aristata* and *N. sativa* possess potent antitumor activity and increase the life span of the treated animal. However *B. aristata* (400 mg/kg) shows more significant antitumor activity among all test extract groups in the animal study. Usually, in cancer chemotherapy, the major problems that are being encountered are myelosuppression and anemia, but the results have clearly shown that BS and NS treatments have brought back not only the hemoglobin content to normal but also the red blood cell (RBC) count to normal (Figure 6C). Anastrazole as well as the extracts reversed these changes significantly (*p* < 0.05). However, the extracts were less efficacious than anastrazole in their effects, but their respective marker compounds showed relatively good effects on the RBC count (*p* < 0.05). Tumor development in the animals caused significant anemia (a decrease in hemoglobin content), while anastrazole treatment reversed this significantly (*p* < 0.05). The treatments with α-pinene , β-pinene, and the *N. sativa* extract failed to correct the anemia (Figures 6A,C), but thymoquinone, berberine, and the *B. aristata* extract were found to be significant (*p* < 0.05). Analysis of the hematological parameters showed a minimum toxic effect in the mice, which were treated with both extracts (Figures 6A–C).

## Histology and Immunohistochemistry

**FIGURE 7:**
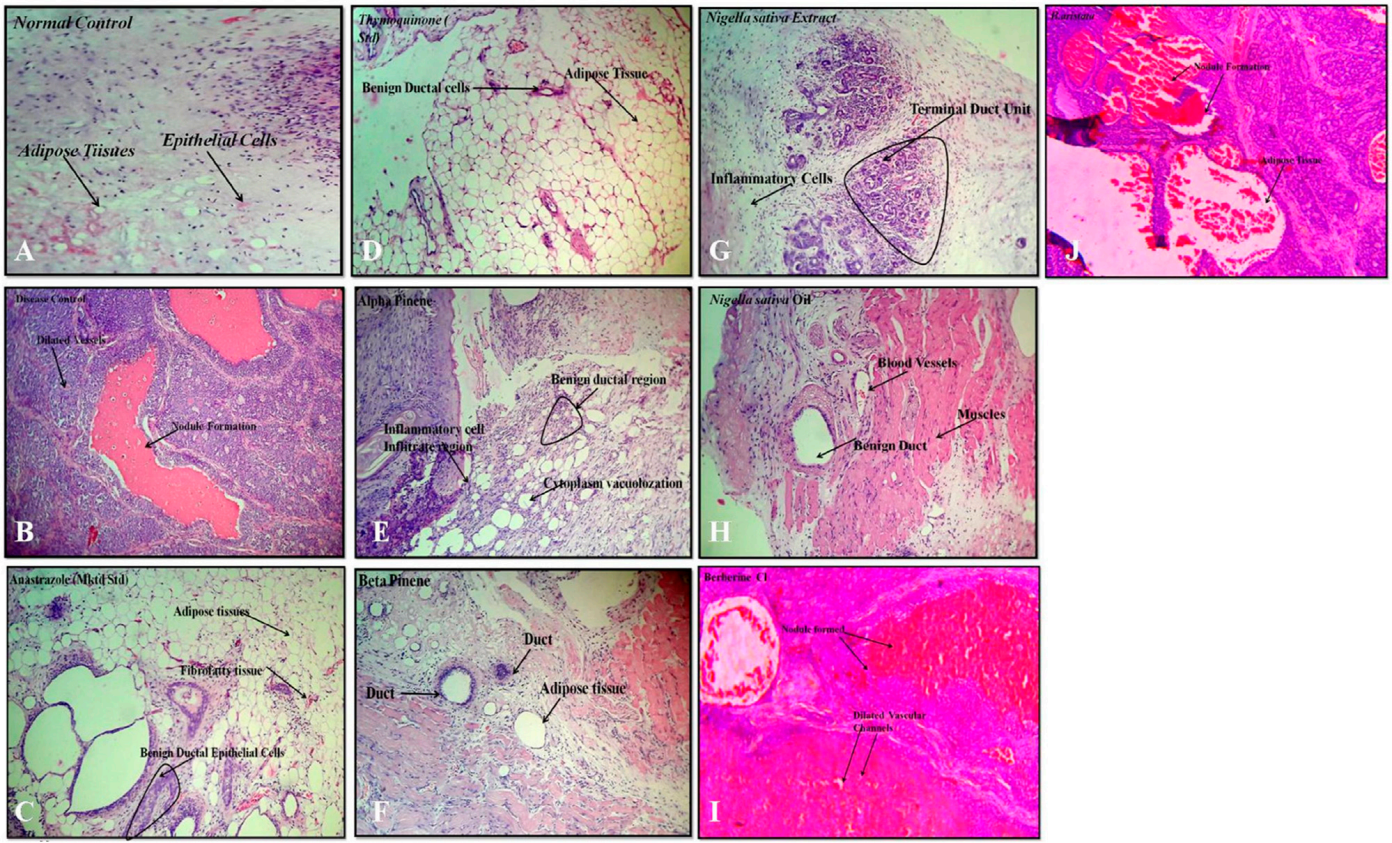
Histopathology: **(A)** normal control, **(B)** disease control, **(C)** marketed standard, **(D)** thymoquinone Std, **(E)** α-pinene Std, **(F)** β-pinene Std, **(G)** *N. sativa* extract, **(H)** *N. sativa* oil, **(I)** berberine Cl std, and **(J)** *Berberis aristata* extract.

A variety of herbal extracts were practiced for treating a number of diseases affecting the human body. Therefore, the objective of the current study was to evaluate the efficacy of *N. sativa* and *B. aristata* extracts on suppressing the several underlying tumor pathologies. For *in vivo* experiments, the DMBA mouse model was used to evaluate the protective effects of the lead herbal extracts (Russo et al., 2000). Active proliferation of the terminal ducts in the breast tissue for the mouse becomes very susceptible to carcinogens and tumor development (Ariazi et al., 2005). The histopathological examination of the current study revealed that DMBA-treated mice supplemented with TQ, the berberine NS extract, NS oil, and BA extracts showed suppression in the progress of the tumor cell proliferation (Figures 7D,G,H,J). In agreement with our findings (19), there was a limitation in the neoplastic changes during the sequential steps of carcinogenesis in DMBA-treated male Syrian hamsters supplemented with *N. sativa.* Another study found that the anticarcinogenic efficiency of *N. sativa* was found with the increase of glutathione-S-transferase activity (Aruna and Sivaramakrishnan, 1990). In line with these findings, the results of the current study found that NS oil and TQ were able to decrease the progress of tumors as well as decrease the values of TNF-α. Treatment with pure anastrazole caused partial necrosis of tumor tissues remaining in the form of “islands” (Figure 7C) and no effect observed with the vehicle-treated group, and a significant therapeutic effect was observed in tumor tissues after treatment with anastrazole, thymoquinone, and berberine (Figures 7C, D, I). We observed tumor cells with cytoplasm vacuolization and signs of karyolysis and inflammatory lymphocytic infiltration expressed weakly around the tumor (Figure 7B).

In our experiments, treatments with α- and β-pinene administered to mice did not induce changes in histology compared to the normal control group treated with the vehicle (Figures 7E–F). Protective effects could be explained by the ability of phytoconstituents (berberine) to inhibit the tumor cell target, which could account for the observed cancer protective potential of *B. aristata* that is pro-apoptotic and endowed with cell cycle arrest properties. However, anastrazole (1 mg/kg), berberine (30 mg/kg), and the *B. aristata* extract (400 mg/kg) against DMBA induced significantly improved tumor pathology. The ductal epithelial cells were bigger than normal cells and had an increased cytoplasm nucleus, which was observed in the group treated with the *B. aristata* extract.

### Immunohistochemistry

**FIGURE 8:**
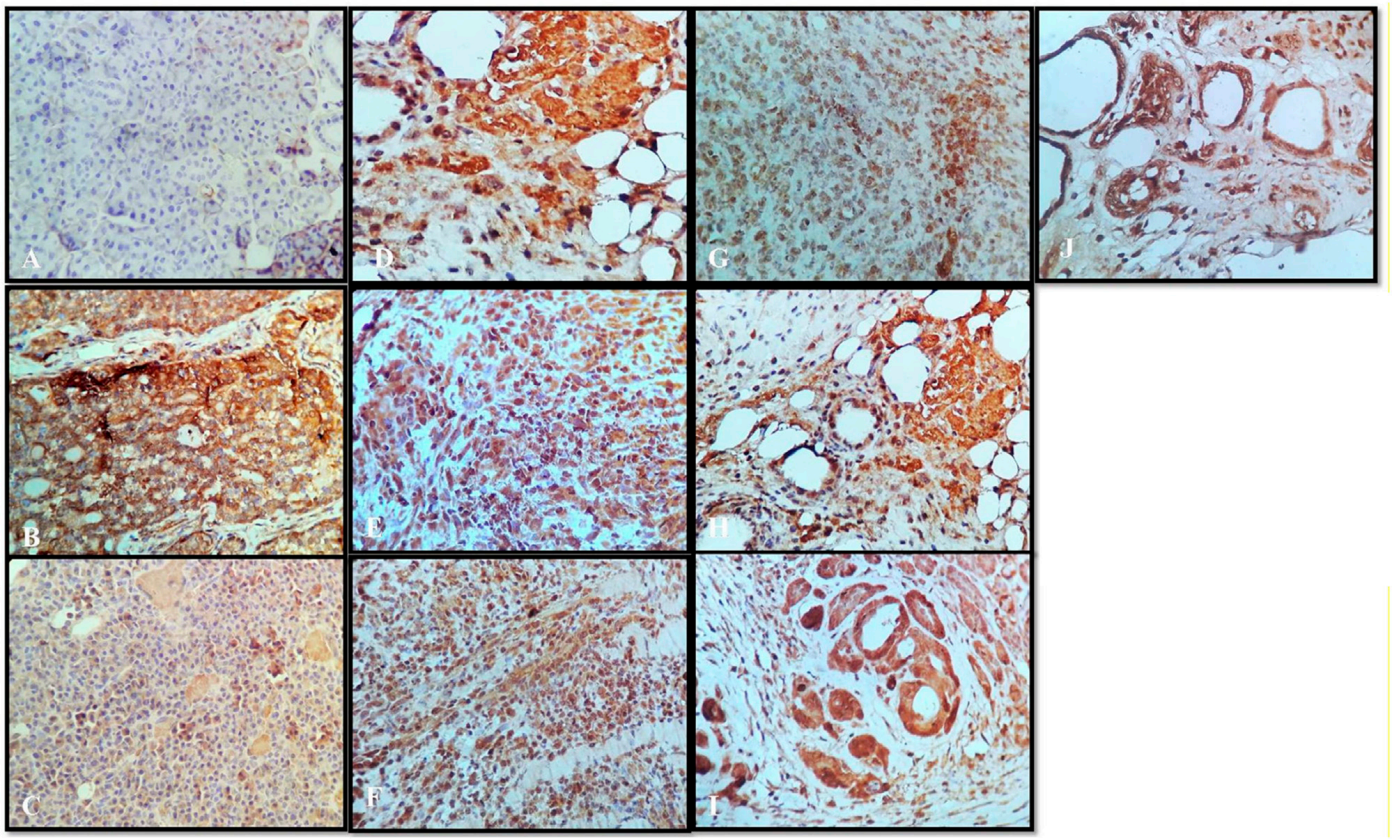
Immunochemistry TNF-α: **(A)** normal control, **(B)** disease control, **(C)** marketed standard, **(D)** thymoquinone Std, **(E)** α-pinene Std, **(F)** β-pinene Std, **(G)** *N. sativa* extract, **(H)** *N. sativa* oil, **(I)** berberine Cl Std, and **(J)** *Berberis aristata* extract.

Tumor necrosis factor alpha (TNF-α) is a master pro-inflammatory cytokine and multifunctional cytokine involved in apoptosis, inflammation, and immunity (21). TNF-α has been reported to be elevated in the serum of patients diagnosed with advanced stages and correlate with an increased number and size of metastatic sites (Berberoglu et al., 2004). Therefore, the TNF-TNFR2 axis was implicated in the suppression of immune response and affects tumor progression and metastasis (Mantovani et al., 2008). In the following sections, we will interpret a possible role of targeting TNF-α interactions with the herbal extract platform in the tumor. Positive staining for TNF-α was observed in all the groups (Figures 8B–J), except that the normal control group showed the negative expressions (Figure 8A). In the disease control group treated with the vehicle, the strong positive expressions of TNF-α were shown (Figure 8B); however, the group treated with anastrazole showed the least expression for TNF-α (Figure 8C) and thus an increase in MST. On comparing the group treated with the thymoquinone standard, α- and β-pinene with the *N. sativa* extract and NS oil were more promising and showed potency in reducing TNF-α, but NS oil has relatively less expression than the *N. sativa* extract. α- and β-pinene showed moderate TNF-α on comparing with the anastrazole-treated group.

Results depict that NS oil showed less TNF-α expression, that is, its phytoconstituent contained thymoquinone, and thus enabled an increase in the MST. The pure compound has better efficacy and potency among all the other standard compounds, and NS oil showed more efficacy due to the presence of thymoquinone. Now, comparing the standard treated group (Figure 8C) with pure berberine (30 mg/kg) and *B. aristata* (400 mg/kg) among test compounds, pure berberine showed (Figure 8I) moderately strong cytoplasmic positivity for TNF-α in the ductal epithelial cells; however, treatment with the *B. aristata* extract showed (Figure 8J) less strong cytoplasmic positivity for TNF-α. On the basis of TNF α expression, pure compounds have more antitumor potency against the extracts. The increased levels of TNF-α in the DMBA group, as compared to the control group, agree with the findings of other studies (Ardizzoia et al., 1992; Bower et al., 2002). This high level may be due to increased production by the tumor-infiltrating lymphocytes and/or by the tumor cells (Lind et al., 1993; Kopreski et al., 1996).

### Vascular Endothelial Growth Factor

**FIGURE 9:**
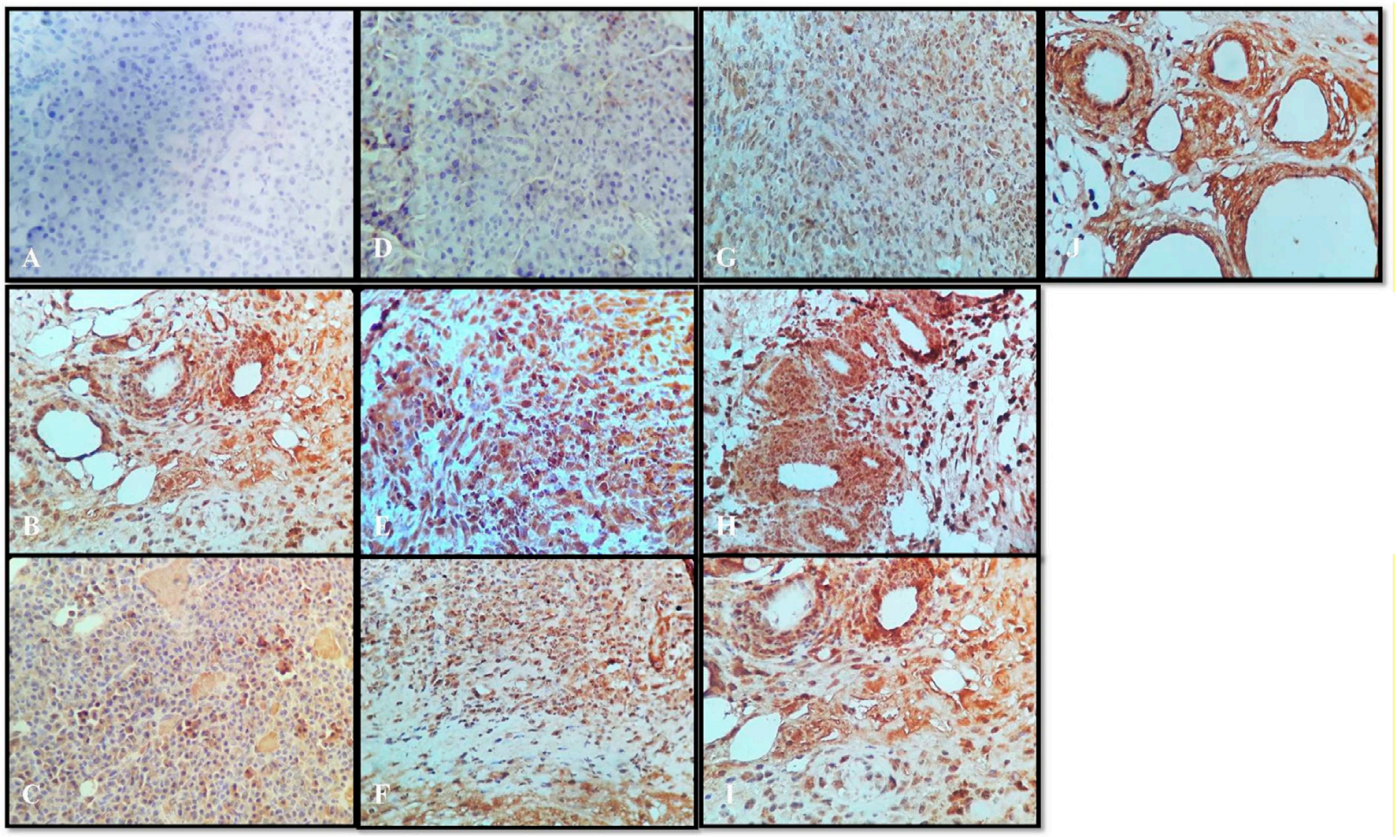
Immunochemistry VEGF: **(A)** normal control, **(B)** disease control, **(C)** marketed standard, **(D)** Thymoquinone Std, **(E)** α-pinene Std, **(F)** β-pinene Std, **(G)** *N. sativa* extract, **(H)** *N. sativa* oil, **(I)** berberine Cl Std, **(J)** *Berberis aristata* extract.

VEGF is a potent angiogenic factor and upregulated in many tumors, and its contribution to tumor angiogenesis is well defined. It is the key mediator of angiogenesis and binds two VEGF receptors (VEGF receptor-1 and VEGF receptor-2), which are expressed on vascular endothelial cells. Different agents including antibodies, aptamers, peptides, and small molecules have been extensively investigated to block VEGF and its pro-angiogenic functions. For multicellular tumor clones to grow beyond this size, they must recruit new blood vessels by angiogenesis and vasculogenesis. In the present study, all tumor disease controls (Figures 9B) to treatments (Figures 9C–J) were found to be VEGF-positive, which is in agreement with previous reports. Not only does VEGF have a direct influence on breast cancer invasion and migration but also it has been shown to act as a survival factor for metastatic breast carcinoma cells (Pidgeon et al., 2001). Studies in the laboratory have shown that VEGF protects both human and murine breast carcinoma cells from apoptosis (Chung et al., 2002). The standard group showed (Figure 9C) very weak cytoplasmic positivity for VEGF in the ductal epithelial cells on comparing with the disease control group (Figure 9B). This indistinct feature elaborates the potential role of VEGF in tumor pathogenesis with the past studies (Torres et al., 2016). Thymoquinone showed (Figure 9D) poor positive expression of VEGF in the ductal epithelial cells, which is similar to other studies that proved that thymoquinone attenuated tumorigenic signaling, including those controlled by EGF, FGF, VEGF, TGF, and various metastatic, angiogenic, and pro-mitogenic factors (Rashid et al., 2019). α-pinene (Figure 9E) showed moderately strong cytoplasmic positivity for VEGF in the ductal epithelial cells and β-pinene (Figure 9F) depicted moderately strong cytoplasmic positivity for VEGF in the ductal epithelial cells, which mean that these isomers have less affinity toward the VEGF receptors and thus are unable to cause the apoptotic action in tumor tissues. The *N. sativa* extract (Figure 9G) and NS oil (Figure 9H) show moderate cytoplasmic positivity for VEGF in the ductal epithelial cells; thus, the NS extract and NS oil have anti-VEGF potential in this regime (Zhang et al., 2014) and demonstrated that berberine inhibits the expression of hypoxia-inducible factor 1α and increases the expression level of VEGF in prostate cancer. Therefore, the anticancer effect of berberine hydrochloride on tumor cells may occur via the activation of the VEGF signaling pathway. The berberine Cl Std (Figure 9I) tissue shows moderately strong cytoplasmic positivity for VEGF in the ductal epithelial cells; however, the *B. aristata* extract (Figure 9J) depicted weak cytoplasmic positivity for VEGF.

## Conclusion

In conclusion, the results of the present study give clear evidence that the *N. sativa* extract and *B. aristata* extract and their marker compounds thymoquinone, ±pinene, and berberine Cl induce no harmful effects on female mice. Moreover, they exert a protective effect against the DMBA-induced tumor model. The antioxidant property is mediated by their actions and investigating other underlying mechanisms, merits, and further studies. The present study thus demonstrated the chemopreventive potential of thymoquinone, ± pinene and berberine Cl in the DMBA-induced tumor model. Despite the exact mechanism of action of chemopreventive potential of marker compounds, the antilipidperoxidative, antioxidant, and modulating effects on the detoxification cascade could play a possible role.

## Data Availability

The raw data supporting the conclusions of this article will be made available by the authors, without undue reservation.
