# Supplementary material for: Standardization of Berberis aristata DC and Nigella sativa L. Using HPTLC and GCMS and Their Antineoplasia Activity in 7,12-Dimethylbenz[a]anthracene-Induced Mouse Models
Source: Front Pharmacol. 2021 Nov 30;12:642067. doi: 10.3389/fphar.2021.642067 (PMC8670326; doi:10.3389/fphar.2021.642067)
Supplement: Supplementary file 1 [file DataSheet2.PDF]

101, A Wing, Aniket Apartment,
Navghar Road, Mulund (E),
Mumbai - 400 081, India.
Tel.: 091 22 21639928 - 31
E-mail : hptlc@anchrom.in
Web : www.anchrom.in

ANCHROM Enterprises (I) P. Ltd.
Technologists, not Traders!

Analysis: 1819285_20190304_(P2)

Path: Home/1819285

Based on method: 1819285

Created	04-Mar-2019 14:42:32	Lab_Sneha
Modified	05-Mar-2019 17:01:05	Saikat Mallick
Last HPTLC log	05-Mar-2019 17:04:34	File has been e-signed
Explorer notes		

Track	Vial ID	Description	Volume	Position	Type
1	RA1819285-01	Thymoquinone_1 mg/ml	1.0 µl	N/A	Reference
2	RA1819285-01	Thymoquinone_1 mg/ml	2.0 µl	N/A	Reference
3	RA1819285-01	Thymoquinone_1 mg/ml	3.0 µl	N/A	Reference
4	RA1819285-01	Thymoquinone_1 mg/ml	4.0 µl	N/A	Reference
5	RA1819285-01	Thymoquinone_1 mg/ml	5.0 µl	N/A	Reference
6	RA1819285-01	Thymoquinone_1 mg/ml	6.0 µl	N/A	Reference
7	RA1819285-01	Thymoquinone_1 mg/ml	7.0 µl	N/A	Reference
8	SA1819285-01	Methanol extract of Nigella Sativa	15.0 µl	N/A	Sample
9	SA1819285-01	Methanol extract of Nigella Sativa	15.0 µl	N/A	Sample
10	SA1819285-01	Methanol extract of Nigella Sativa	20.0 µl	N/A	Sample
11	SA1819285-01	Methanol extract of Nigella Sativa	20.0 µl	N/A	Sample
12	SA1819285-01	Methanol extract of Nigella Sativa	20.0 µl	N/A	Sample
Sequence table notes					

A track marked with ⚠ means: the application type is overridden in some evaluation(s).

System setup:

Software	Server VisionCATS-Server-PH, version 2.5.18262.1
Chamber	N/A
Linomat5	S/N:080222 (LINOMAT V)
Scanner4	S/N:170422 (SCANNER 4)
Visualizer	S/N:150503 (VISUALIZER)

Chromatography

Plate layout:

Stationary phase	Merck, TLC plates Al silica gel 60 F 254
Plate format	200.0 x 100.0 mm
Application type	BandIntertrack
Application	Position Y: 8.0 mm, length: 8.0 mm, width: 0.0 mm
Track	First position X: 20.0 mm, distance: 14.0 mm
Solvent front position	70.0 mm
Notes	

Take image clean plate 1a - Visualizer (S/N: 150503):

Quality	Enhanced
R White	auto capture, Auto, level 85 %, Band
R 254	auto capture, Auto, level 85 %, Band
R 366	auto capture, Auto, level 85 %, Band
Instrument diagnostics	Valid diagnostics
Documentation step label	
Notes	

Application 1 - Linomat 5 (S/N: 080222):

101, A Wing, Aniket Apartment,
Navghar Road, Mulund (E),
Mumbai - 400 081, India.
Tel.: 091 22 21639928 - 31
E-mail : hptlc@anchrom.in
Web : www.anchrom.in

Sample solvent type	Methanol
Dosage speed	150 nl/s
Pre dosage volume	0.20 ul
Instrument diagnostics	Valid diagnostics
Notes	

Development 1 - Chamber:

Tank	TTC 20x10
Mobile phase	n hexane:ethyl aceate(8:2)v/v
Saturation time	20 min
Use saturation pad	true
Use smartALERT	false
Volume front through	10 ml
Volume rear through	10 ml
Drying time	5 min
Drying temperature	Room temperature
Notes	

Take image developed plate 1a - Visualizer (S/N: 150503):

Quality	Enhanced
R White	auto capture, Auto, level 85 %, Band
R 254	auto capture, Auto, level 85 %, Band
R 366	auto capture, Auto, level 85 %, Band
Instrument diagnostics	Valid diagnostics
Documentation step label	
Notes	

Scan developed plate 1b - Scanner 4 (S/N: 170422):

Scanner type	Single λ
Optimization for	Light (sensitivity)
Measurement mode	Absorption
Filter	n/a
Detector mode	Automatic
Scanning speed	20 mm/s
Data resolution	25 µm/step
Slit	6 x 0.45 mm, micro
Partial scan	No
Lamp	Deuterium
Wavelength(s)	254 nm
Instrument diagnostics	Valid diagnostics
Documentation step label	
Notes	

Spectrum Scan developed plate 1c - Scanner 4 (S/N: 170422):

Scanner type	Spectrum
Optimization for	Light (sensitivity)
Measurement mode	Absorption
Filter	n/a
Detector mode	Automatic
Spectrum speed	20 nm/s
Data resolution	1 nm
Slit	6 x 0.45 mm, micro
Lamp	Deuterium
Wavelength range	190 nm to 450 nm
Reference spectrum	Per plate, X=10.0 mm, Y=10.0 mm
Purity	No
Instrument diagnostics	Valid diagnostics
Documentation step label	
Notes	

Substance Thymoquinone (Rf. 0.503 +/- 0.029):

Track	Rf	X (mm)	Y (mm)
1	0.529	20.0	40.8
2	0.519	34.0	40.1
3	0.518	48.0	40.1
4	0.516	62.0	40.0
5	0.509	76.0	39.5
6	0.510	90.0	39.6
7	0.514	104.0	39.9
8	0.514	118.0	39.9
9	0.508	132.0	39.5
10	0.509	146.0	39.5
11	0.509	160.0	39.5
12	0.508	174.0	39.5

System suitability tests:

SST settings:

SST tracks	

Data acquisition

Application 1 - Linomat 5 (S/N: 080222):

Executed	04-Mar-2019 16:26:49 Lab_Sneha

Development 1 - Chamber:

Executed	04-Mar-2019 16:54:43 Lab_Sneha

Take image developed plate 1a - Visualizer (S/N: 150503):

Executed	04-Mar-2019 17:14:49 Lab_Sneha

R White Developed, RemissionVis

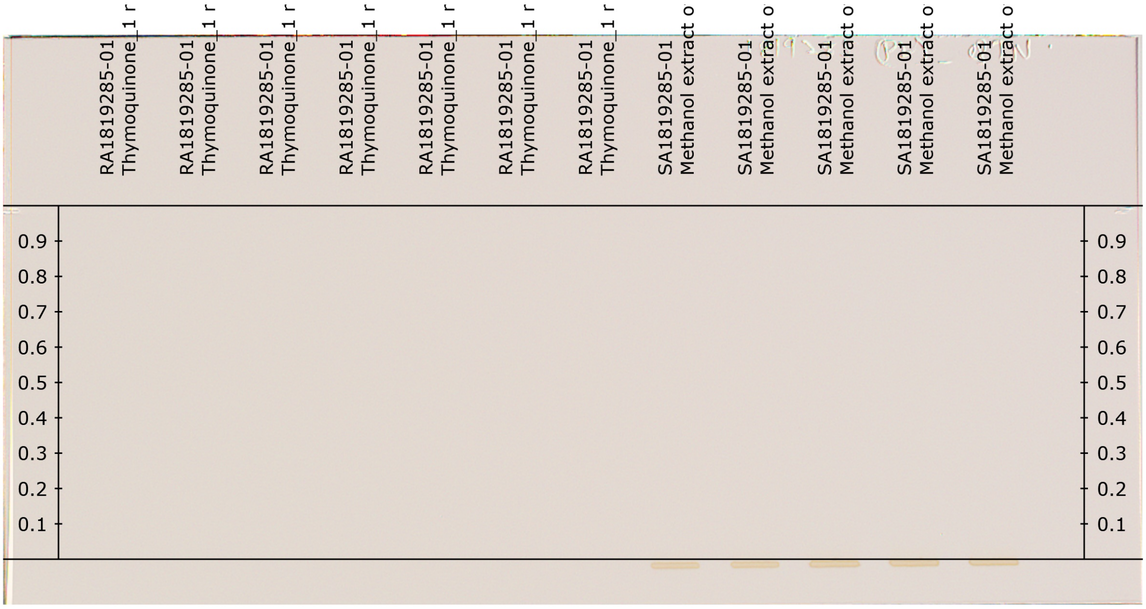

Exposure	0.077 s
Contrast	1
Normalized exposure	Disabled
Clarify	Disabled
White balance	1.00, 1.00, 1.00

R 254 Developed, Remission254

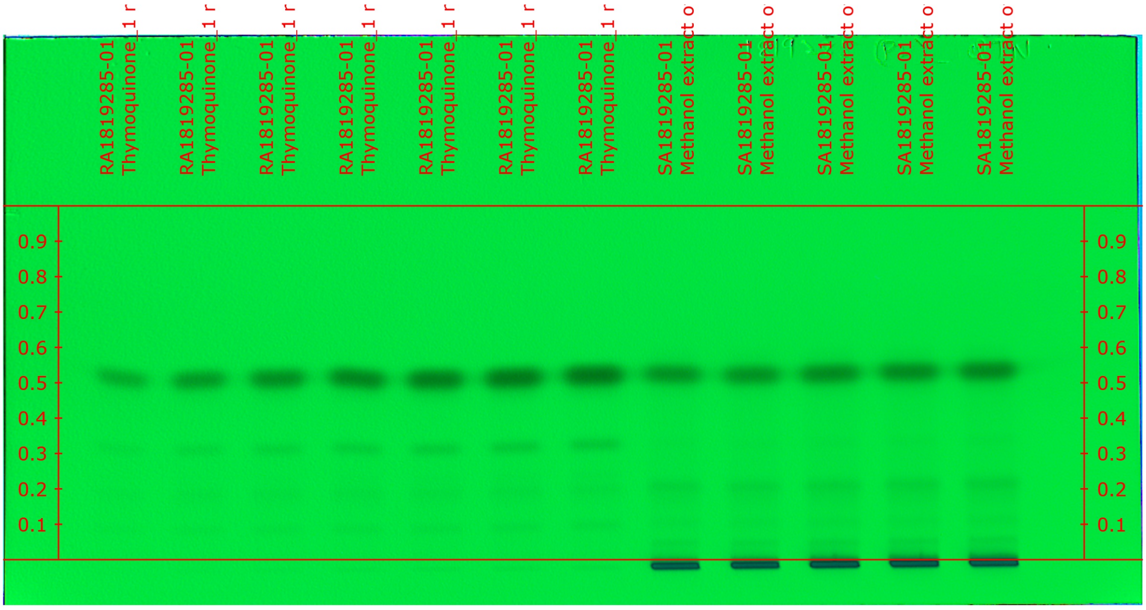

Exposure	0.336 s
Contrast	1
Normalized exposure	Disabled
Clarify	Disabled
White balance	1.00, 1.00, 1.00

101, A Wing, Aniket Apartment,
Navghar Road, Mulund (E),
Mumbai - 400 081, India.
Tel.: 091 22 21639928 - 31
E-mail : hptlc@anchrom.in
Web : www.anchrom.in

R 366 Developed, Remission366

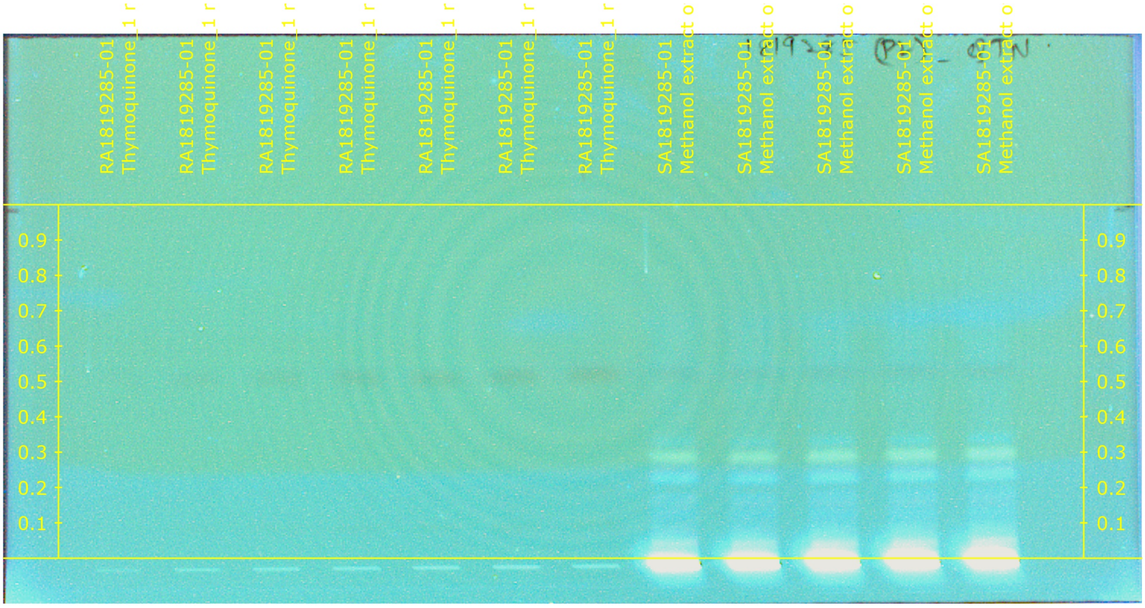

Exposure	0.997 s
Contrast	1
Normalized exposure	Enabled
Clarify	Disabled
White balance	1.00, 1.00, 1.00

Scan developed plate 1b - Scanner 4 (S/N: 170422):

Executed	04-Mar-2019 17:18:43 Lab_Sneha

Scan:

Wavelength	254 nm

Track 1:

Type	Single λ

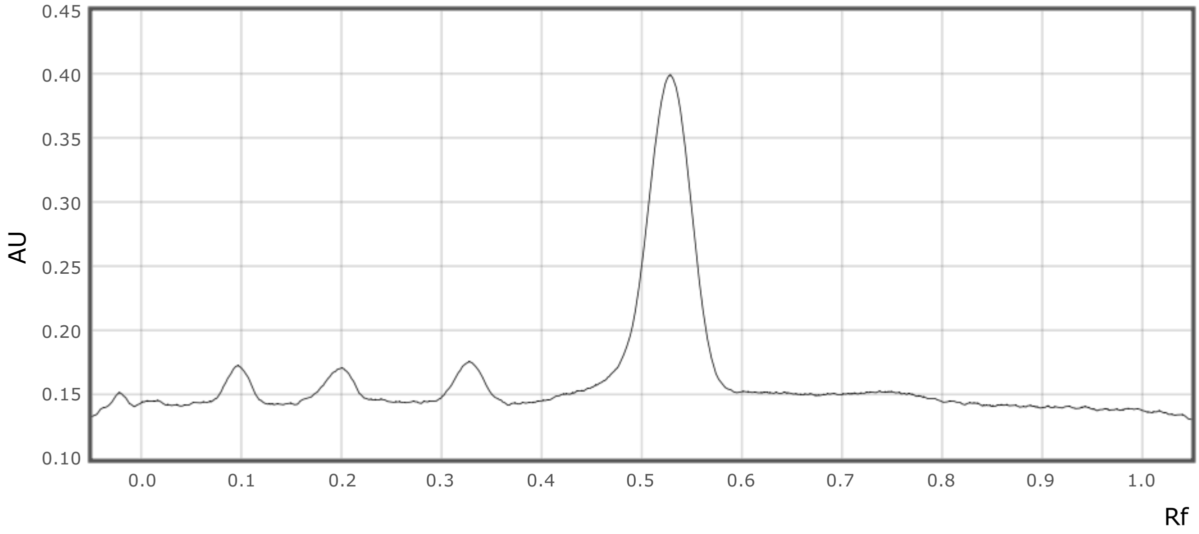

Track 2:

Type	Single λ

101, A Wing, Aniket Apartment,
Navghar Road, Mulund (E),
Mumbai - 400 081, India.
Tel.: 091 22 21639928 - 31
E-mail : hptlc@anchrom.in
Web : www.anchrom.in

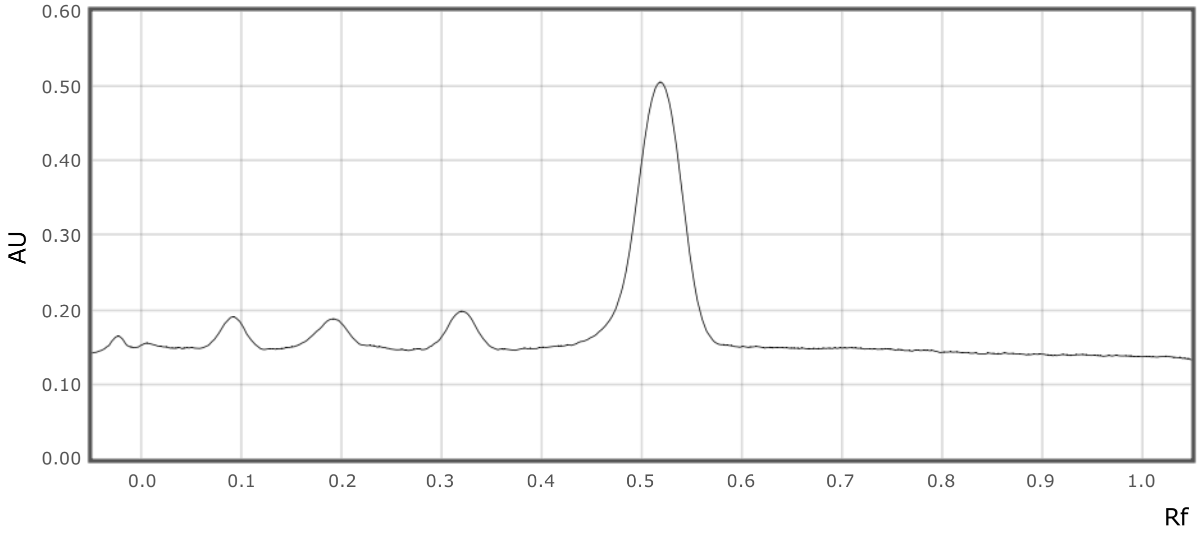

Track 3:

Type	Single λ

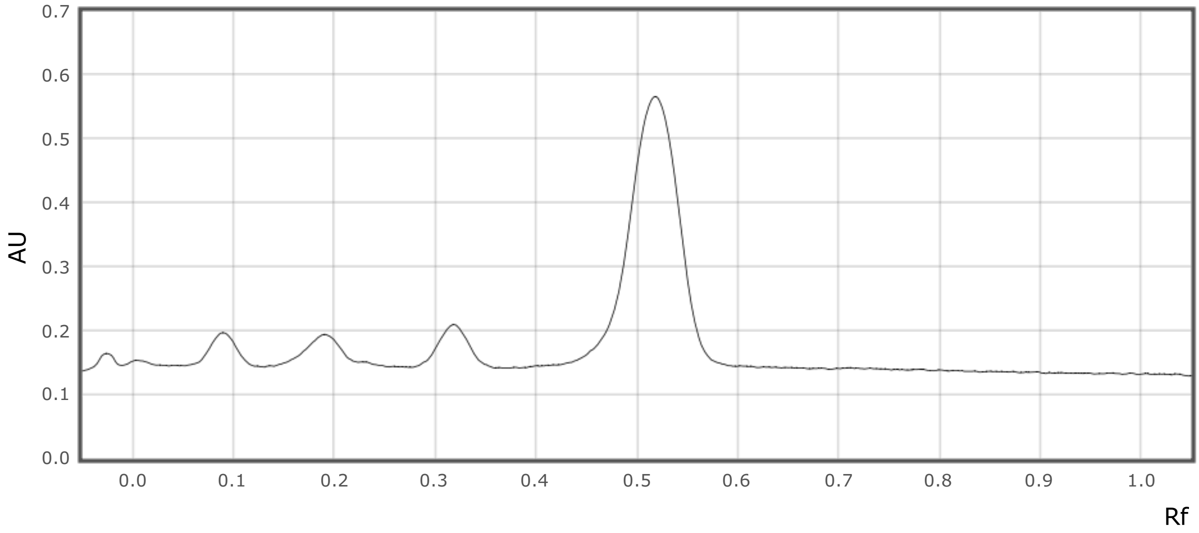

Track 4:

Type	Single λ

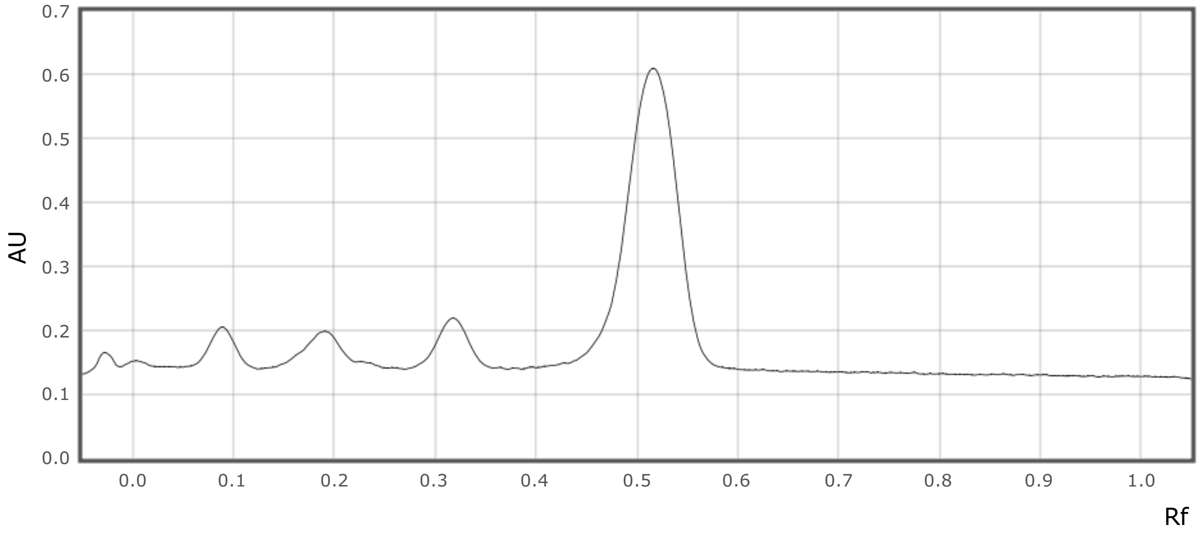

101, A Wing, Aniket Apartment,
Navghar Road, Mulund (E),
Mumbai - 400 081, India.
Tel.: 091 22 21639928 - 31
E-mail : hptlc@anchrom.in
Web : www.anchrom.in

Track 5:

Type	Single λ

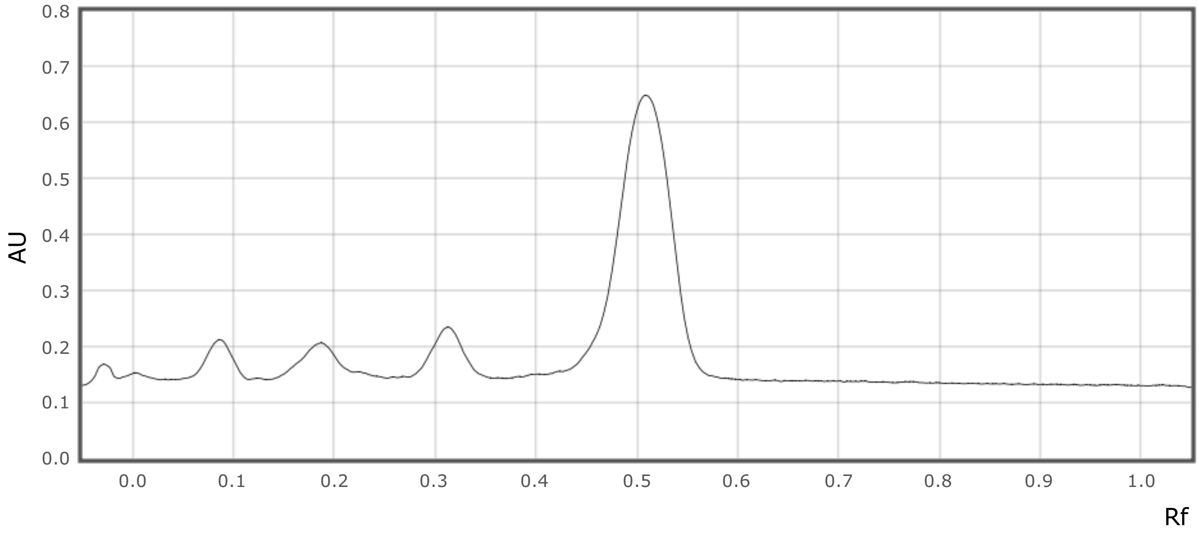

Track 6:

Type	Single λ

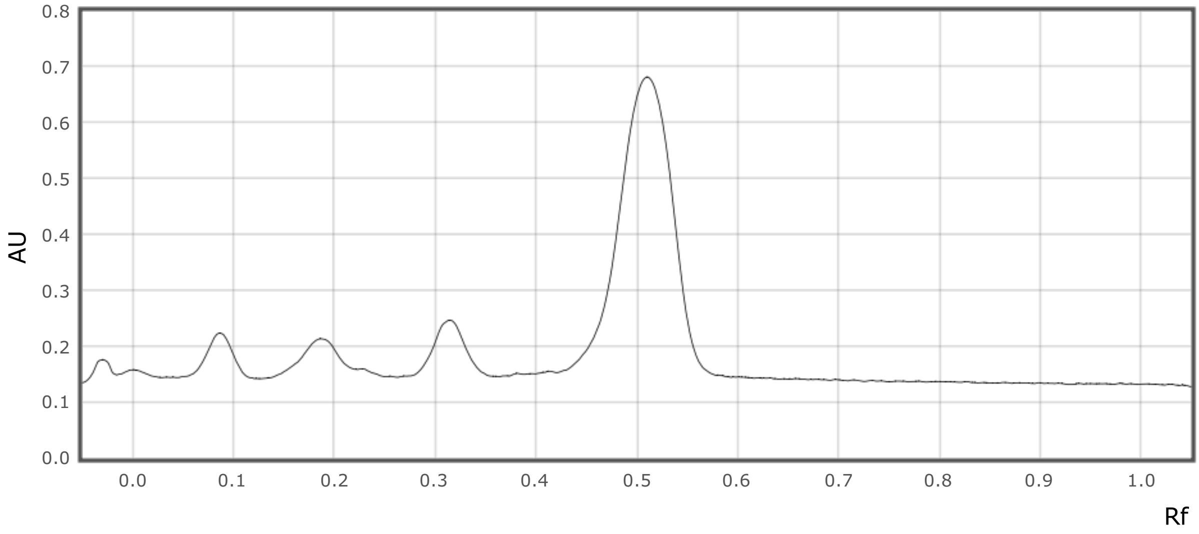

Track 7:

Type	Single λ

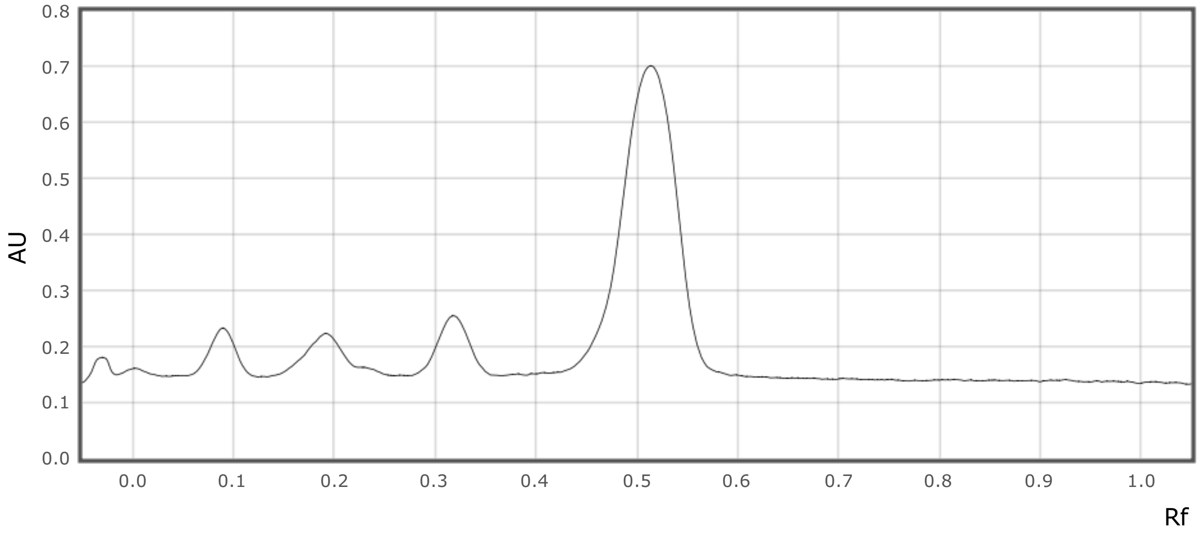

Track 8:	
Type	Single λ

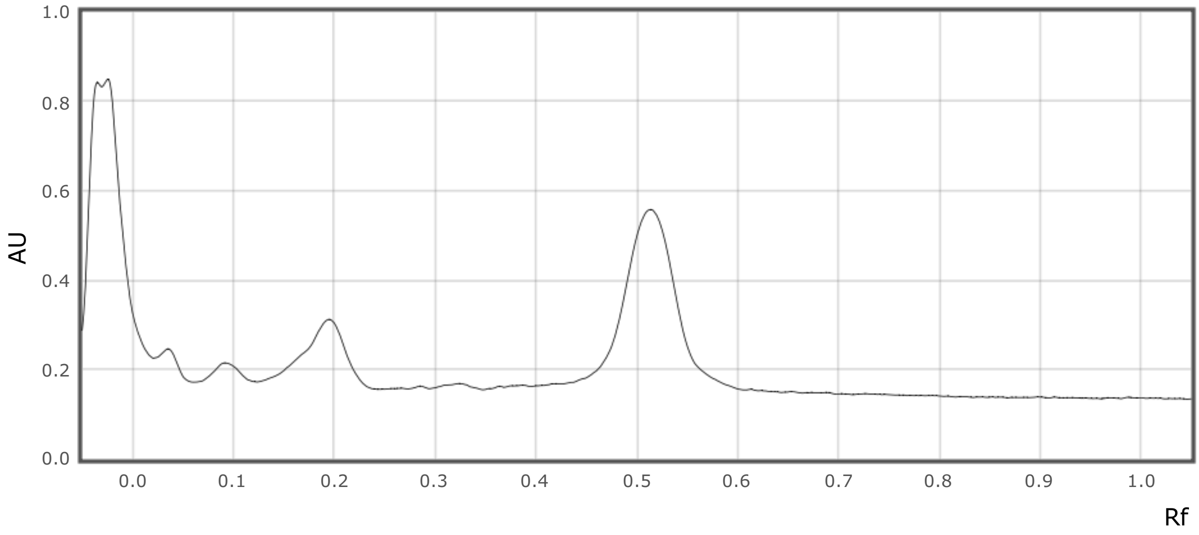

Track 9:	
Type	Single λ

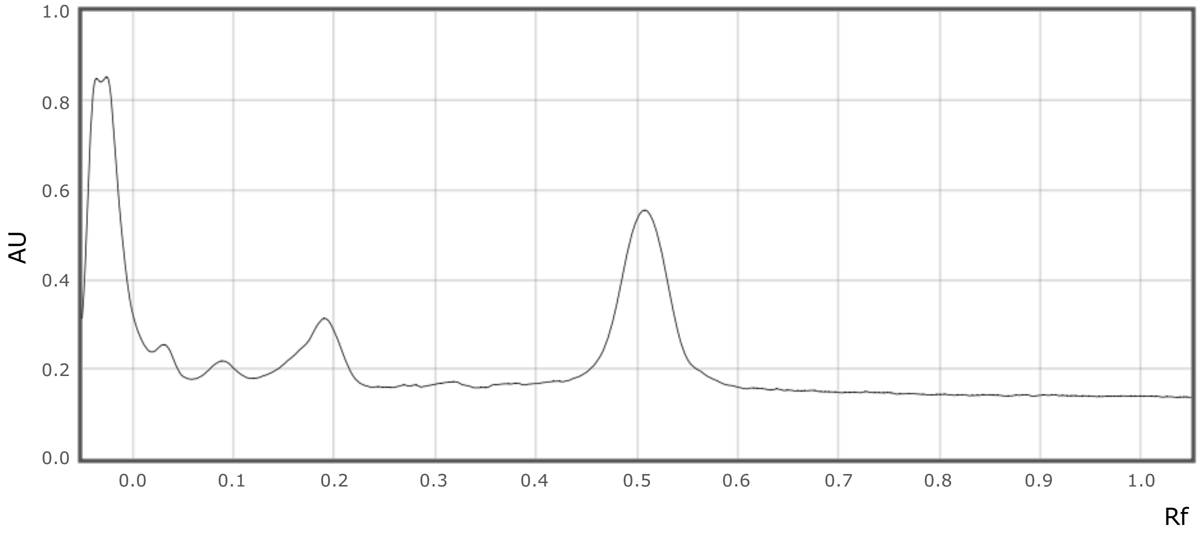

101, A Wing, Aniket Apartment,
Navghar Road, Mulund (E),
Mumbai - 400 081, India.
Tel.: 091 22 21639928 - 31
E-mail : hptlc@anchrom.in
Web : www.anchrom.in

Track 10:

Type	Single λ

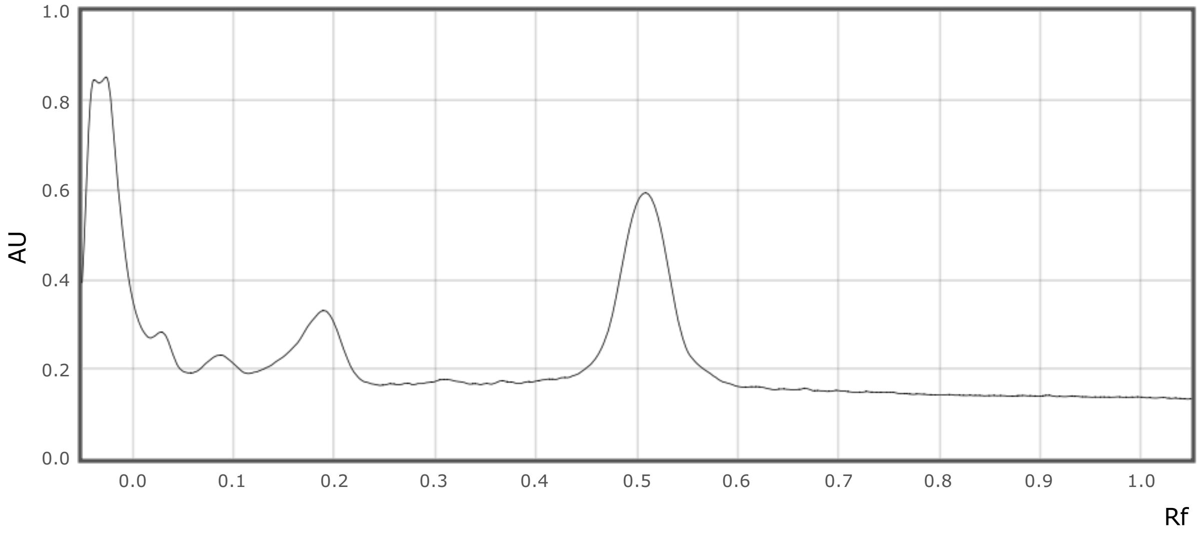

Track 11:

Type	Single λ

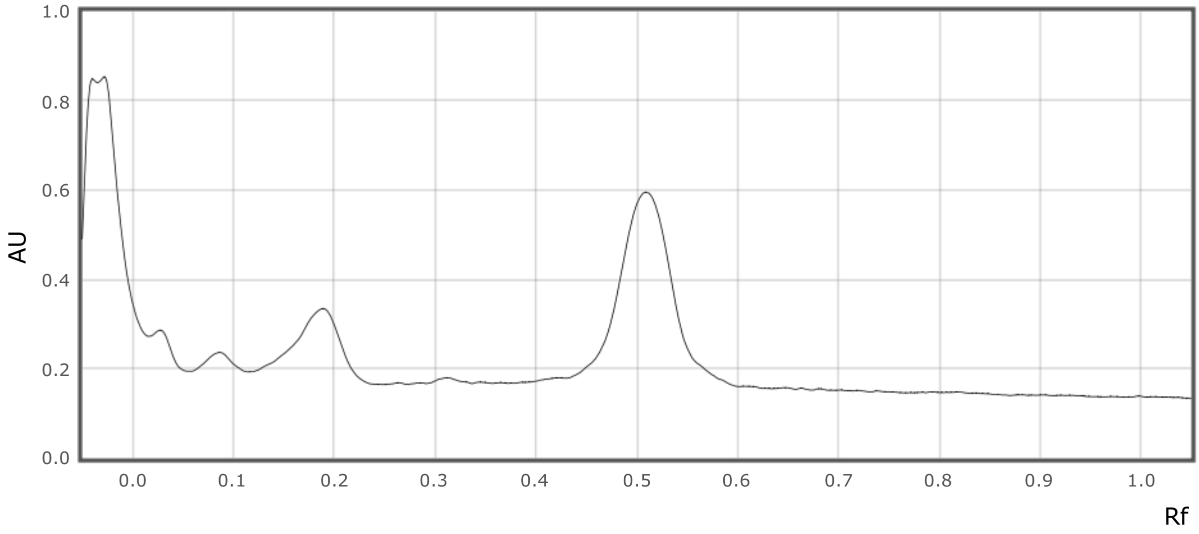

Track 12:

Type	Single λ

101, A Wing, Aniket Apartment,
Navghar Road, Mulund (E),
Mumbai - 400 081, India.
Tel.: 091 22 21639928 - 31
E-mail : hptlc@anchrom.in
Web : www.anchrom.in

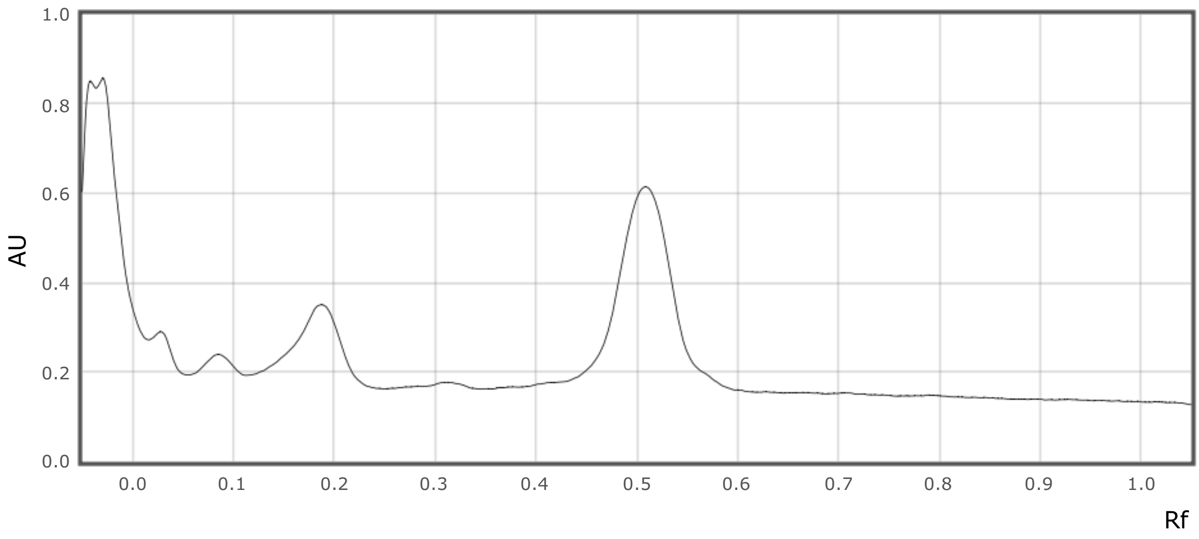

Spectrum Scan developed plate 1c - Scanner 4 (S/N: 170422):

Executed	04-Mar-2019 17:36:21 Lab_Sneha

Substance Thymoquinone (Rf. 0.503 +/- 0.029):

Tr. 1 Rf 0.529 (20.0 mm, 40.8 mm)

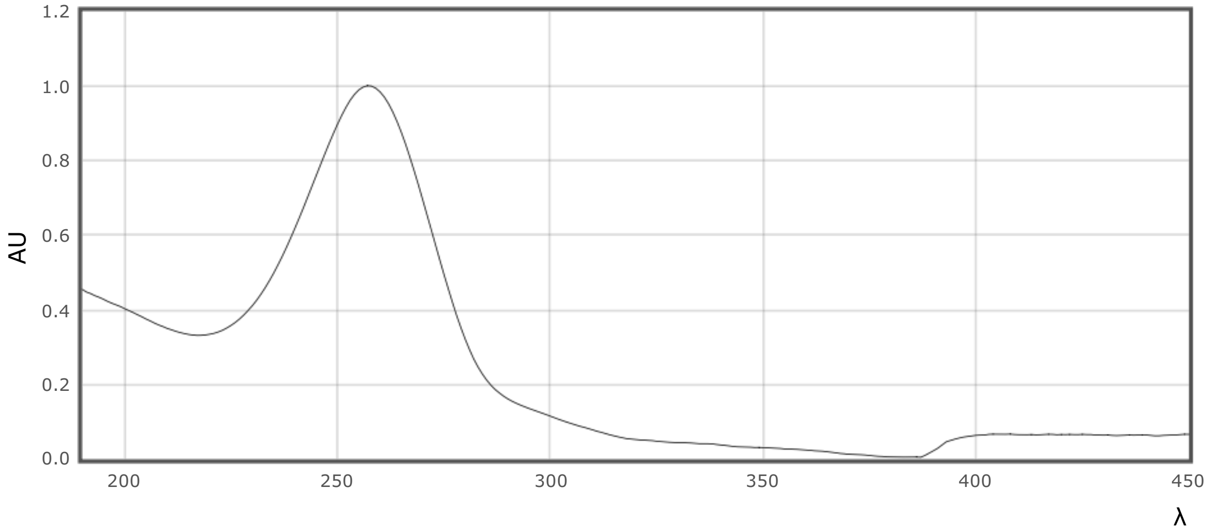

Tr. 2 Rf 0.519 (34.0 mm, 40.1 mm)

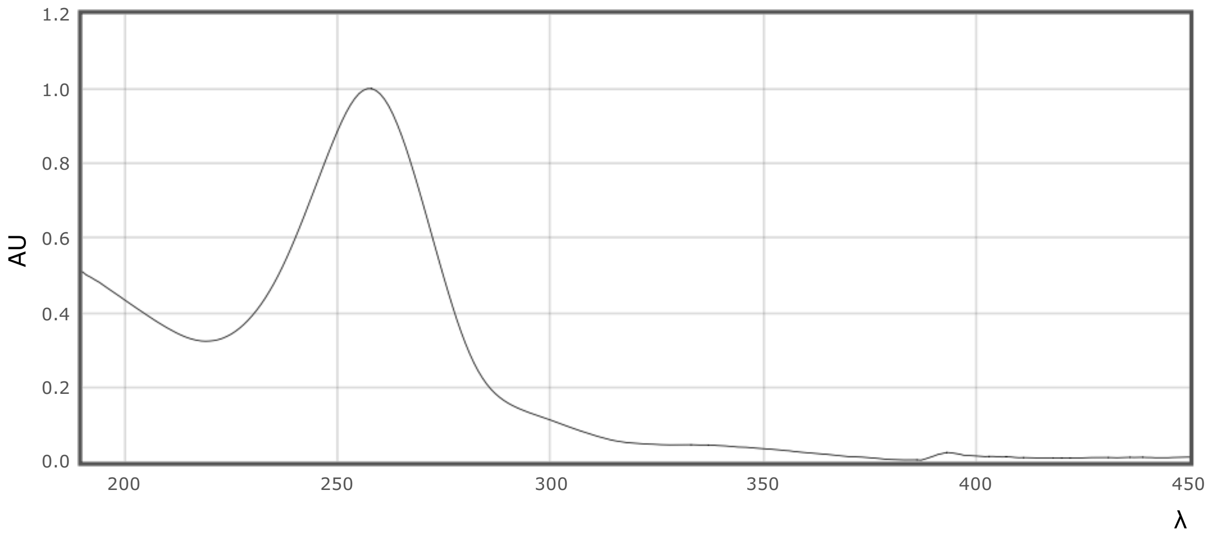

Tr. 3 Rf 0.518 (48.0 mm, 40.1 mm)

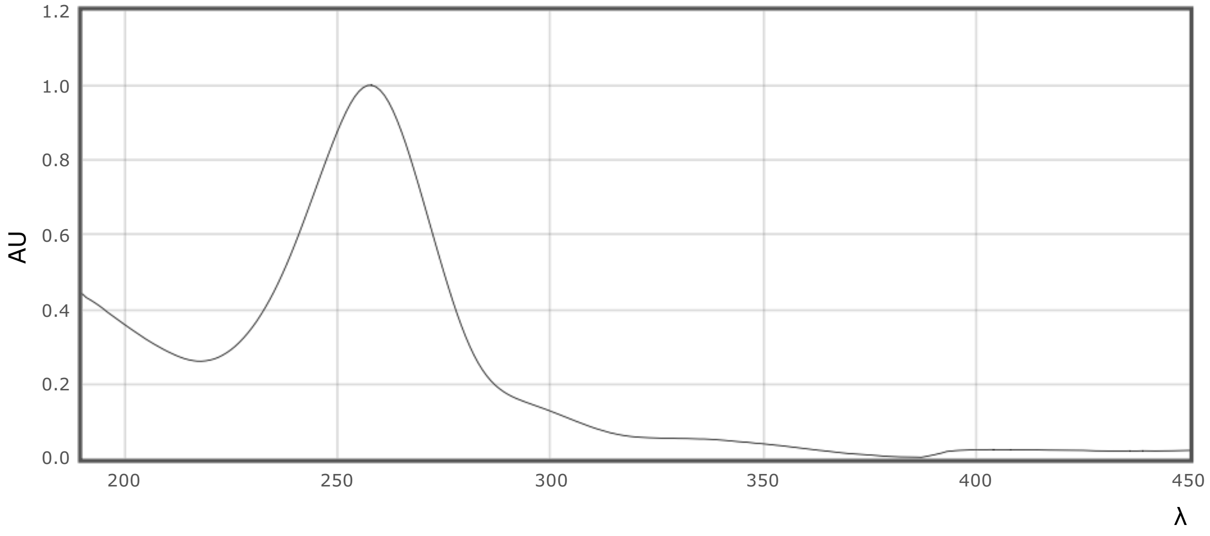

Tr. 4 Rf 0.516 (62.0 mm, 40.0 mm)

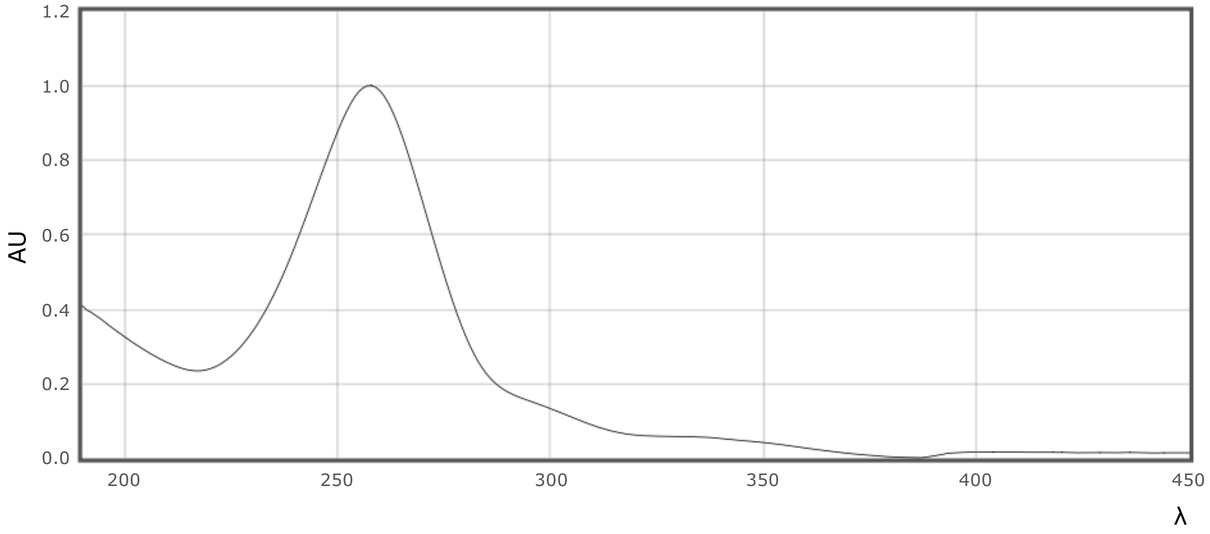

Tr. 5 Rf 0.509 (76.0 mm, 39.5 mm)

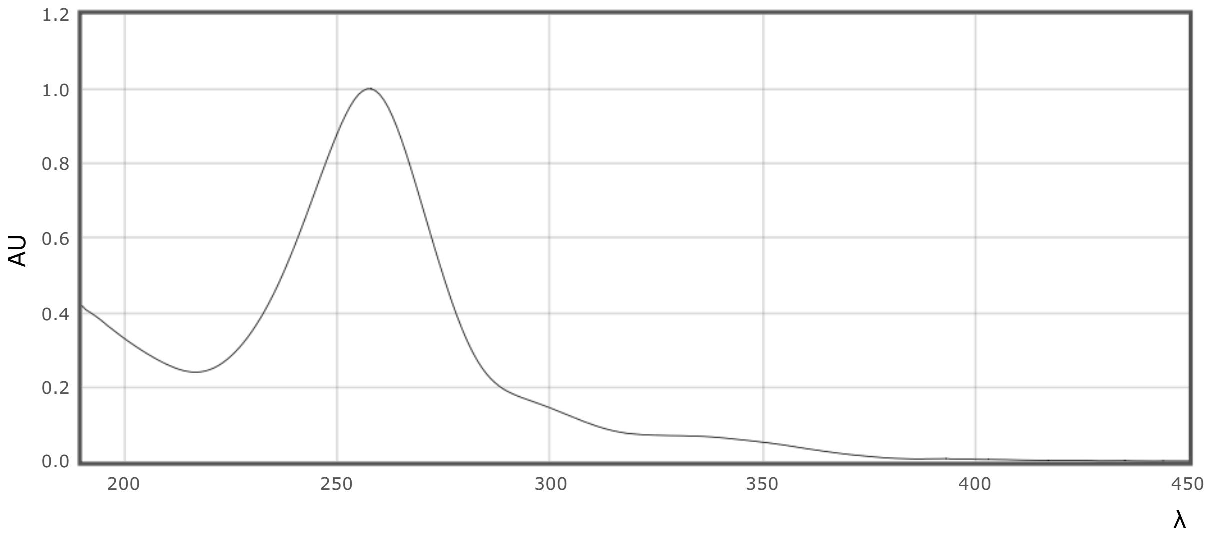

Tr. 6 Rf 0.510 (90.0 mm, 39.6 mm)

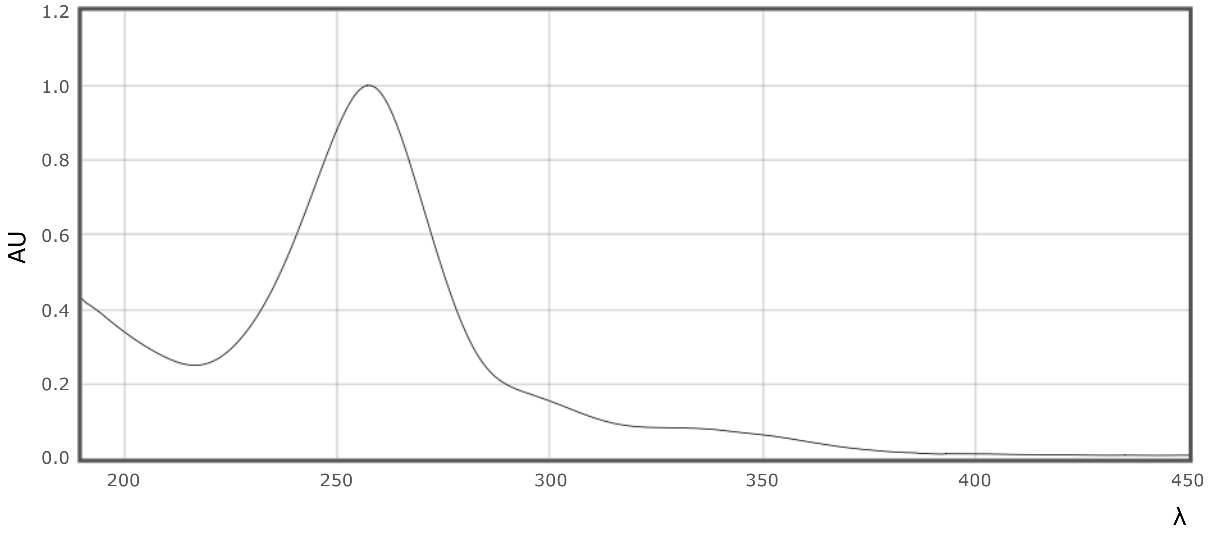

Tr. 7 Rf 0.514 (104.0 mm, 39.9 mm)

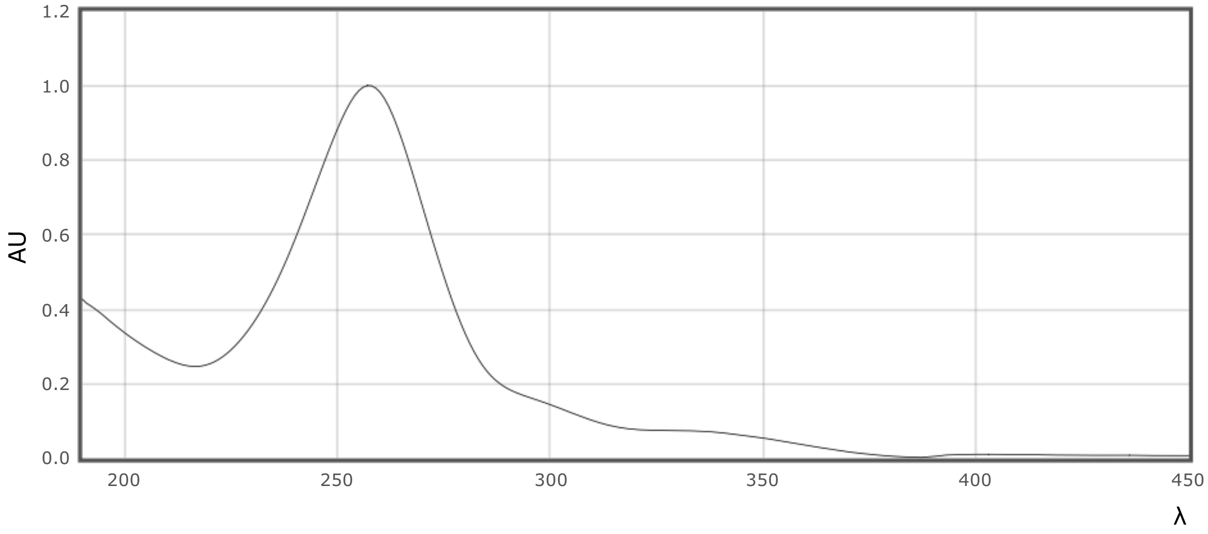

Tr. 8 Rf 0.514 (118.0 mm, 39.9 mm)

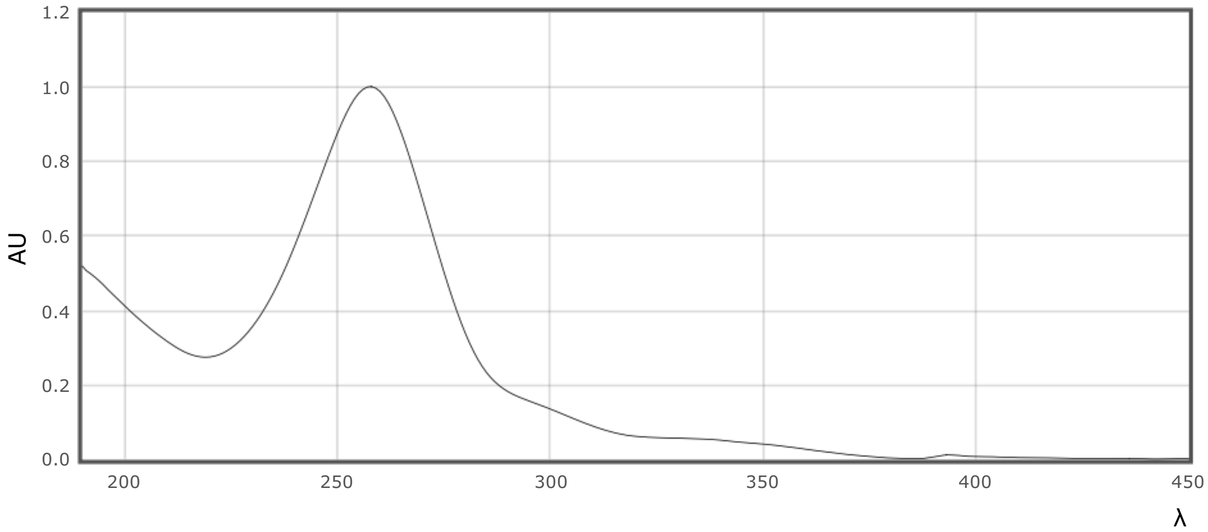

Tr. 9 Rf 0.508 (132.0 mm, 39.5 mm)

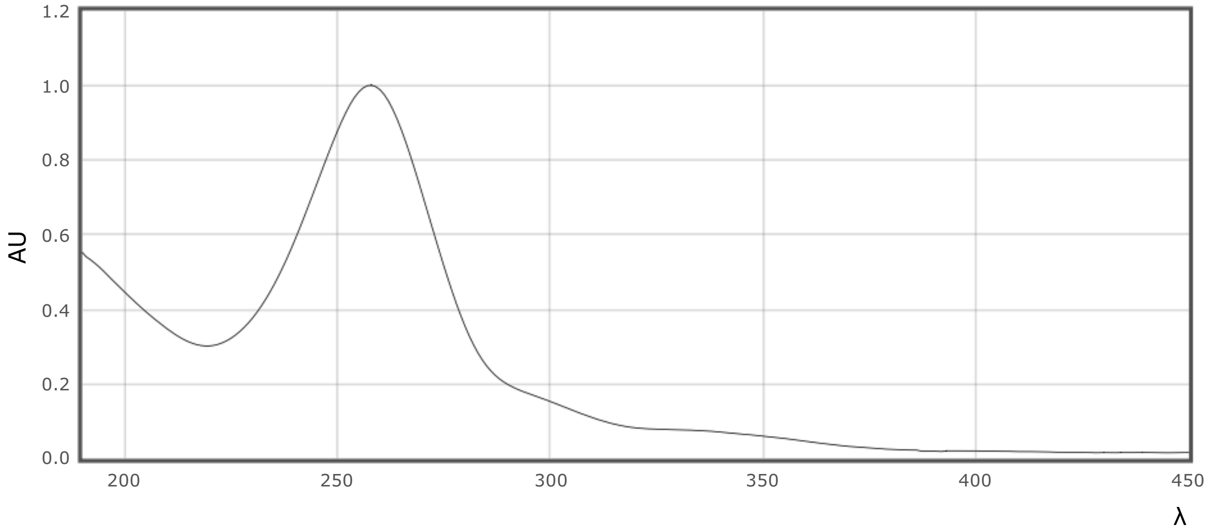

Tr. 10 Rf 0.509 (146.0 mm, 39.5 mm)

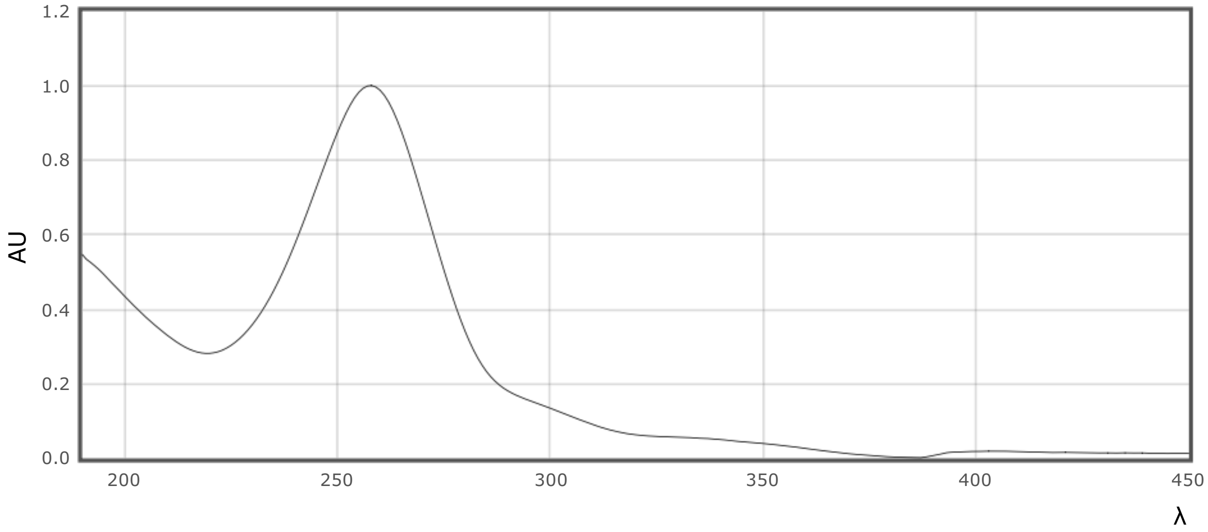

Tr. 11 Rf 0.509 (160.0 mm, 39.5 mm)

101, A Wing, Aniket Apartment,
Navghar Road, Mulund (E),
Mumbai - 400 081, India.
Tel.: 091 22 21639928 - 31
E-mail : hptlc@anchrom.in
Web : www.anchrom.in

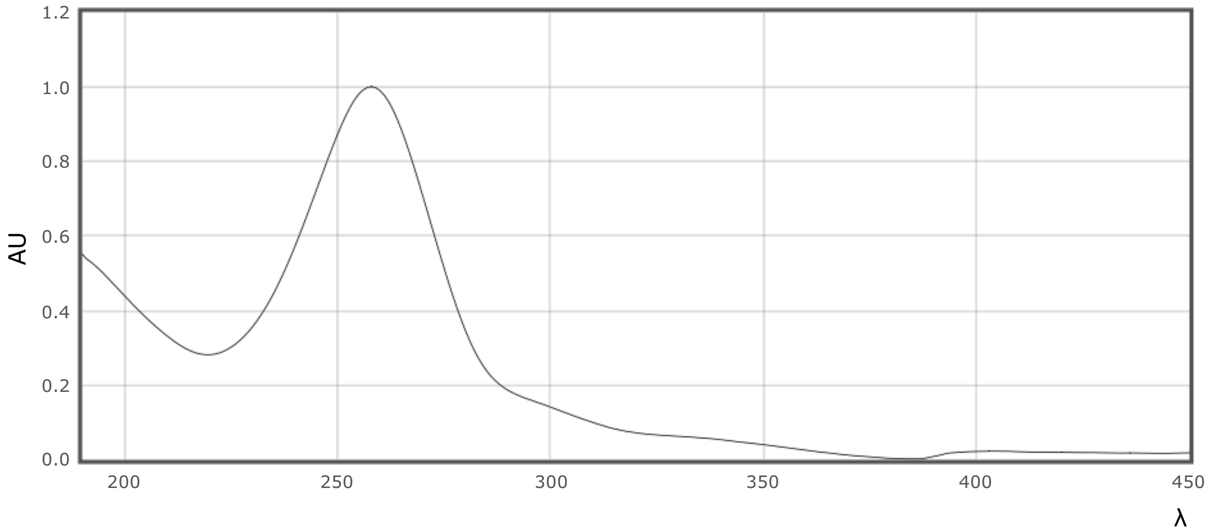

Tr. 12 Rf 0.508 (174.0 mm, 39.5 mm)

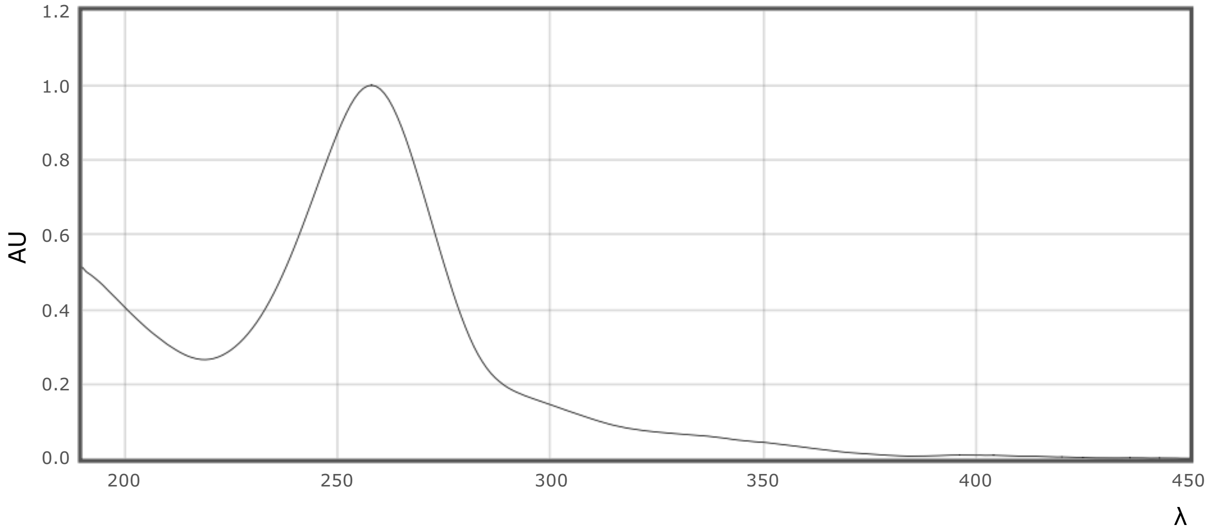

Spectrum correlation data:

Substance name	Track	Rf	r(s,m)	r(e,m)	Ref. spectrum	Correlation
Thymoquinone	1	0.529	0.000000	0.000000		0.000000
Thymoquinone	2	0.519	0.000000	0.000000		0.000000
Thymoquinone	3	0.518	0.000000	0.000000		0.000000
Thymoquinone	4	0.516	0.000000	0.000000		0.000000
Thymoquinone	5	0.509	0.000000	0.000000		0.000000
Thymoquinone	6	0.510	0.000000	0.000000		0.000000
Thymoquinone	7	0.514	0.000000	0.000000		0.000000
Thymoquinone	8	0.514	0.000000	0.000000		0.000000
Thymoquinone	9	0.508	0.000000	0.000000		0.000000
Thymoquinone	10	0.509	0.000000	0.000000		0.000000
Thymoquinone	11	0.509	0.000000	0.000000		0.000000
Thymoquinone	12	0.508	0.000000	0.000000		0.000000

Evaluation 1 :

Validated	true
Step	Scan developed plate 1b
Concentration unit type	Mass / volume
Notes	

101, A Wing, Aniket Apartment,
Navghar Road, Mulund (E),
Mumbai - 400 081, India.
Tel.: 091 22 21639928 - 31
E-mail : hptlc@anchrom.in
Web : www.anchrom.in

ANCHROM Enterprises (I) P. Ltd.
Technologists, not Traders!

Definition:

References:

RA1819285-01		
Substance Name	Concentration	Purity
Thymoquinone	1.000 mg/ml	100.00 %

Samples:

Vial ID	Amount	Volume solution	Reference amount	Related to
SA1819285-01	10.000 g	100.00 ml	0.000 mg	

Integration parameters:

Bounds	[0.404,0.608]
Smoothing	Savitzky–Golay of order 3 and window 7
Baseline correction	Lowest slope with noise 0.05
Profile subtraction	None
Peaks detection	Gauss (legacy) with sensitivity 0.1, separation 1 and threshold 0.1

Scan:

Wavelength	254 nm

Track 2:

Type	Reference
Vial ID	RA1819285-01
Description	Thymoquinone_1 mg/ml
Volume	2.0 µl

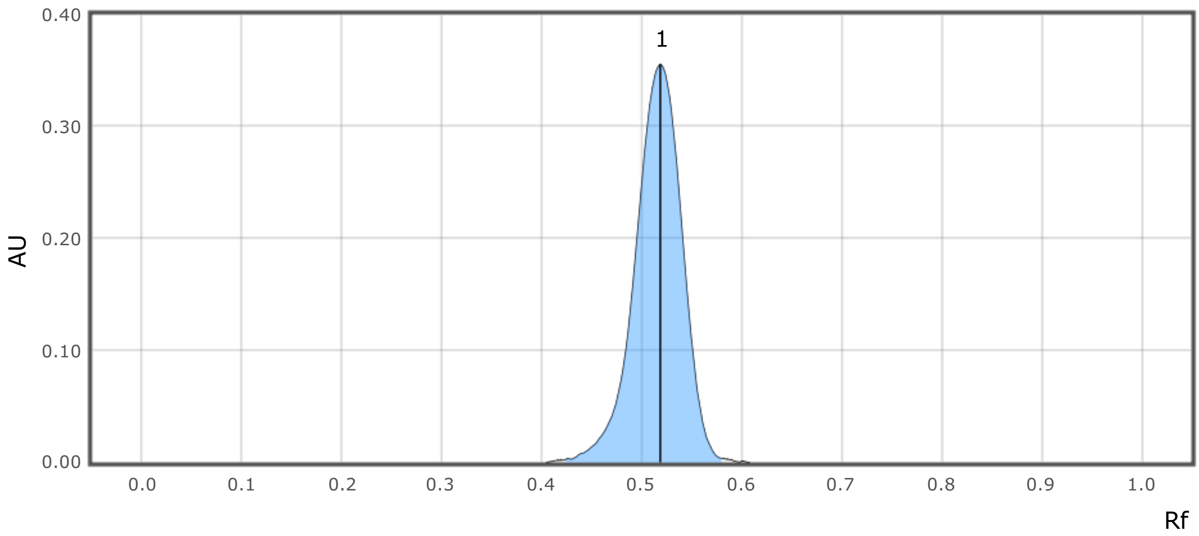

Peak #	Start		Max			End		Area		Manual peak	Substance Name
	Rf	H	Rf	H	%	Rf	H	A	%		
1	0.421	0.0019	0.519	0.3554	100.00	0.580	0.0036	0.01935	100.00	No	Thymoquinone

Track 3:

Type	Reference
Vial ID	RA1819285-01
Description	Thymoquinone_1 mg/ml
Volume	3.0 µl

101, A Wing, Aniket Apartment,
Navghar Road, Mulund (E),
Mumbai - 400 081, India.
Tel.: 091 22 21639928 - 31
E-mail : hptlc@anchrom.in
Web : www.anchrom.in

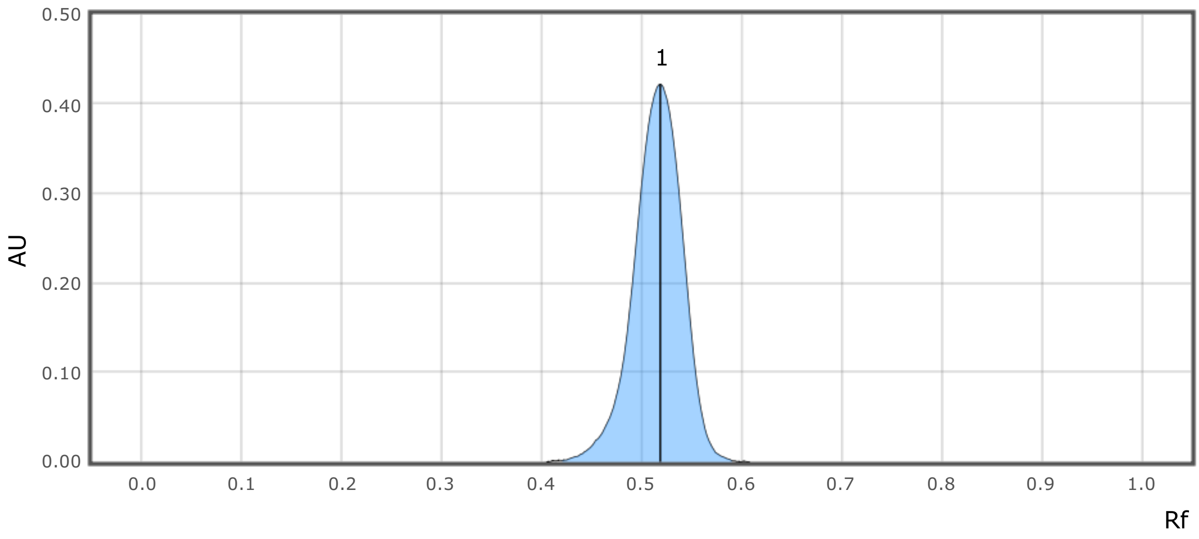

Peak #	Start		Max			End		Area		Manual peak	Substance Name
	Rf	H	Rf	H	%	Rf	H	A	%		
1	0.421	0.0015	0.519	0.4214	100.00	0.593	0.0010	0.02395	100.00	No	Thymoquinone

Track 4:

Type	Reference
Vial ID	RA1819285-01
Description	Thymoquinone_1 mg/ml
Volume	4.0 µl

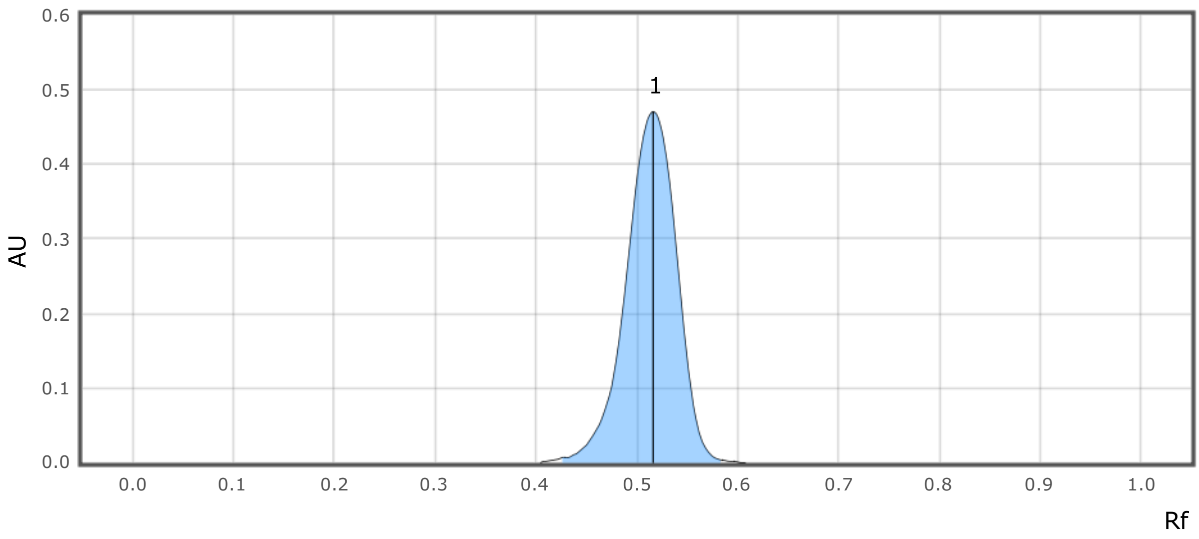

Peak #	Start		Max			End		Area		Manual peak	Substance Name
	Rf	H	Rf	H	%	Rf	H	A	%		
1	0.424	0.0058	0.516	0.4698	100.00	0.583	0.0037	0.02739	100.00	No	Thymoquinone

Track 5:

Type	Reference
Vial ID	RA1819285-01
Description	Thymoquinone_1 mg/ml
Volume	5.0 µl

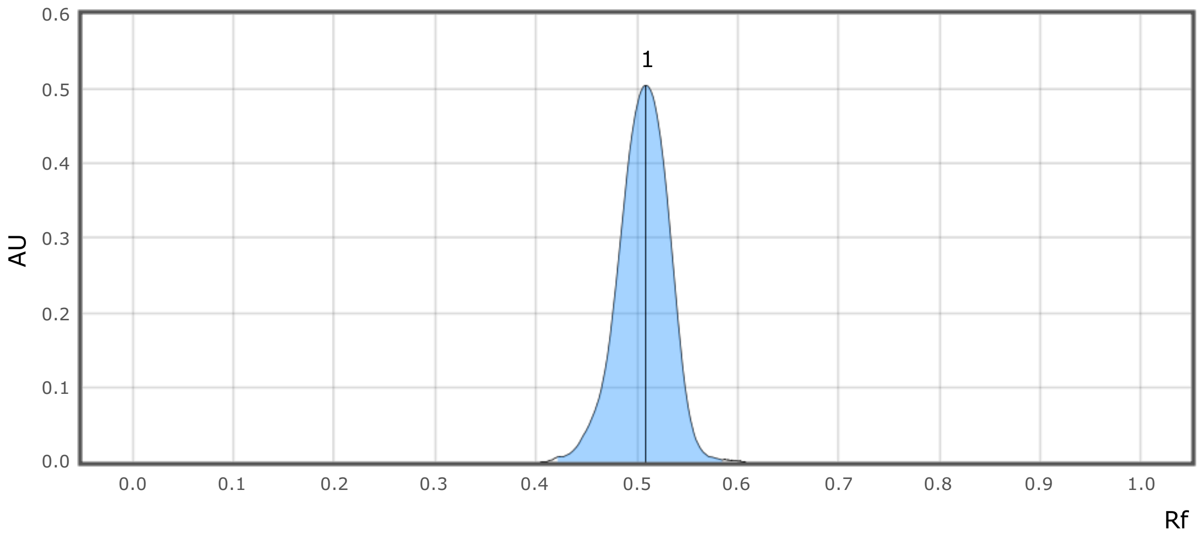

Peak #	Start		Max			End		Area		Manual peak	Substance Name
	Rf	H	Rf	H	%	Rf	H	A	%		
1	0.419	0.0047	0.509	0.5042	100.0 0	0.585	0.0024	0.03027	100.0 0	No	Thymoquinone

Track 6:	
Type	Reference
Vial ID	RA1819285-01
Description	Thymoquinone_1 mg/ml
Volume	6.0 µl

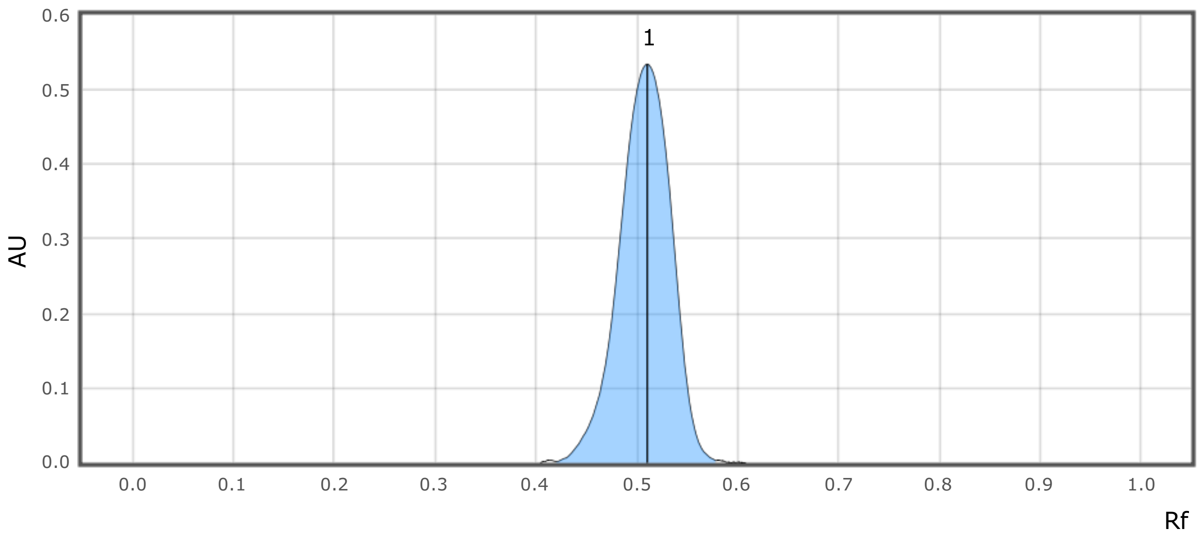

Peak #	Start		Max			End		Area		Manual peak	Substance Name
	Rf	H	Rf	H	%	Rf	H	A	%		
1	0.420	0.0014	0.510	0.5336	100.0 0	0.578	0.0029	0.03251	100.0 0	No	Thymoquinone

Track 10:	
Type	Sample
Vial ID	SA1819285-01
Description	Methanol extract of Nigella Sativa
Volume	20.0 µl

101, A Wing, Aniket Apartment,
Navghar Road, Mulund (E),
Mumbai - 400 081, India.
Tel.: 091 22 21639928 - 31
E-mail : hptlc@anchrom.in
Web : www.anchrom.in

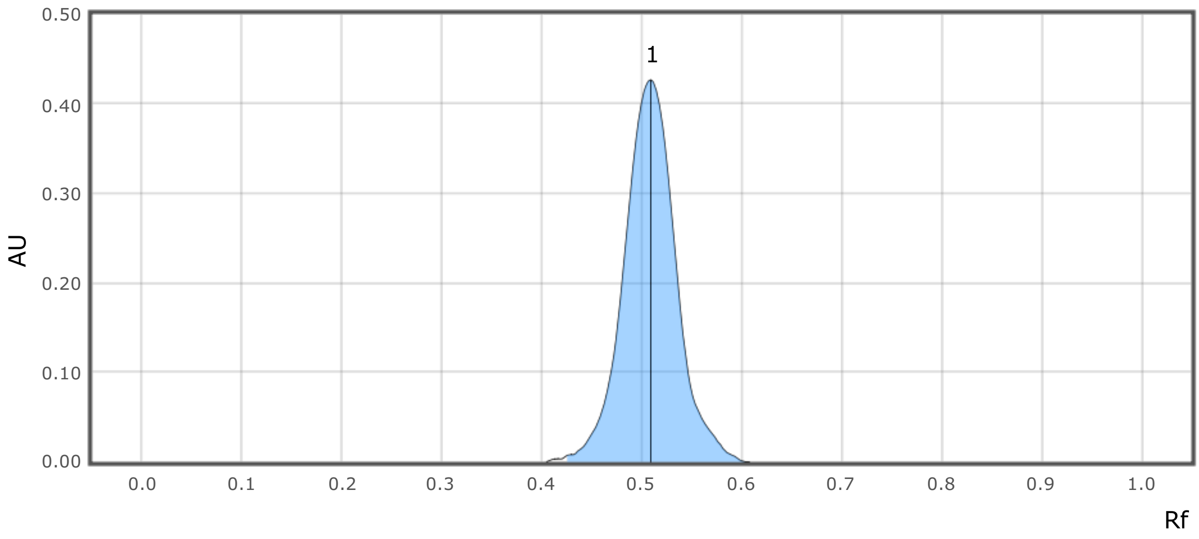

Peak #	Start		Max			End		Area		Manual peak	Substance Name
	Rf	H	Rf	H	%	Rf	H	A	%		
1	0.423	0.0053	0.509	0.4264	100.00	0.604	0.0000	0.02553	100.00	No	Thymoquinone

Track 11:	
Type	Sample
Vial ID	SA1819285-01
Description	Methanol extract of Nigella Sativa
Volume	20.0 µl

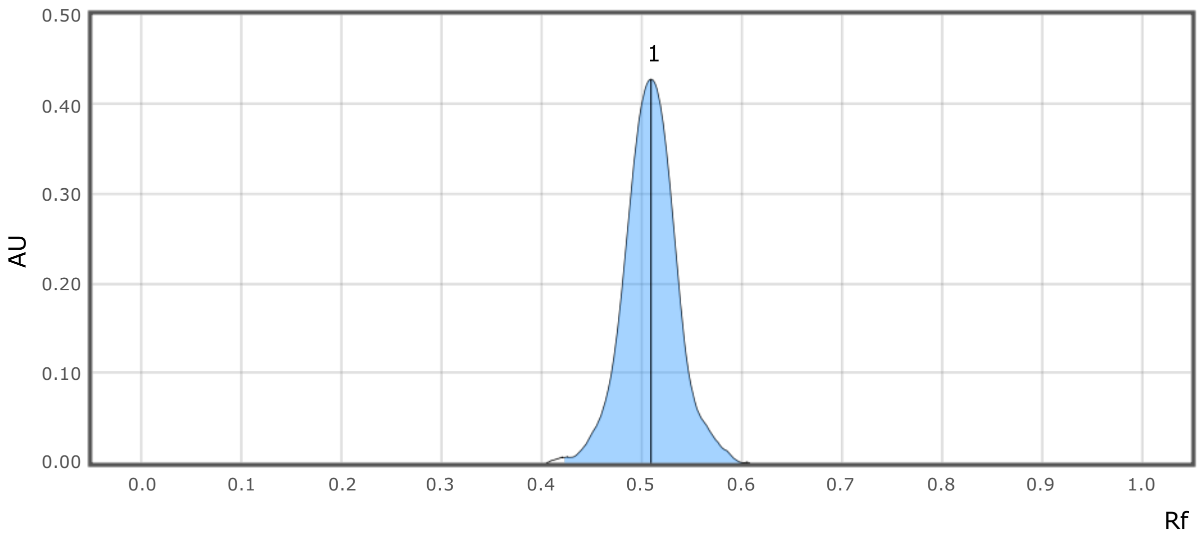

Peak #	Start		Max			End		Area		Manual peak	Substance Name
	Rf	H	Rf	H	%	Rf	H	A	%		
1	0.423	0.0059	0.509	0.4277	100.00	0.601	0.0000	0.02568	100.00	No	Thymoquinone

Calibration results:

Area calibration for substance Thymoquinone @ 254 nm:

101, A Wing, Aniket Apartment,
Navghar Road, Mulund (E),
Mumbai - 400 081, India.
Tel.: 091 22 21639928 - 31
E-mail : hptlc@anchrom.in
Web : www.anchrom.in

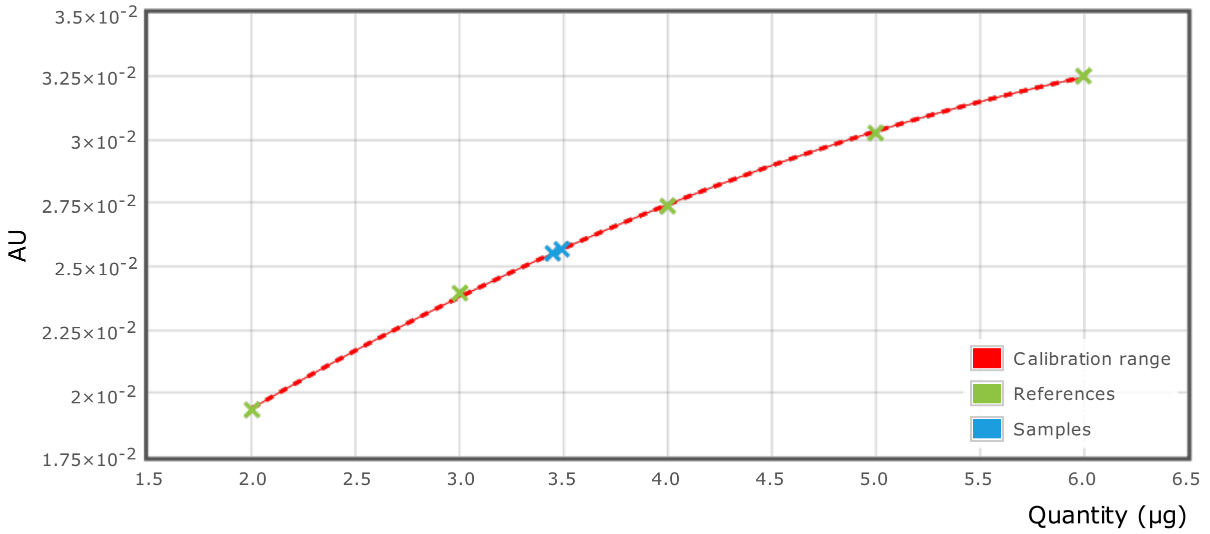

Regression mode	Polynomial
Range deviation	0.00 %
Related substances	Default
Number of references	5
Calibration function	$y=-3.761\times10^{-16}x^{2}+6.272\times10^{-9}x+8.375\times10^{-3}$
Coefficient of variation	CV 0.31 %
Correlation coefficient	R=99.983713 %

Results:

Thymoquinone	(2 sample assignments) @ 254 nm		
Sample 'SA1819285-01'	173.4 µg/ml	CV=0.88 %	(2 applications)
			17.34 mg in 10.000 g
Volume: 20.0 µl	173.4 µg/ml	CV=0.88 %	(2 replicas)
Track 10	172.3 µg/ml	3.446 µg	
Track 11	174.5 µg/ml	3.489 µg	

A track marked with ⚠ means: this result is outside the regression range given by the reference assignments, but is included in the results because it is in the allowed range deviation.

Remarks

Conclusion: The samples shows 17.34 mg of Thymoquinone in 10 gm. The percentage of Thymoquinone detected was 0.17%

101, A Wing, Aniket Apartment,
Navghar Road, Mulund (E),
Mumbai - 400 081, India.
Tel.: 091 22 21639928 - 31
E-mail : hptlc@anchrom.in
Web : www.anchrom.in

E-signed

Level: 1 - Lab Analyst

Signed by	Lab_Sneha
Date	05-Mar-2019 11:32:18 UTC
Comment	final result

Level: 2 - Asst. Lab Manager

Signed by	Lab_saikat
Date	05-Mar-2019 11:34:34 UTC
Comment	OK
