# Supplementary material for: Standardization of Berberis aristata DC and Nigella sativa L. Using HPTLC and GCMS and Their Antineoplasia Activity in 7,12-Dimethylbenz[a]anthracene-Induced Mouse Models
Source: Front Pharmacol. 2021 Nov 30;12:642067. doi: 10.3389/fphar.2021.642067 (PMC8670326; doi:10.3389/fphar.2021.642067)
Supplement: Supplementary file 2 [file DataSheet3.PDF]

Sample Information

Analyzed by : Admin
Analyzed : 8/8/2019 8:06:10 PM
Sample Name : Nigella sativa oil
Data File : D:\GCMS DATA\GC-MS Data\DIPSAR\8.8.2019\Nigella sativa oil-R.qgd

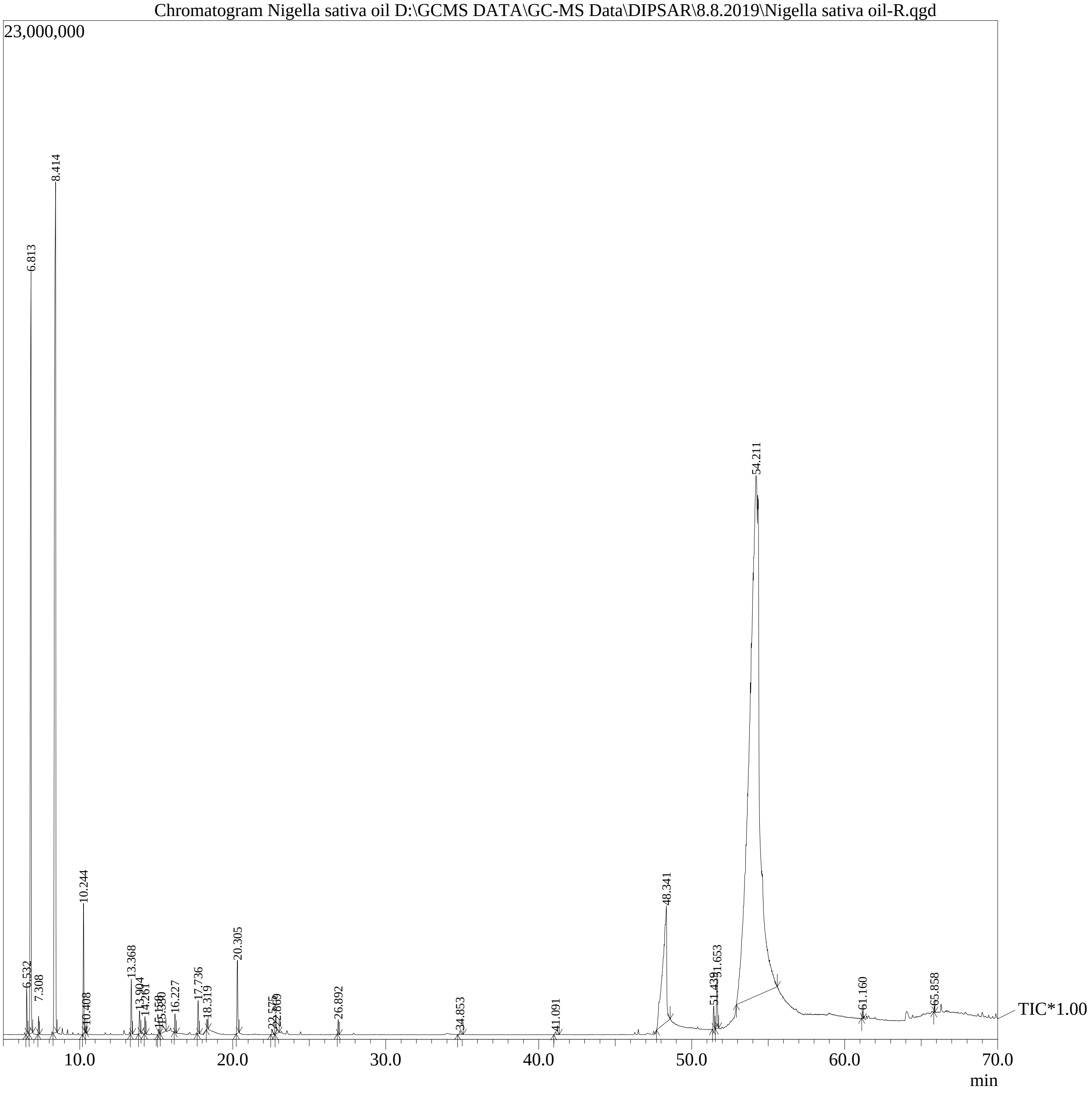


Peak Report TIC

| Peak# | R.Time | Area | Area% | Name |
|---|---|---|---|---|
| 1 | 6.532 | 3008277 | 0.36 | Thujene <alpha-> |
| 2 | 6.813 | 75532375 | 8.93 | BICYCLO[3.1.1]HEPT-2-ENE, 2,6,6-TRIMETHYL- |
| 3 | 7.308 | 1063045 | 0.13 | Camphene |
| 4 | 8.414 | 115567825 | 13.66 | Pinene oxide <beta-> |
| 5 | 10.244 | 9042970 | 1.07 | Cymene <para-> |
| 6 | 10.408 | 342467 | 0.04 | CYCLOHEXENE, 1-METHYL-4-(1-METHYLETHENYL)-, (S)- |

| Peak# | R.Time | Area | Area% | Name |
|---|---|---|---|---|
| 7 | 13.368 | 4033463 | 0.48 | 3-Oxatricyclo[4.1.1.0(2,4)]octane, 2,7,7-trimethyl- |
| 8 | 13.904 | 2122686 | 0.25 | Pinene oxide <alpha-> |
| 9 | 14.261 | 1263376 | 0.15 | (1R,4R,5S)-1-Isopropyl-4-methoxy-4-methylbicyclo[3.1.0]hexane |
| 10 | 15.158 | 365669 | 0.04 | Nopinone |
| 11 | 15.330 | 2293068 | 0.27 | |
| 12 | 16.227 | 1444460 | 0.17 | Pinocarvone |
| 13 | 17.736 | 2646046 | 0.31 | Myrtenal |
| 14 | 18.319 | 835255 | 0.10 | |
| 15 | 20.305 | 6423268 | 0.76 | Thymoquinone |
| 16 | 22.575 | 568936 | 0.07 | THUJYL ACETATE <NEO-ISO-3-> DB5-1081 (CLASSICAL NAME = NEO-3-) |
| 17 | 22.869 | 876235 | 0.10 | PINOCARVEOL <TRANS-> DB5-724 |
| 18 | 26.892 | 1176740 | 0.14 | (+)-LONGIFOLEN |
| 19 | 34.853 | 462953 | 0.05 | |
| 20 | 41.091 | 397933 | 0.05 | Tetradecanoic acid <n-> |
| 21 | 48.341 | 48096480 | 5.69 | Hexadecanoic acid <n-> |
| 22 | 51.439 | 2139770 | 0.25 | 9,12-OCTADECADIENOIC ACID (Z,Z)-, METHYL ESTER |
| 23 | 51.653 | 5090837 | 0.60 | 9-Undecenal, 2,10-dimethyl- |
| 24 | 54.211 | 560235582 | 66.24 | Linoleic acid |
| 25 | 61.160 | 478053 | 0.06 | 3-Cyclopentylpropionic acid, 2-dimethylaminoethyl ester |
| 26 | 65.858 | 278309 | 0.03 | Squalene |
| | | 845786078 | 100.00 | |
