# Supplementary material for: Standardization of Berberis aristata DC and Nigella sativa L. Using HPTLC and GCMS and Their Antineoplasia Activity in 7,12-Dimethylbenz[a]anthracene-Induced Mouse Models
Source: Front Pharmacol. 2021 Nov 30;12:642067. doi: 10.3389/fphar.2021.642067 (PMC8670326; doi:10.3389/fphar.2021.642067)
Supplement: Supplementary file 3 [file DataSheet1.PDF]

101, A Wing, Aniket Apartment,
Navghar Road, Mulund (E),
Mumbai - 400 081, India.
Tel.: 091 22 21639928 - 31
E-mail : hptlc@anchrom.in
Web : www.anchrom.in

Analysis: 1819284_20190302_(P3)

Path: Home/1819284

Based on method: 1819284

Created	04-Mar-2019 09:52:01	Lab_Sneha
Modified	05-Mar-2019 17:03:30	Saikat Mallick
Last HPTLC log	05-Mar-2019 17:05:07	File has been e-signed
Explorer notes		

Track	Vial ID	Description	Volume	Position	Type
1	RA1819284-01	Berberine Chloride(0.1 mg/ml)	1.0 µl	B1	Reference
2	RA1819284-01	Berberine Chloride(0.1 mg/ml)	2.0 µl	B1	Reference
3	RA1819284-01	Berberine Chloride(0.1 mg/ml)	3.0 µl	B1	Reference
4	RA1819284-01	Berberine Chloride(0.1 mg/ml)	4.0 µl	B1	Reference
5	RA1819284-01	Berberine Chloride(0.1 mg/ml)	5.0 µl	B1	Reference
6	RA1819284-01	Berberine Chloride(0.1 mg/ml)	6.0 µl	B1	Reference
7	RA1819284-01	Berberine Chloride(0.1 mg/ml)	7.0 µl	B1	Reference
8	SA1819284-01	Berberis aristata(10 mg/ml)_1:100	2.0 µl	B2	Sample
9	SA1819284-01	Berberis aristata(10 mg/ml)_1:100	2.0 µl	B2	Sample
10	SA1819284-01	Berberis aristata(10 mg/ml)_1:100	5.0 µl	B2	Sample
11	SA1819284-01	Berberis aristata(10 mg/ml)_1:100	5.0 µl	B2	Sample
12	SA1819284-01	Berberis aristata(10 mg/ml)_1:100	5.0 µl	B2	Sample
Sequence table notes					

A track marked with ⚠ means: the application type is overridden in some evaluation(s).

System setup:

Software	Server VisionCATS-Server-PH, version 2.5.18262.1
ATS4	S/N:250243 (NEW ATS4)
Chamber	N/A
Scanner4	S/N:170422 (SCANNER 4)
Visualizer	S/N:150503 (VISUALIZER)

Chromatography

Plate layout:

Stationary phase	Merck, TLC plates Al silica gel 60 F 254
Plate format	200.0 x 100.0 mm
Application type	BandIntertrack
Application	Position Y: 8.0 mm, length: 8.0 mm, width: 0.0 mm
Track	First position X: 20.0 mm, distance: 14.0 mm
Solvent front position	70.0 mm
Notes	

Take image clean plate 1a - Visualizer (S/N: 150503):

Quality	Enhanced
R White	auto capture, Auto, level 85 %, Band
R 254	auto capture, Auto, level 85 %, Band
R 366	auto capture, Auto, level 85 %, Band
Instrument diagnostics	Valid diagnostics
Documentation step label	
Notes	

Application 1 - ATS 4 (S/N: 250243):

101, A Wing, Aniket Apartment,
Navghar Road, Mulund (E),
Mumbai - 400 081, India.
Tel.: 091 22 21639928 - 31
E-mail : hptlc@anchrom.in
Web : www.anchrom.in

Spray gas	Air
Sample solvent type	Methanol
Filling speed	15 µl/s
Predosage volume	200 nl
Retraction volume	200 nl
Dosage speed	150 nl/s
Filling quality	Standard
Rinsing cycles / vacuum	1 / 4 s
Filling cycles / vacuum	1 / 4 s
Rinsing solvent name	Methanol
Nozzle temperature	Unheated
Rack in use	
Instrument diagnostics	Valid diagnostics
Notes	

Development 1 - Chamber:

Tank	TTC 20x10
Mobile phase	n butanol:acetic acid:water(3:1:1)v/v/v
Saturation time	20 min
Use saturation pad	true
Use smartALERT	false
Volume front through	10 ml
Volume rear through	10 ml
Drying time	5 min
Drying temperature	Room temperature
Notes	

Take image developed plate 1a - Visualizer (S/N: 150503):

Quality	Enhanced
R White	auto capture, Auto, level 85 %, Band
R 366	auto capture, Auto, level 85 %, Band
Instrument diagnostics	Valid diagnostics
Documentation step label	
Notes	

Take image developed plate 1b - Visualizer (S/N: 150503):

Quality	Enhanced
R 254	auto capture, Auto, level 85 %, Band
Instrument diagnostics	Valid diagnostics
Documentation step label	
Notes	

Scan developed plate 1c - Scanner 4 (S/N: 170422):

Scanner type	Single λ
Optimization for	Resolution
Measurement mode	Absorption
Filter	n/a
Detector mode	Automatic
Scanning speed	20 mm/s
Data resolution	100 µm/step
Slit	5 x 0.2 mm, micro
Partial scan	No
Lamp	Deuterium
Wavelength(s)	254 nm
Instrument diagnostics	Valid diagnostics
Documentation step label	
Notes	

101, A Wing, Aniket Apartment,
Navghar Road, Mulund (E),
Mumbai - 400 081, India.
Tel.: 091 22 21639928 - 31
E-mail : hptlc@anchrom.in
Web : www.anchrom.in

Scan developed plate 1d - Scanner 4 (S/N: 170422):

Scanner type	Single λ
Optimization for	Light (sensitivity)
Measurement mode	Absorption
Filter	n/a
Detector mode	Automatic
Scanning speed	20 mm/s
Data resolution	25 µm/step
Slit	6 x 0.45 mm, micro
Partial scan	No
Lamp	Mercury
Wavelength(s)	366 nm
Instrument diagnostics	Valid diagnostics
Documentation step label	
Notes	

Spectrum Scan developed plate 1e - Scanner 4 (S/N: 170422):

Scanner type	Spectrum
Optimization for	Light (sensitivity)
Measurement mode	Absorption
Filter	n/a
Detector mode	Automatic
Spectrum speed	20 nm/s
Data resolution	1 nm
Slit	6 x 0.45 mm, micro
Lamp	Deuterium
Wavelength range	190 nm to 450 nm
Reference spectrum	Per plate, X=10.0 mm, Y=10.0 mm
Purity	No
Instrument diagnostics	Valid diagnostics
Documentation step label	
Notes	

Substance berberine HCl (Rf. 0.488 +/- 0.033):

Track	Rf	X (mm)	Y (mm)
1	0.485	20.0	38.1
2	0.481	34.0	37.8
3	0.482	48.0	37.9
4	0.481	62.0	37.8
5	0.481	76.0	37.8
6	0.489	90.0	38.3
7	0.492	104.0	38.5
8	0.489	118.0	38.3
9	0.490	132.0	38.4
10	0.502	146.0	39.1
11	0.505	160.0	39.3
12	0.506	174.0	39.4

System suitability tests:

SST settings:

SST tracks	

Data acquisition

Application 1 - ATS 4 (S/N: 250243):

Executed	04-Mar-2019 10:37:10 Lab_Sneha

Development 1 - Chamber:

Executed	04-Mar-2019 11:42:10 Lab_Sneha

Take image developed plate 1a - Visualizer (S/N: 150503):

Executed	04-Mar-2019 11:47:21 Lab_Sneha

R White Developed, RemissionVis

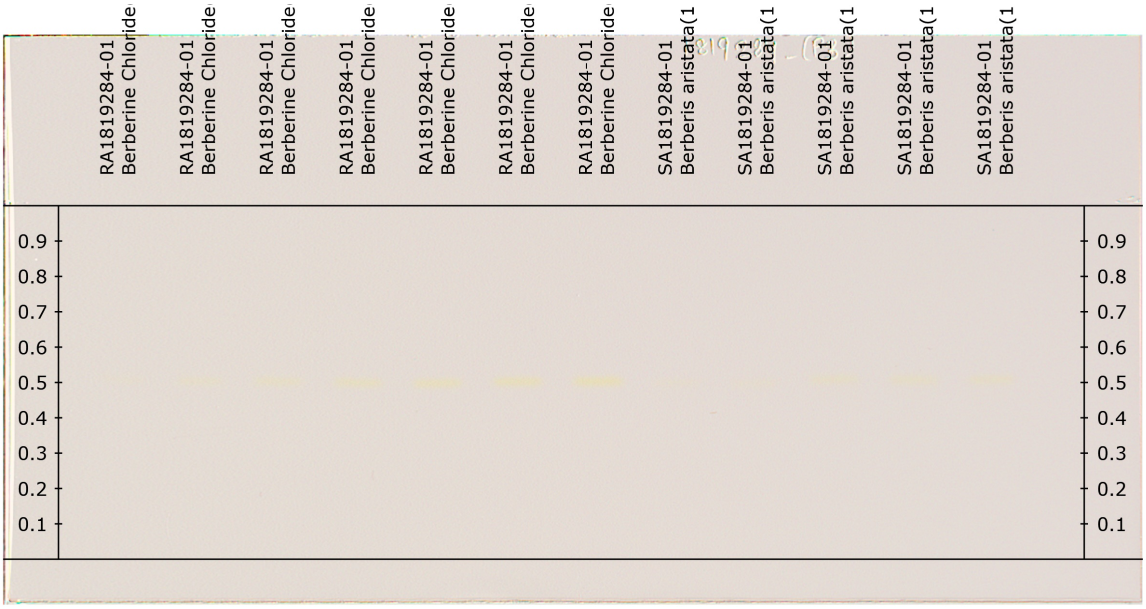

Exposure	0.065 s
Contrast	1
Normalized exposure	Disabled
Clarify	Disabled
White balance	1.00, 1.00, 1.00

101, A Wing, Aniket Apartment,
Navghar Road, Mulund (E),
Mumbai - 400 081, India.
Tel.: 091 22 21639928 - 31
E-mail : hptlc@anchrom.in
Web : www.anchrom.in

R 366 Developed, Remission366

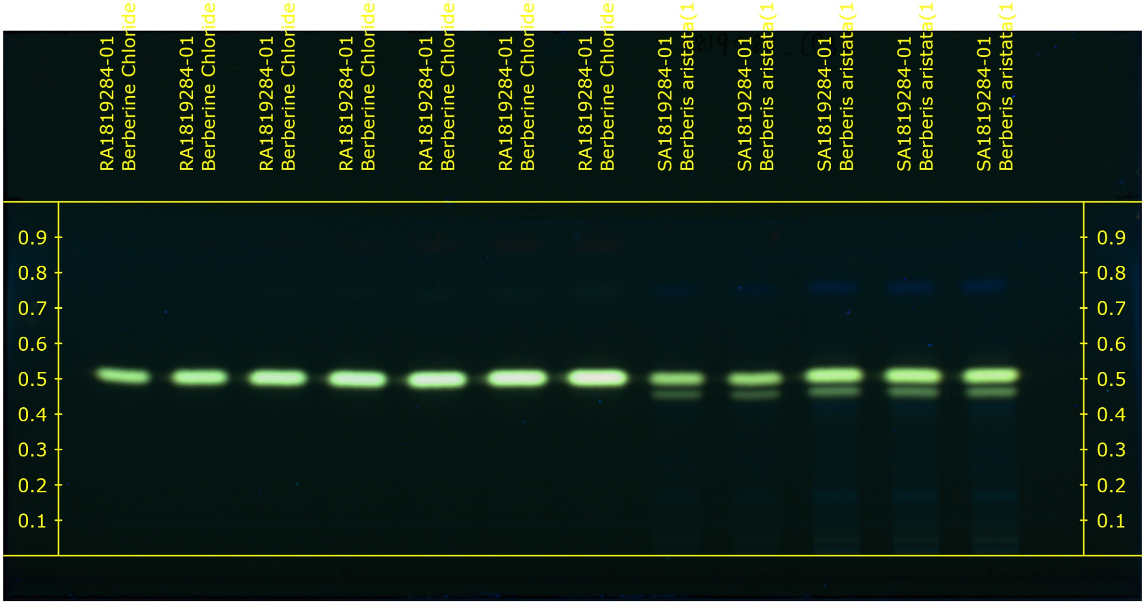

Exposure	0.273 s
Contrast	1
Normalized exposure	Enabled
Clarify	Disabled
White balance	1.00, 1.00, 1.00

Take image developed plate 1b - Visualizer (S/N: 150503):

Executed	04-Mar-2019 12:07:49 Lab_Sneha

R 254 Developed, Remission254

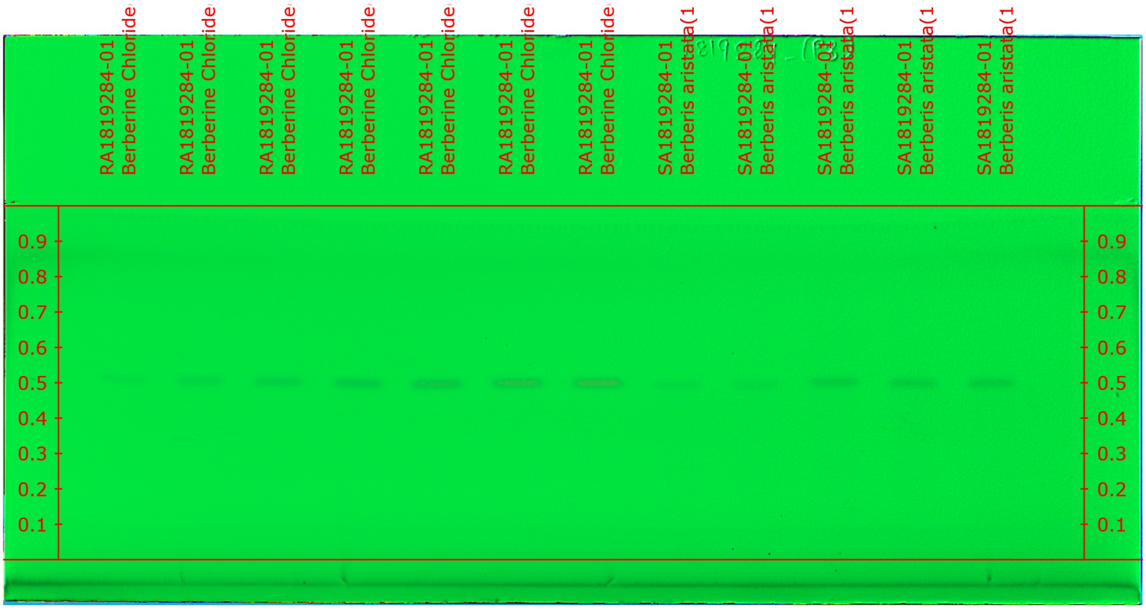

Exposure	0.340 s
Contrast	1
Normalized exposure	Disabled
Clarify	Disabled
White balance	1.00, 1.00, 1.00

Scan developed plate 1c - Scanner 4 (S/N: 170422):

Executed	04-Mar-2019 12:10:04 Lab_Sneha

101, A Wing, Aniket Apartment,
Navghar Road, Mulund (E),
Mumbai - 400 081, India.
Tel.: 091 22 21639928 - 31
E-mail : hptlc@anchrom.in
Web : www.anchrom.in

Scan:	
Wavelength	254 nm

Track 1:	
Type	Single λ

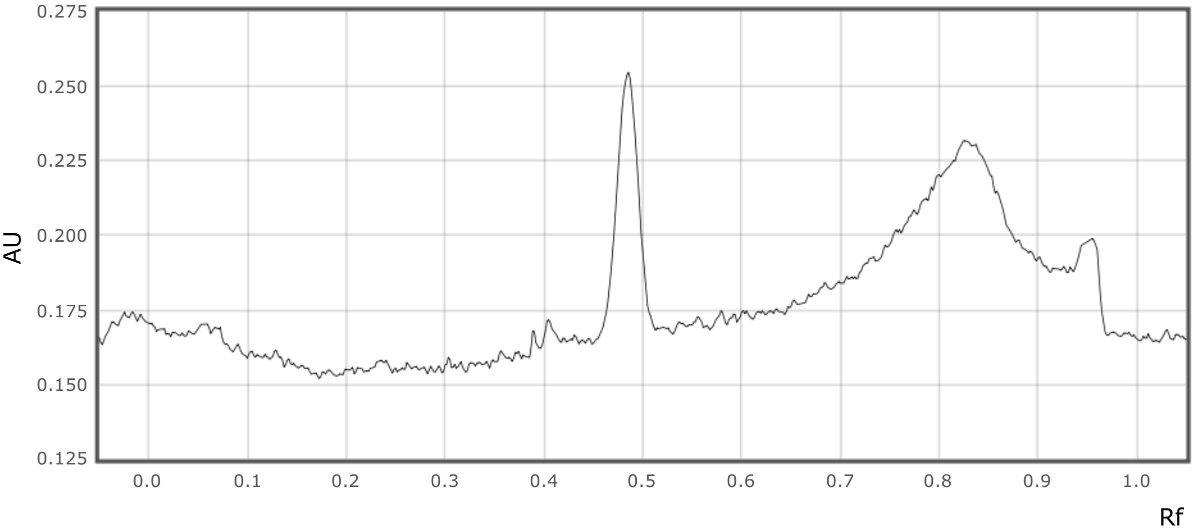

Track 2:	
Type	Single λ

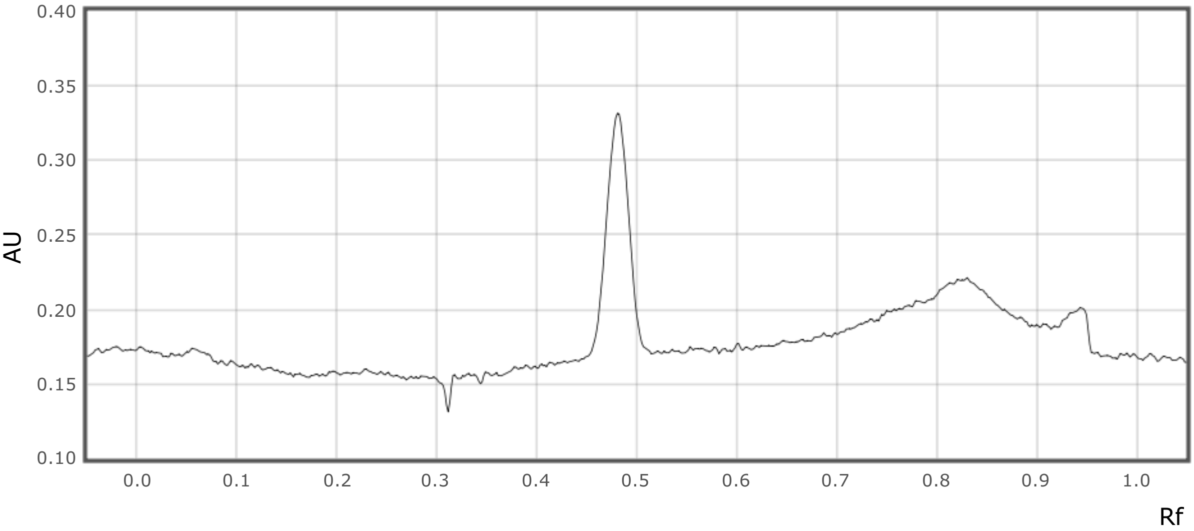

Track 3:	
Type	Single λ

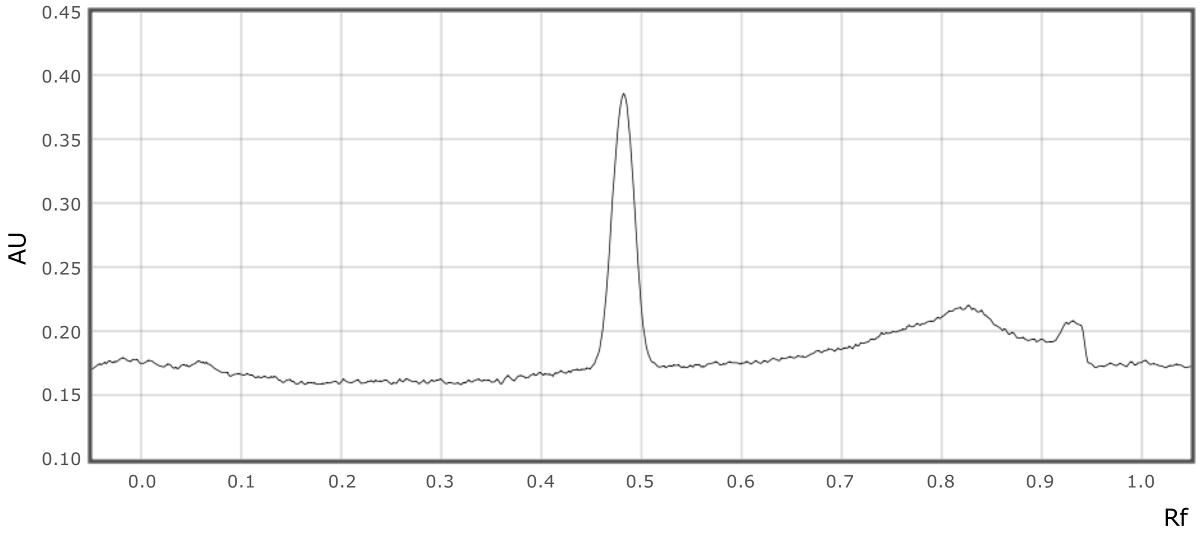

Track 4:

Type	Single λ

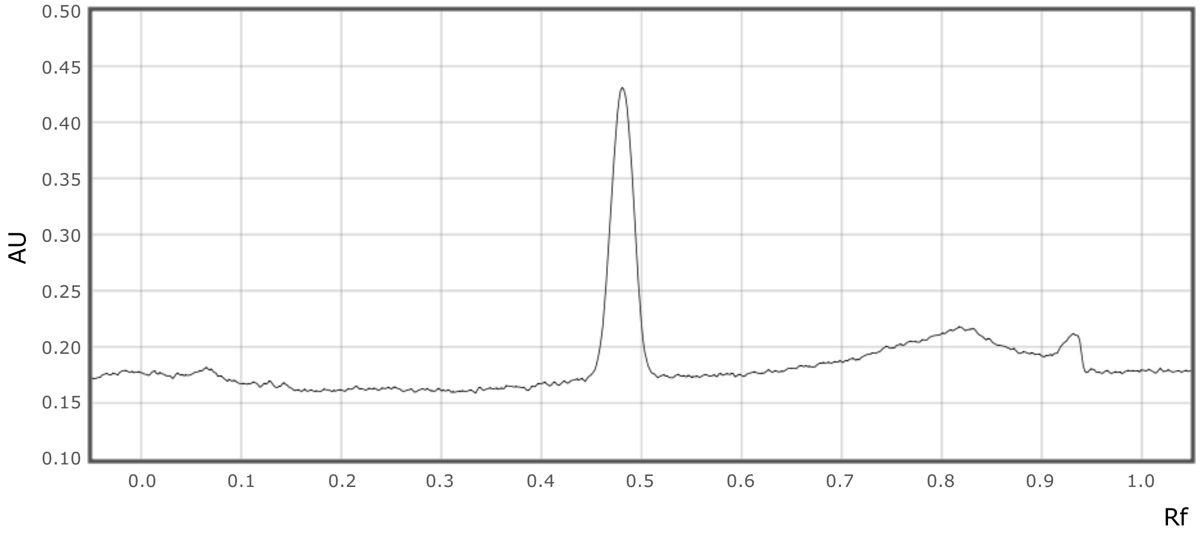

Track 5:

Type	Single λ

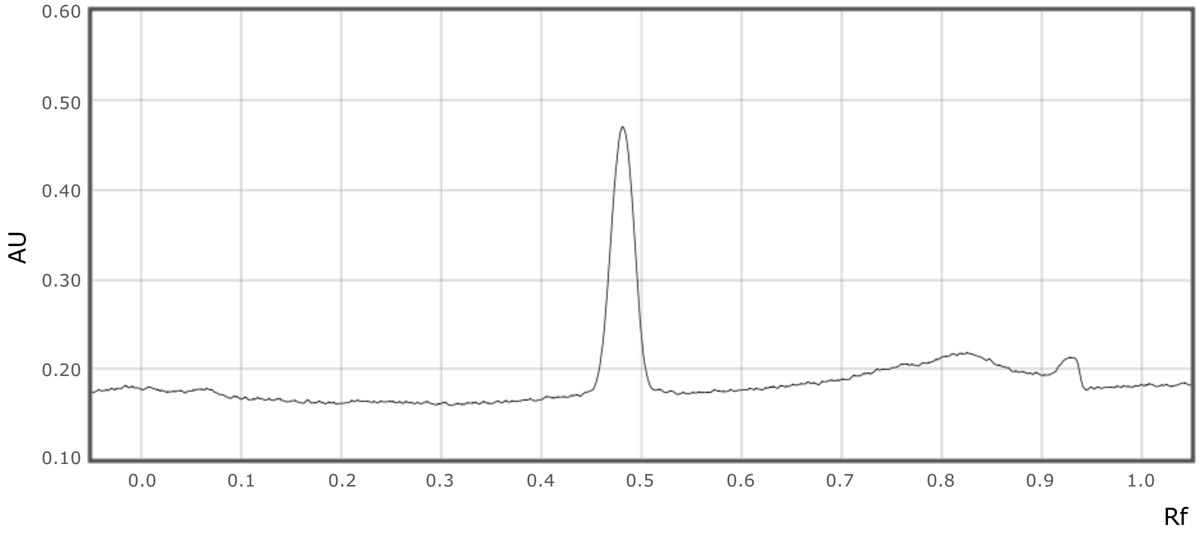

Track 6:

Type	Single λ

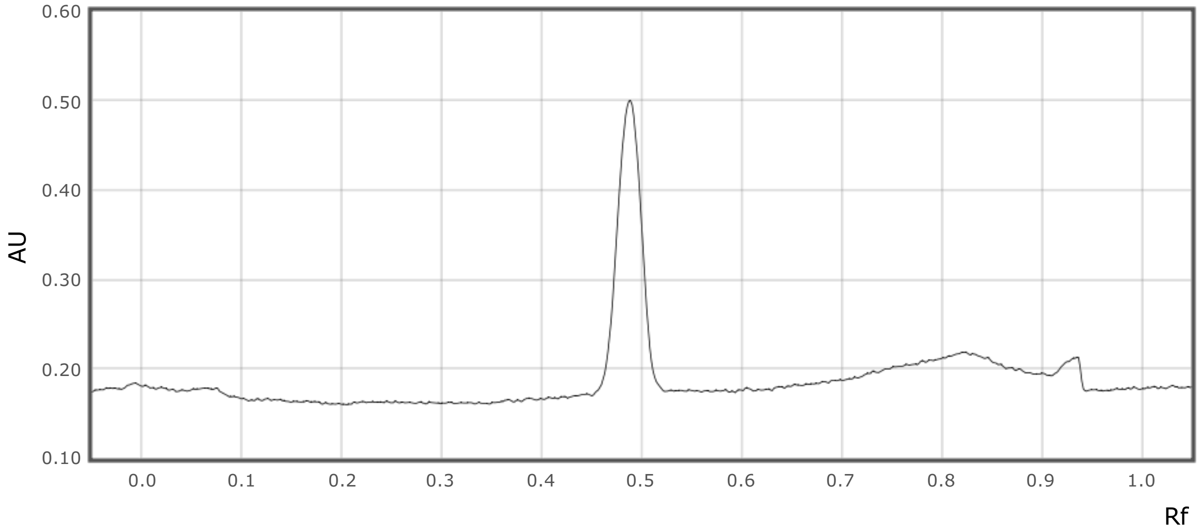

Track 7:

Type	Single λ

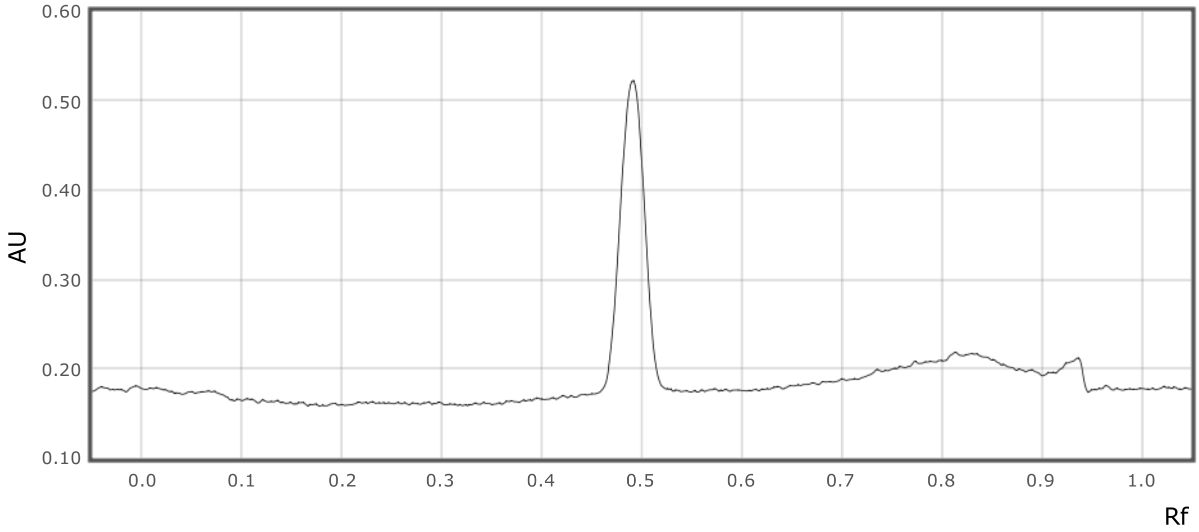

Track 8:

Type	Single λ

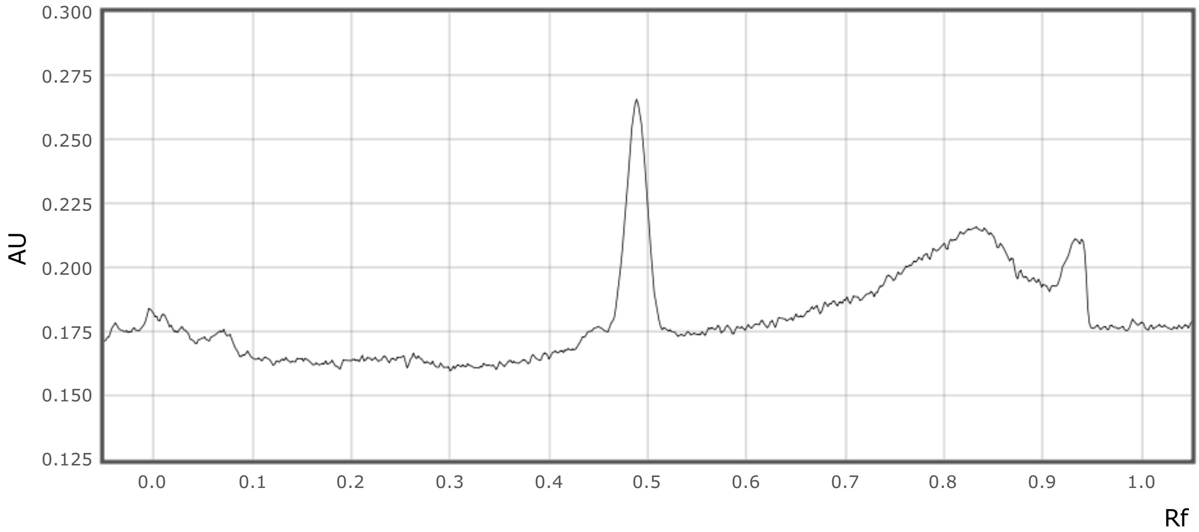

Track 9:

Type	Single λ

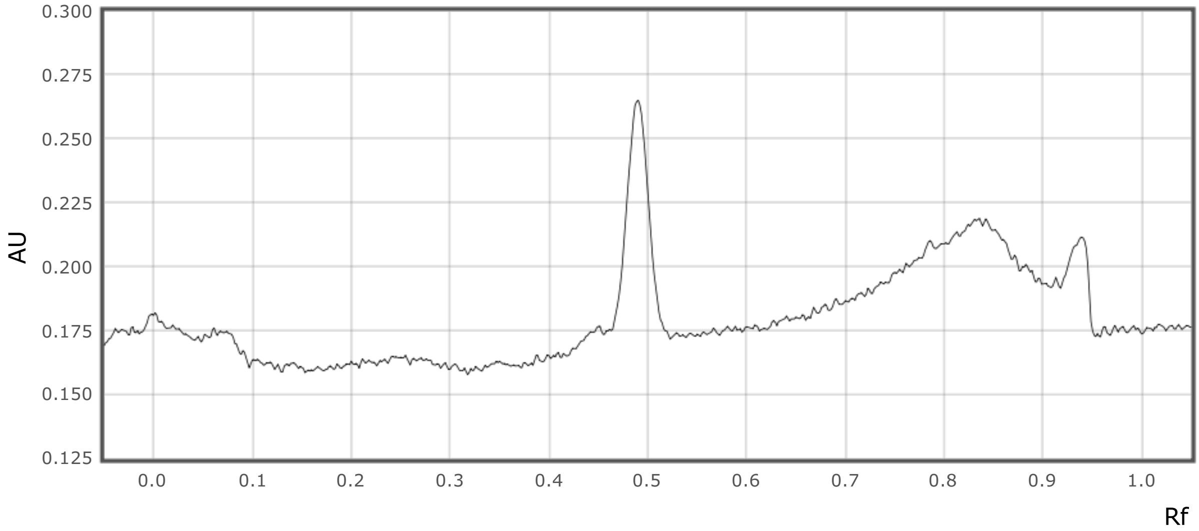

Track 10:

Type	Single λ

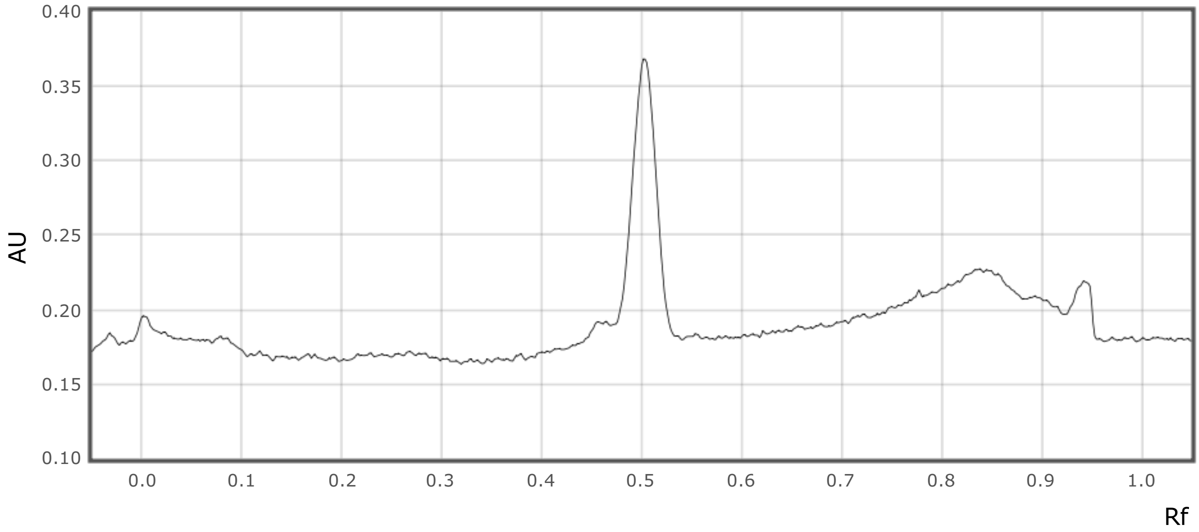

101, A Wing, Aniket Apartment,
Navghar Road, Mulund (E),
Mumbai - 400 081, India.
Tel.: 091 22 21639928 - 31
E-mail : hptlc@anchrom.in
Web : www.anchrom.in

Track 11:

Type	Single λ

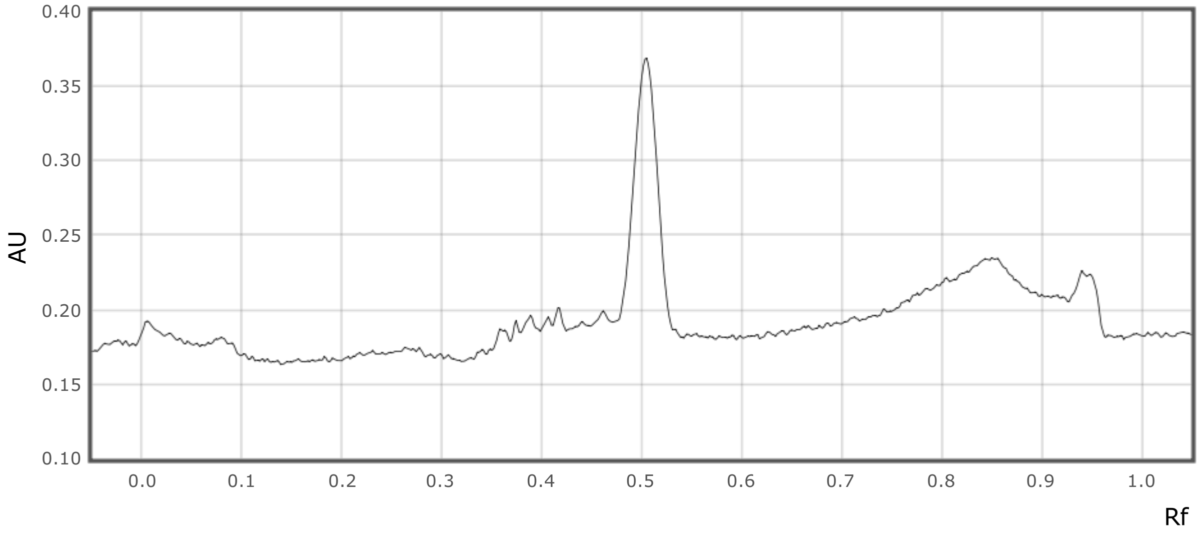

Track 12:

Type	Single λ

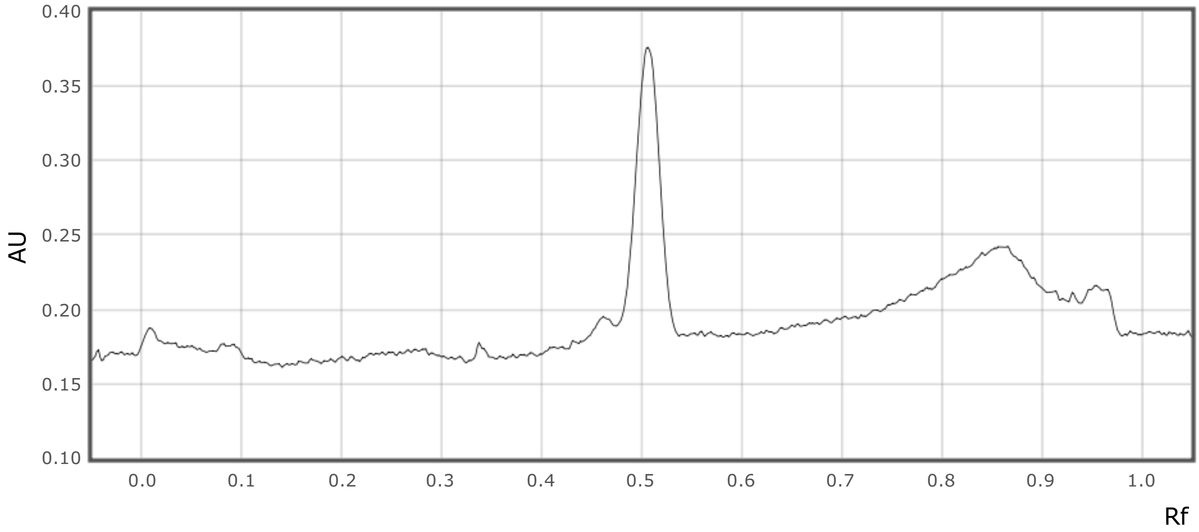

Scan developed plate 1d - Scanner 4 (S/N: 170422):

Executed	04-Mar-2019 12:12:14 Lab_Sneha

Scan:

Wavelength	366 nm

Track 1:

Type	Single λ

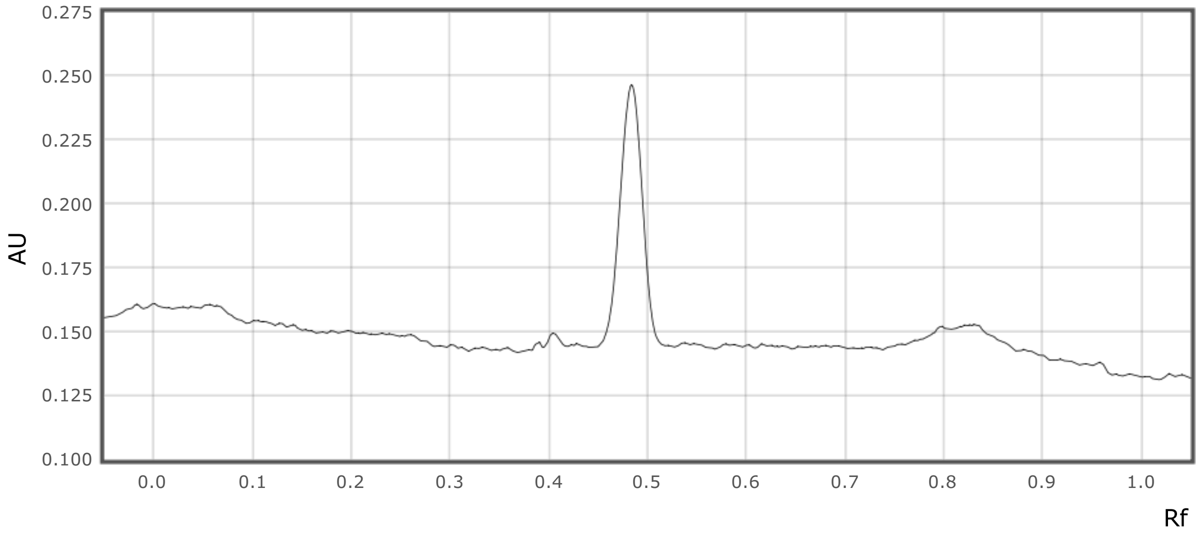

Track 2:

Type	Single λ

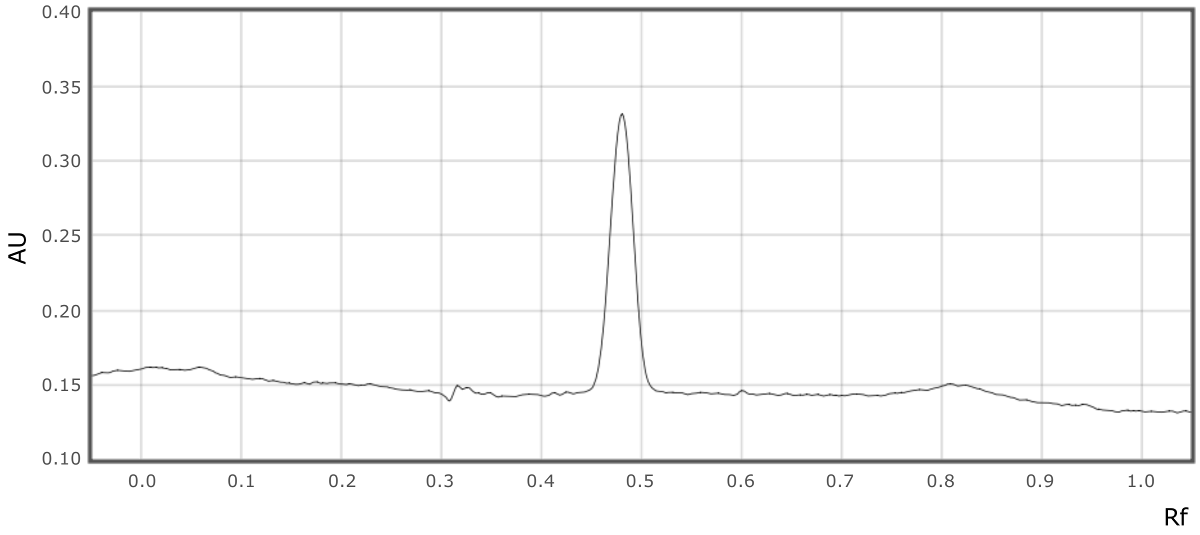

Track 3:

Type	Single λ

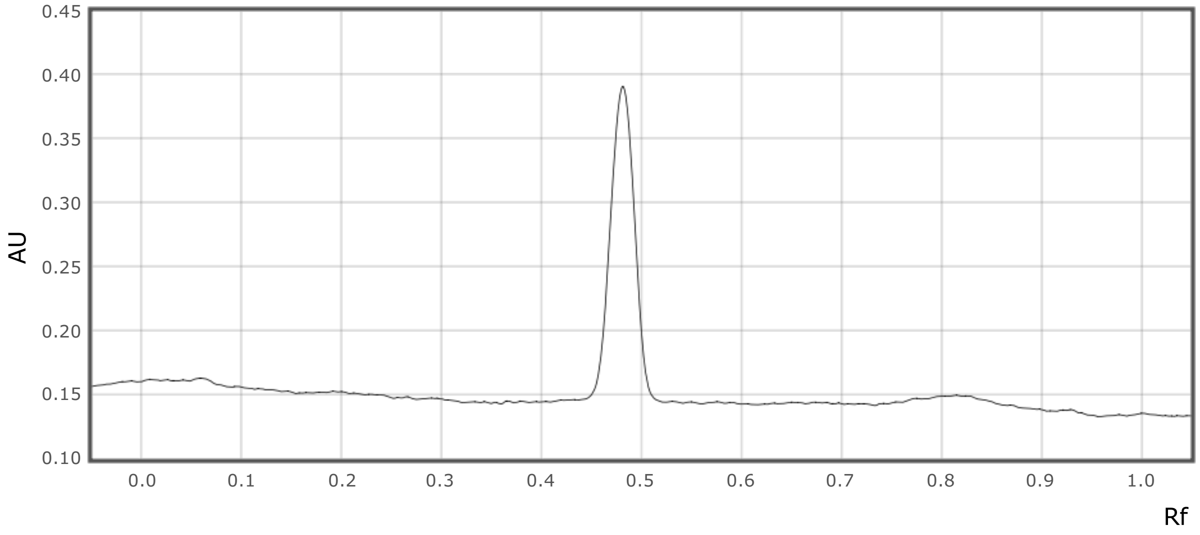

101, A Wing, Aniket Apartment,
Navghar Road, Mulund (E),
Mumbai - 400 081, India.
Tel.: 091 22 21639928 - 31
E-mail : hptlc@anchrom.in
Web : www.anchrom.in

Track 4:

Type	Single λ

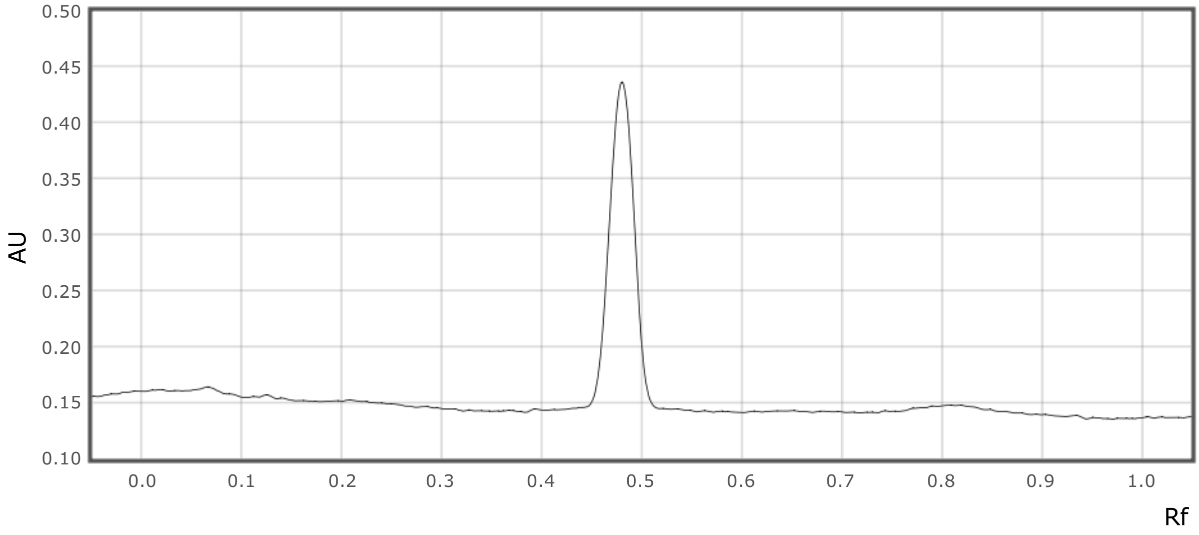

Track 5:

Type	Single λ

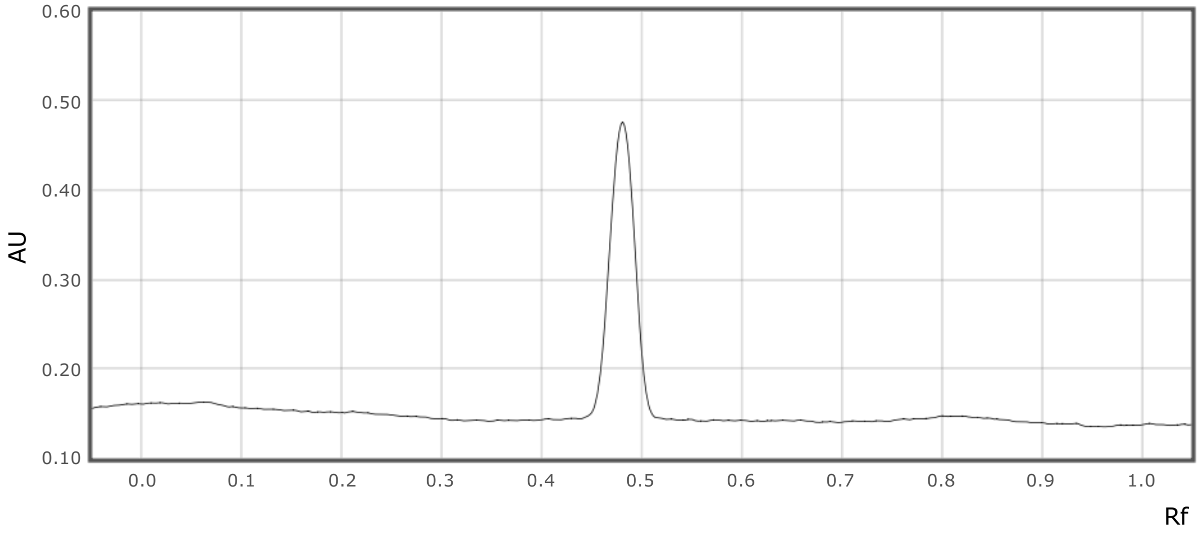

Track 6:

Type	Single λ

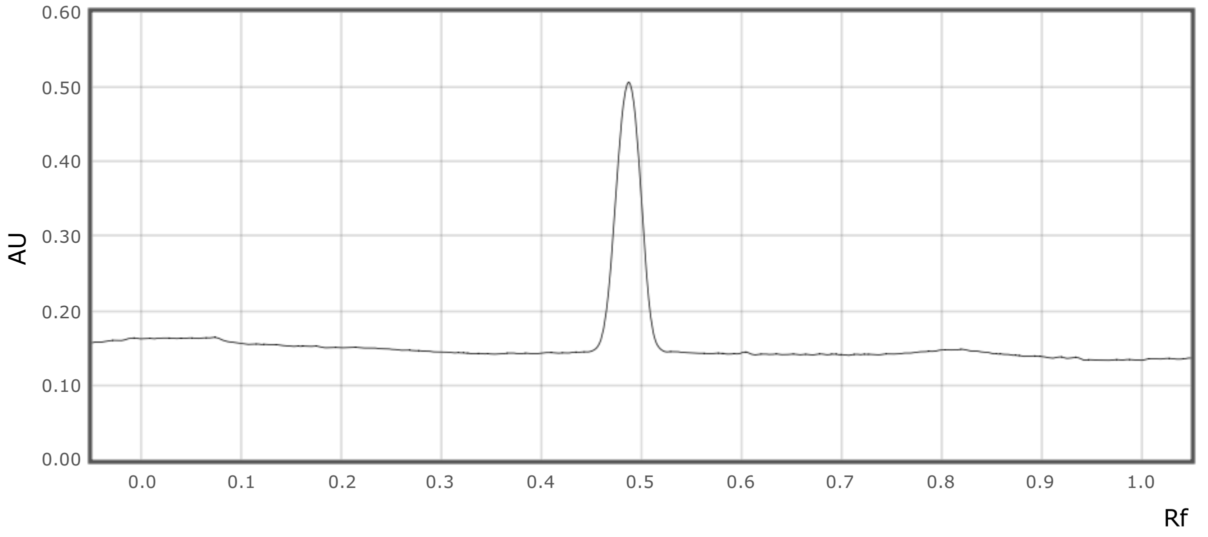

Track 7:

Type	Single λ

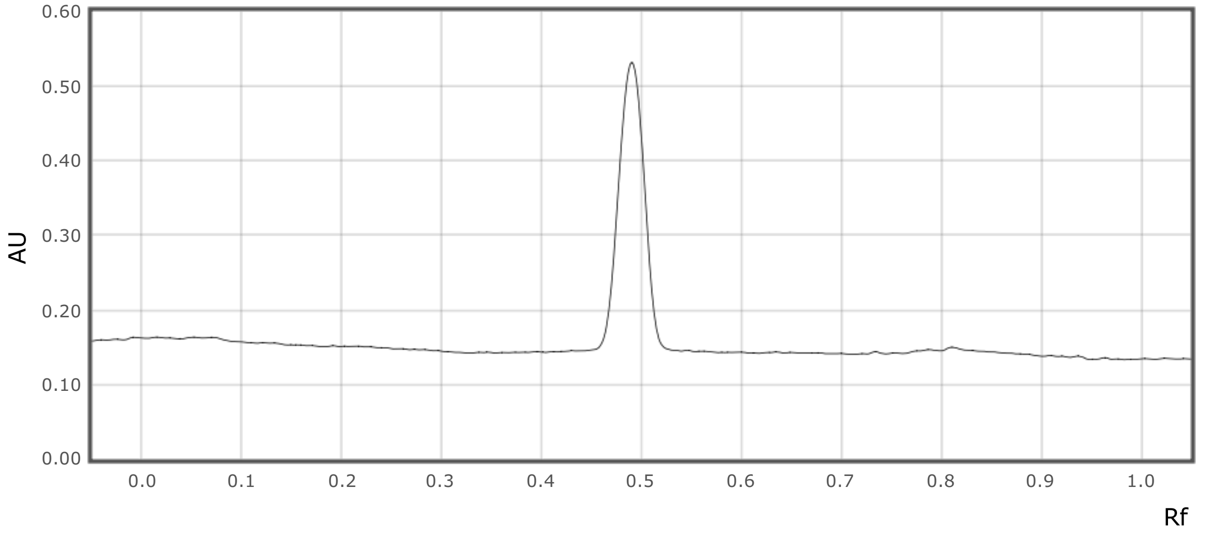

Track 8:

Type	Single λ

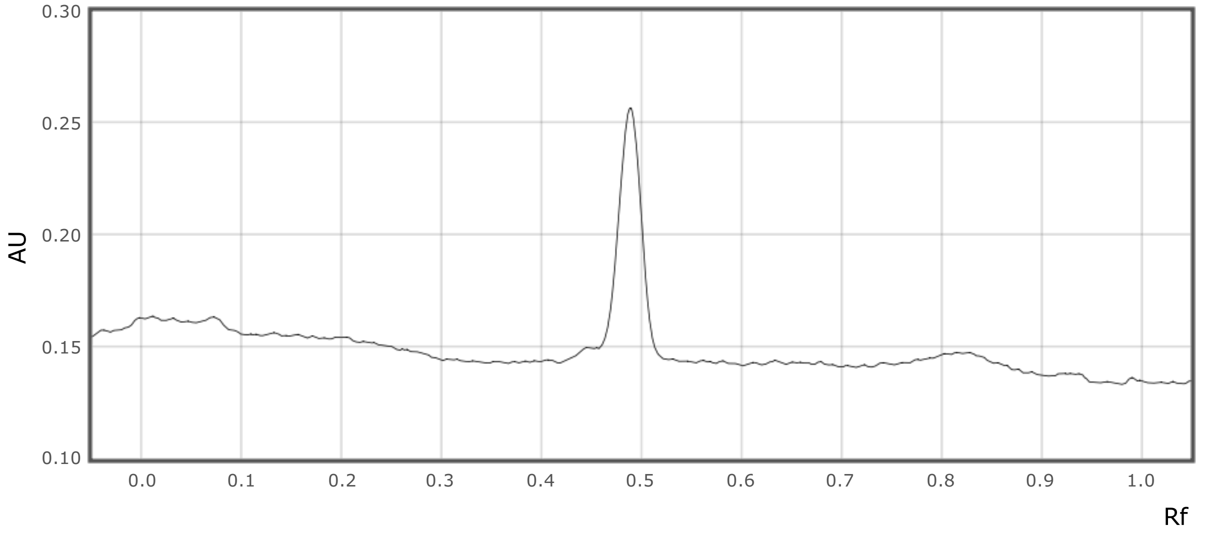

101, A Wing, Aniket Apartment,
Navghar Road, Mulund (E),
Mumbai - 400 081, India.
Tel.: 091 22 21639928 - 31
E-mail : hptlc@anchrom.in
Web : www.anchrom.in

Track 9:

Type	Single λ

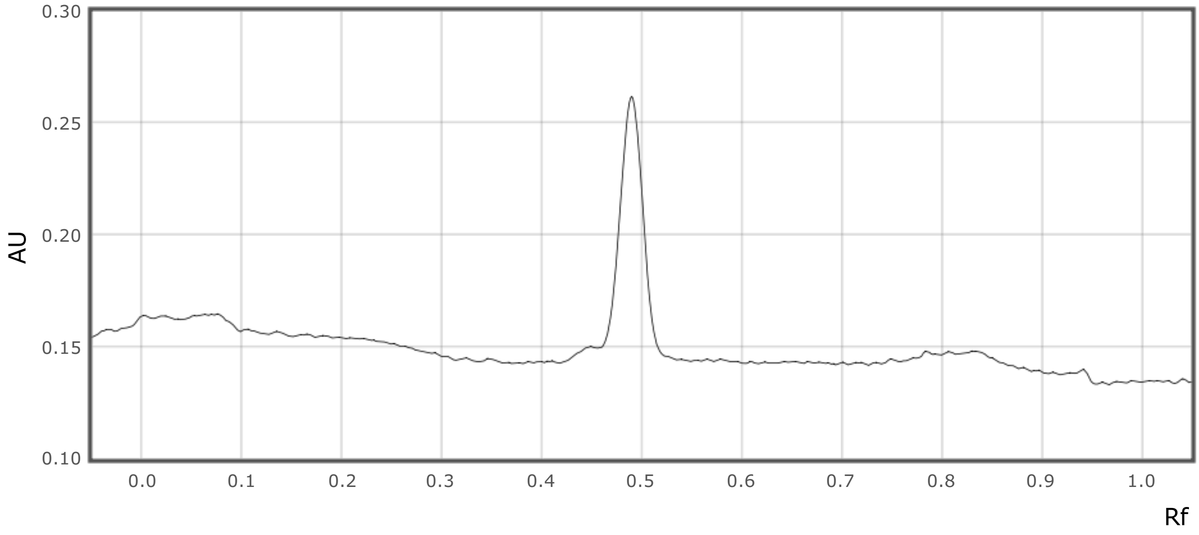

Track 10:

Type	Single λ

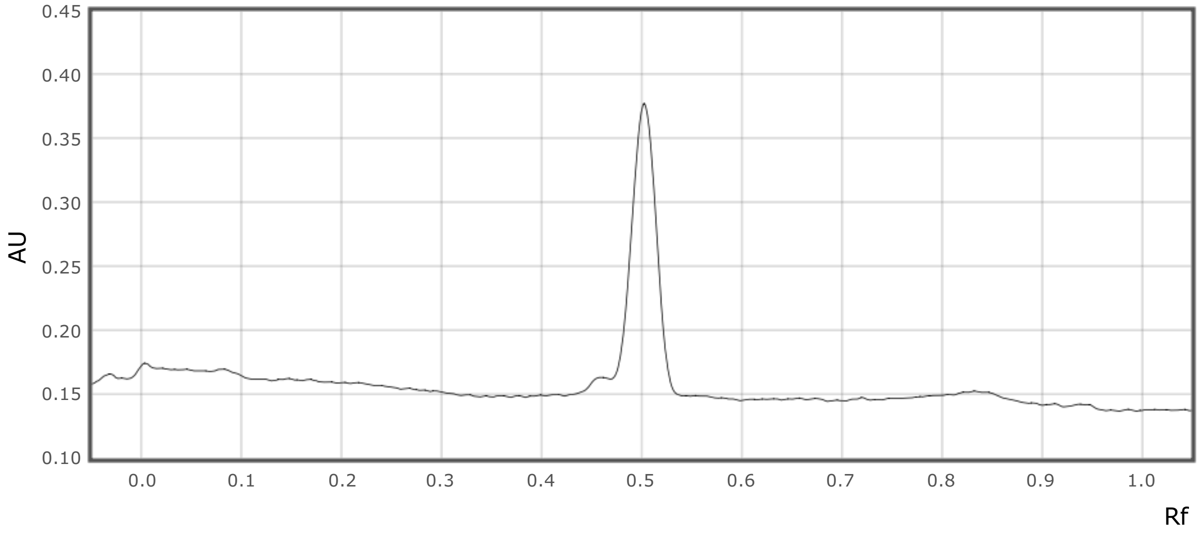

Track 11:

Type	Single λ

101, A Wing, Aniket Apartment,
Navghar Road, Mulund (E),
Mumbai - 400 081, India.
Tel.: 091 22 21639928 - 31
E-mail : hptlc@anchrom.in
Web : www.anchrom.in

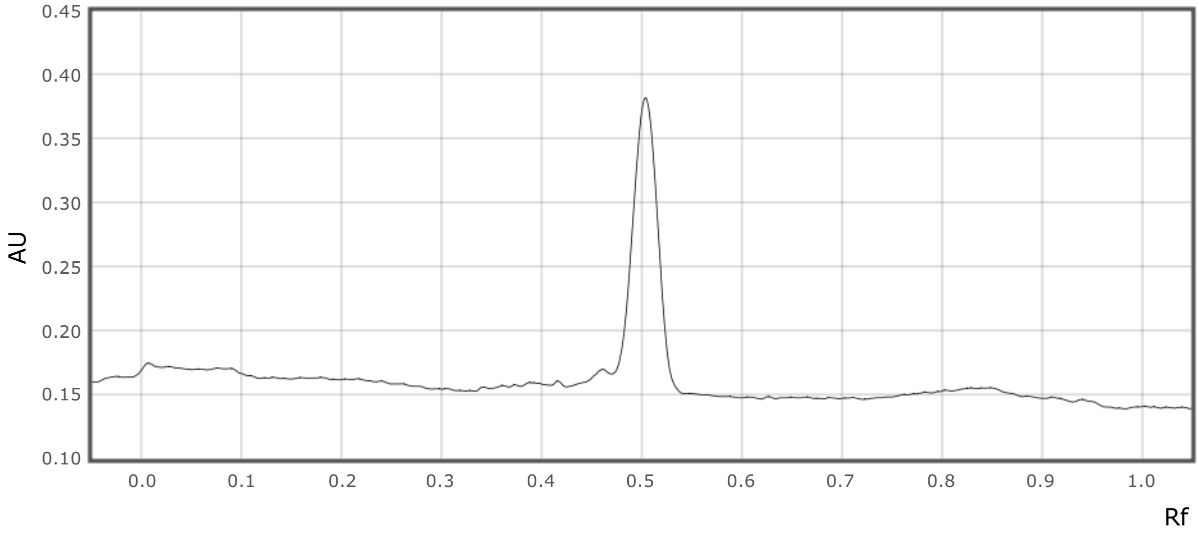

Track 12:

Type	Single λ

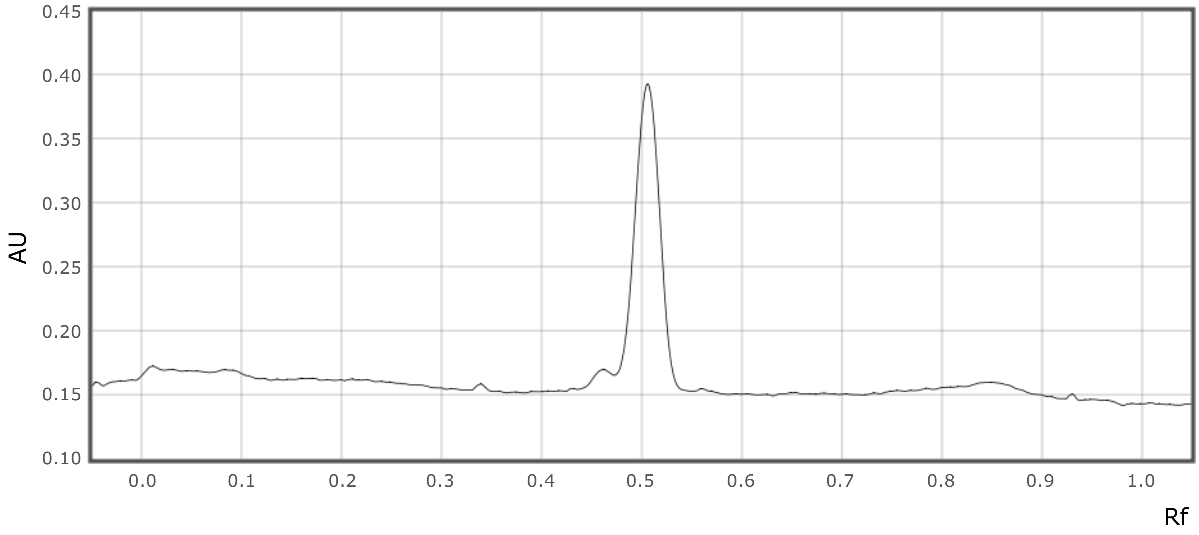

Spectrum Scan developed plate 1e - Scanner 4 (S/N: 170422):

Executed	04-Mar-2019 12:18:53 Lab_Sneha

Substance berberine HCl (Rf. 0.488 +/- 0.033):

Tr. 1 Rf 0.485 (20.0 mm, 38.1 mm)

101, A Wing, Aniket Apartment,
Navghar Road, Mulund (E),
Mumbai - 400 081, India.
Tel.: 091 22 21639928 - 31
E-mail : hptlc@anchrom.in
Web : www.anchrom.in

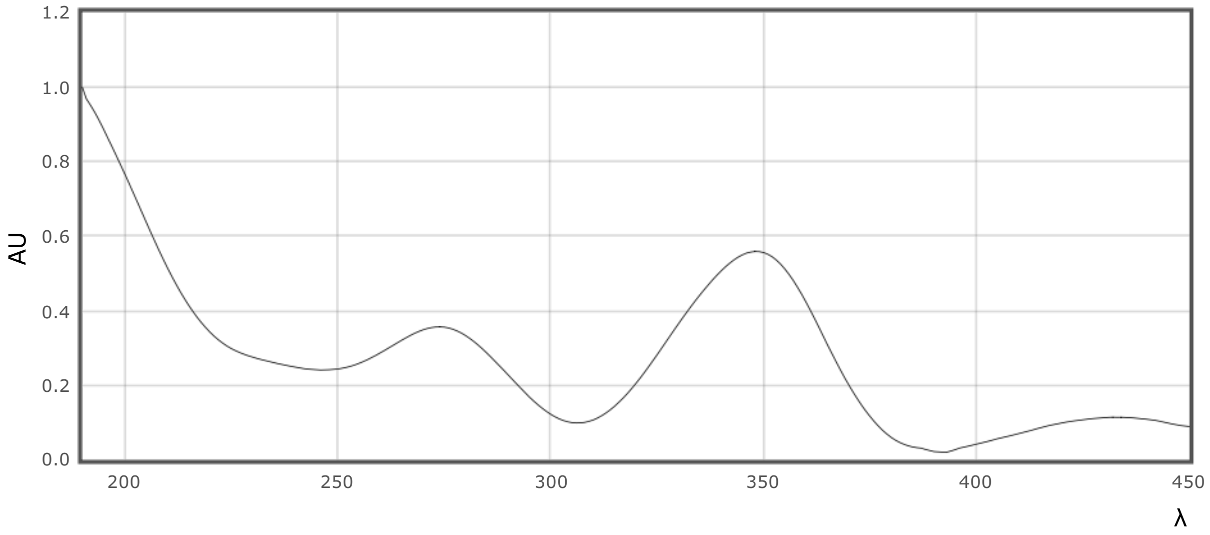

Tr. 2 Rf 0.481 (34.0 mm, 37.8 mm)

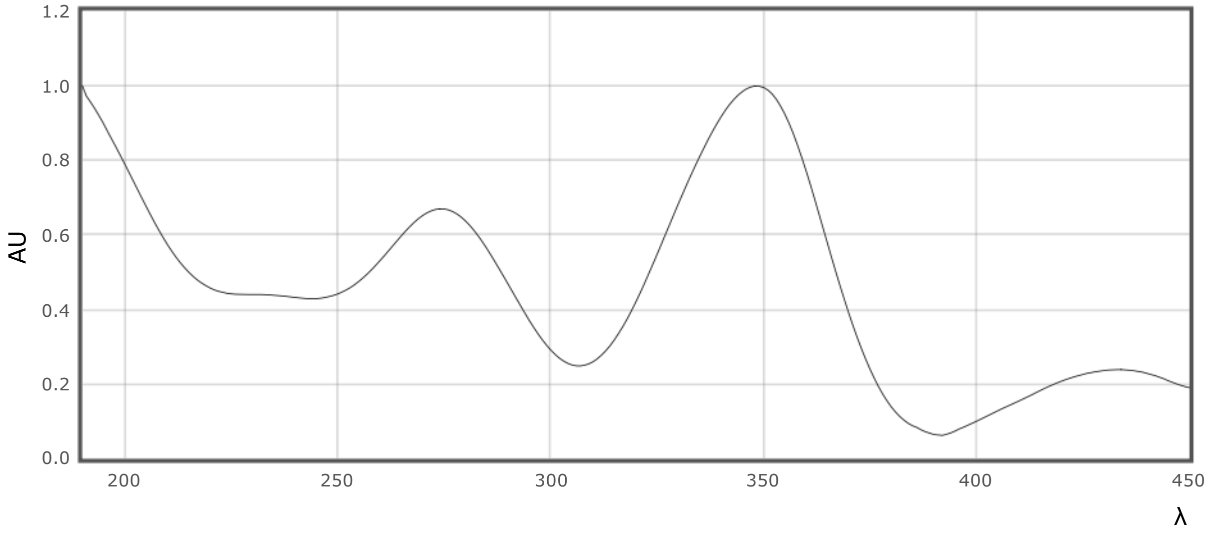

Tr. 3 Rf 0.482 (48.0 mm, 37.9 mm)

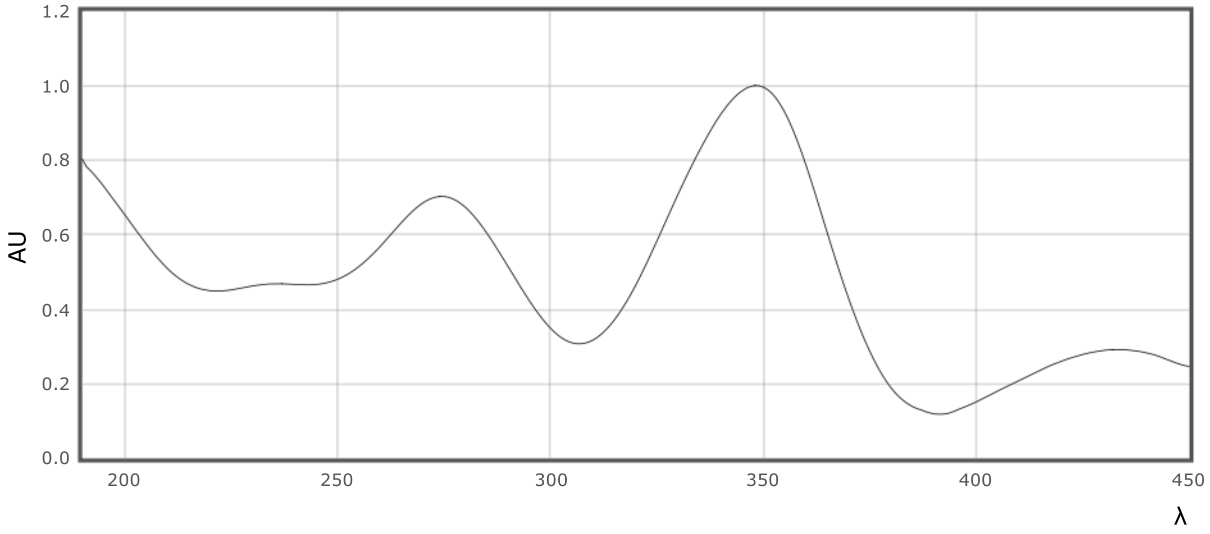

Tr. 4 Rf 0.481 (62.0 mm, 37.8 mm)

101, A Wing, Aniket Apartment,
Navghar Road, Mulund (E),
Mumbai - 400 081, India.
Tel.: 091 22 21639928 - 31
E-mail : hptlc@anchrom.in
Web : www.anchrom.in

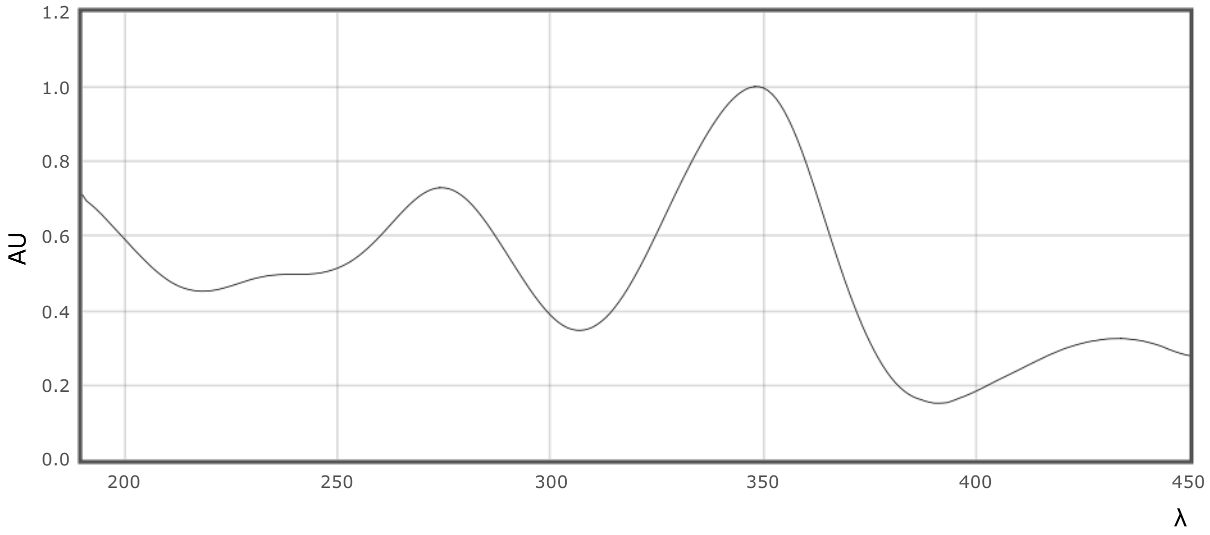

Tr. 5 Rf 0.481 (76.0 mm, 37.8 mm)

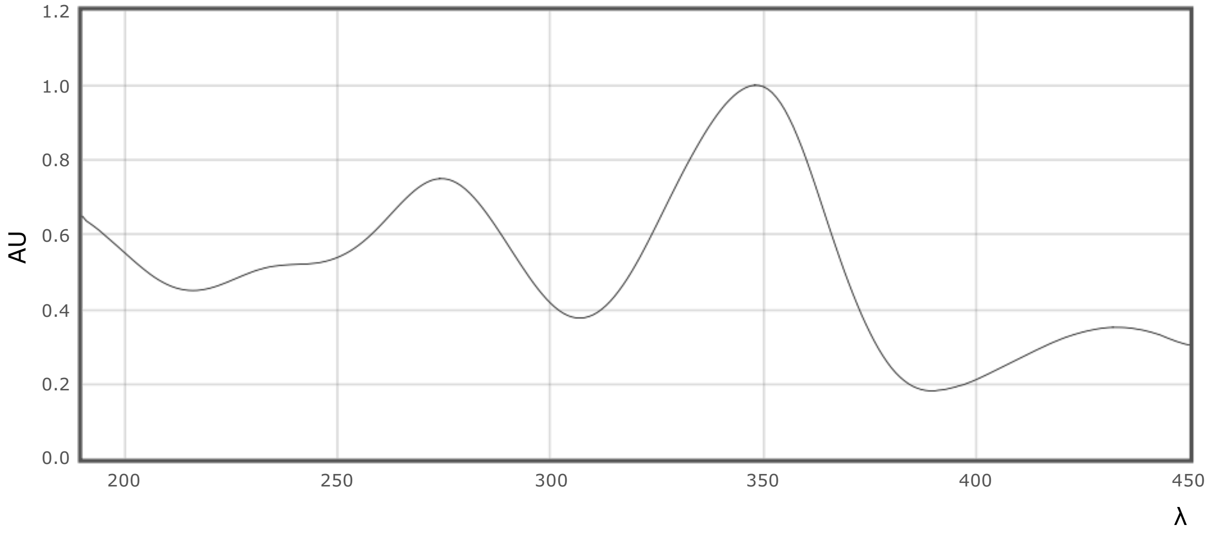

Tr. 6 Rf 0.489 (90.0 mm, 38.3 mm)

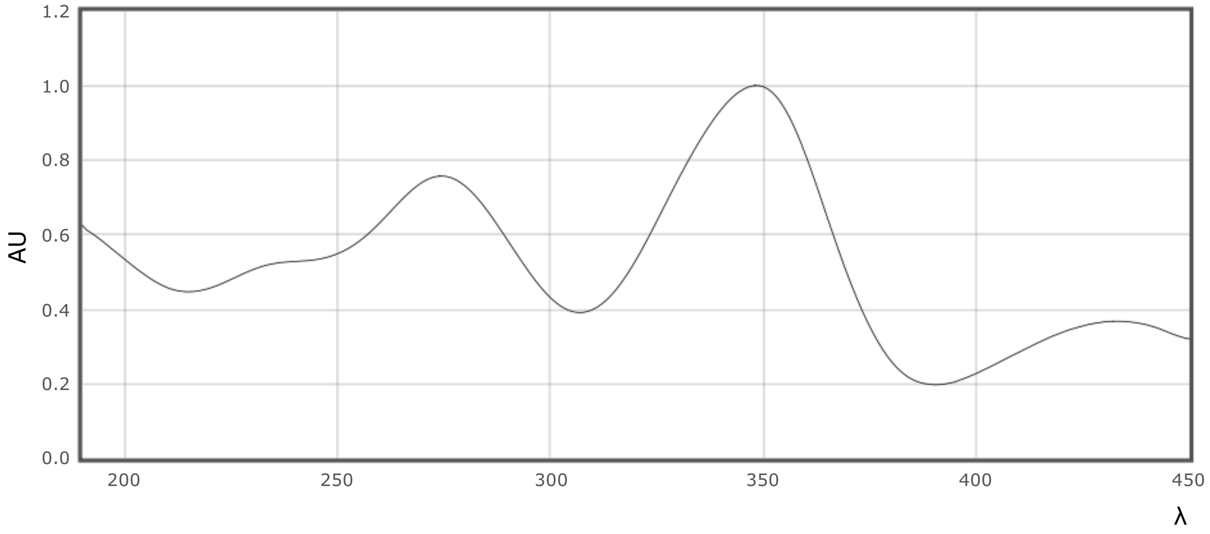

Tr. 7 Rf 0.492 (104.0 mm, 38.5 mm)

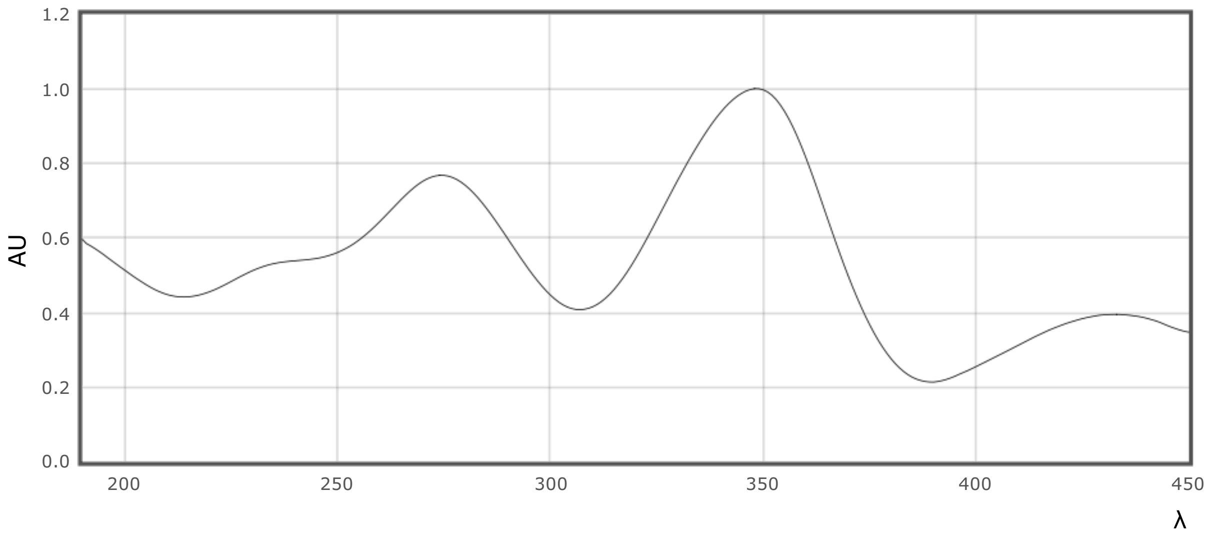

Tr. 8 Rf 0.489 (118.0 mm, 38.3 mm)

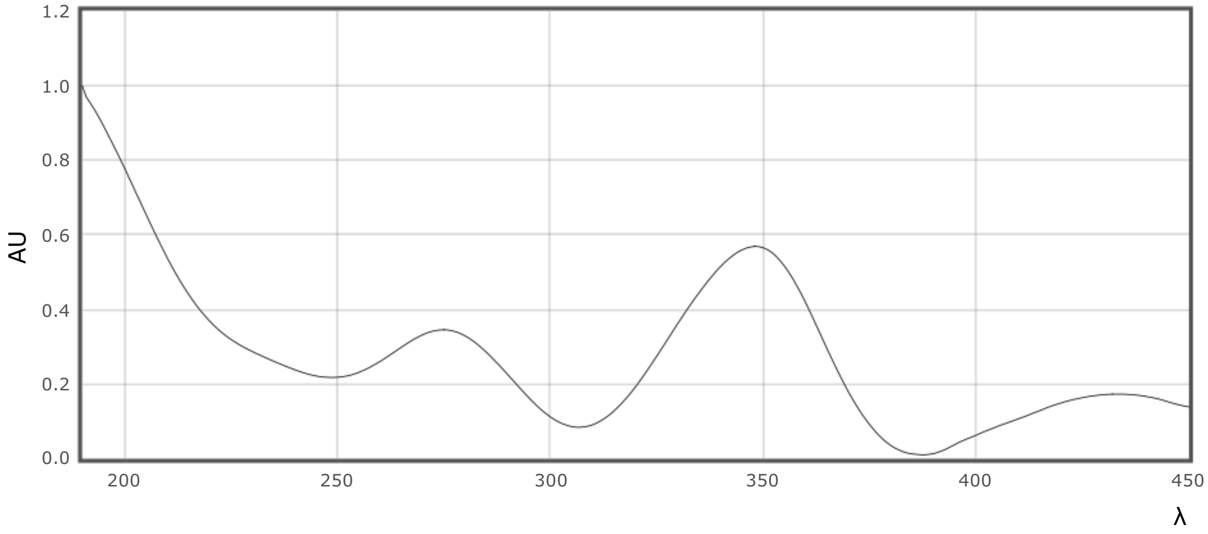

Tr. 9 Rf 0.490 (132.0 mm, 38.4 mm)

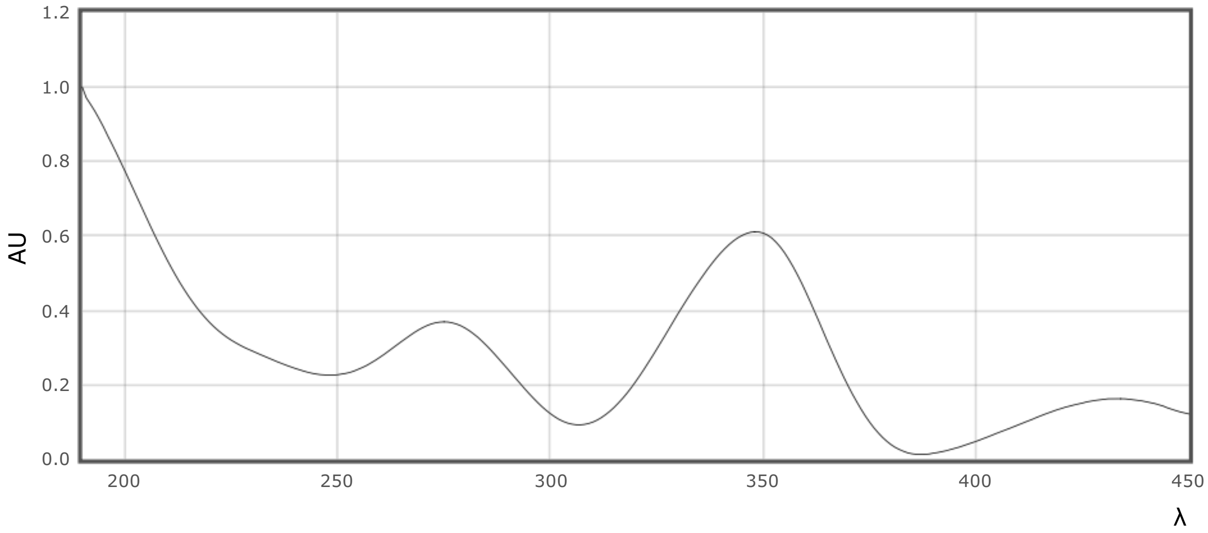

Tr. 10 Rf 0.502 (146.0 mm, 39.1 mm)

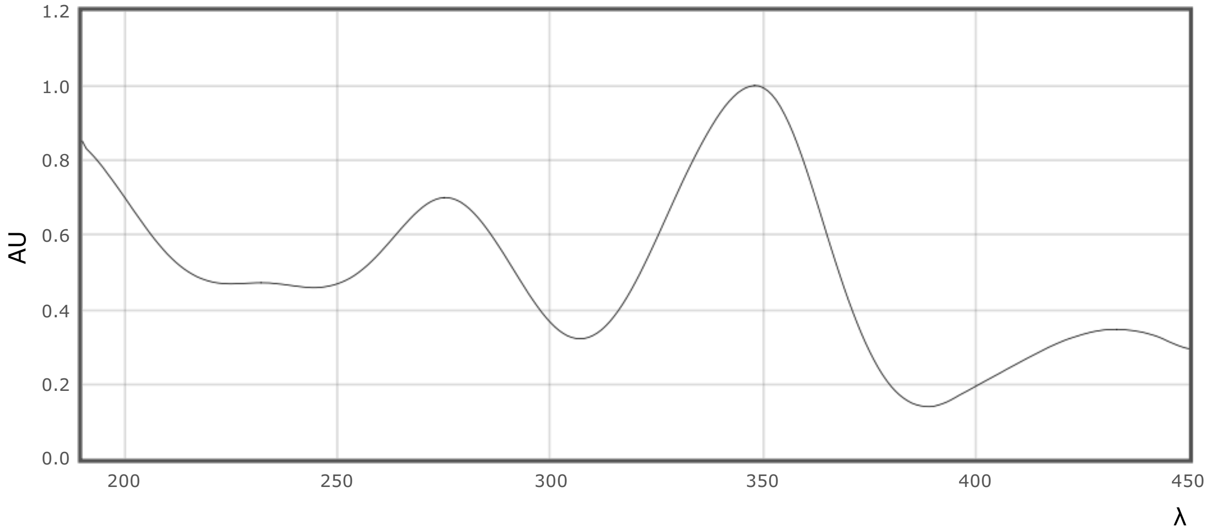

Tr. 11 Rf 0.505 (160.0 mm, 39.3 mm)

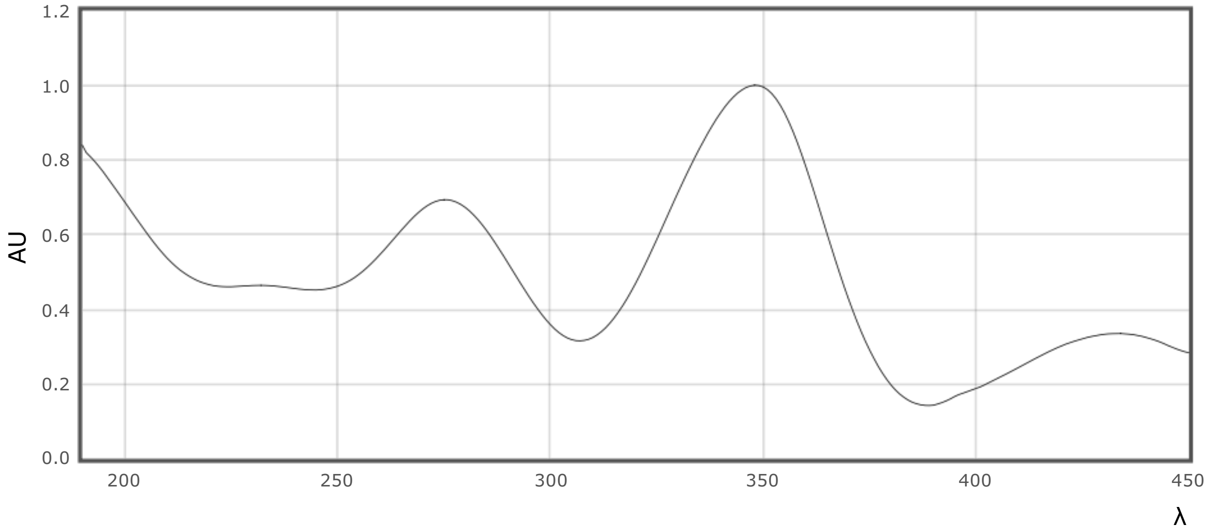

Tr. 12 Rf 0.506 (174.0 mm, 39.4 mm)

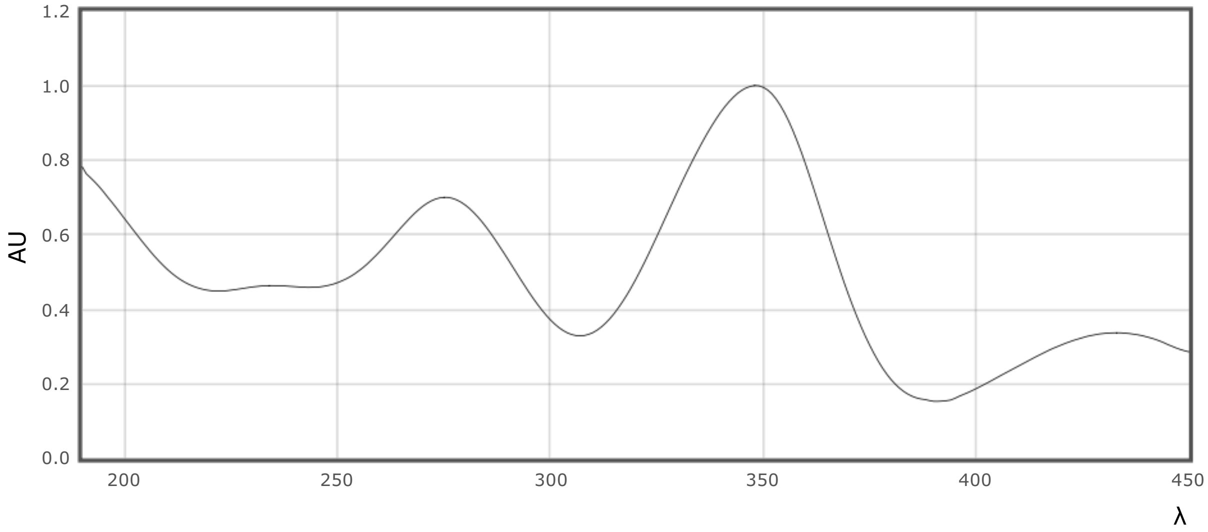

Spectrum correlation data:

101, A Wing, Aniket Apartment,
Navghar Road, Mulund (E),
Mumbai - 400 081, India.
Tel.: 091 22 21639928 - 31
E-mail : hptlc@anchrom.in
Web : www.anchrom.in

Substance name	Track	Rf	r(s,m)	r(e,m)	Ref. spectrum	Correlation
berberine HCl	1	0.485	0.000000	0.000000	Tr. 2, Rf 0.481, Sub. berberine HCl	0.903825
berberine HCl	2	0.481	0.000000	0.000000	Tr. 1, Rf 0.485, Sub. berberine HCl	0.903825
berberine HCl	3	0.482	0.000000	0.000000	Tr. 2, Rf 0.481, Sub. berberine HCl	0.984192
berberine HCl	4	0.481	0.000000	0.000000	Tr. 2, Rf 0.481, Sub. berberine HCl	0.959389
berberine HCl	5	0.481	0.000000	0.000000	Tr. 2, Rf 0.481, Sub. berberine HCl	0.933514
berberine HCl	6	0.489	0.000000	0.000000	Tr. 2, Rf 0.481, Sub. berberine HCl	0.918923
berberine HCl	7	0.492	0.000000	0.000000	Tr. 2, Rf 0.481, Sub. berberine HCl	0.897320
berberine HCl	8	0.489	0.000000	0.000000	Tr. 2, Rf 0.481, Sub. berberine HCl	0.881638
berberine HCl	9	0.490	0.000000	0.000000	Tr. 2, Rf 0.481, Sub. berberine HCl	0.911528
berberine HCl	10	0.502	0.000000	0.000000	Tr. 2, Rf 0.481, Sub. berberine HCl	0.989198
berberine HCl	11	0.505	0.000000	0.000000	Tr. 2, Rf 0.481, Sub. berberine HCl	0.988581
berberine HCl	12	0.506	0.000000	0.000000	Tr. 2, Rf 0.481, Sub. berberine HCl	0.978085

Evaluation 1 :

Validated	true
Step	Scan developed plate 1d
Concentration unit type	Mass / volume
Notes	

Definition:

References:

RA1819284-01		
Substance Name	Concentration	Purity
berberine chloride	100.000 µg/ml	100.00 %

Samples:

Vial ID	Amount	Volume solution	Reference amount	Related to
SA1819284-01	1.000 mg	1.00 ml	0.000 mg	

Integration parameters:

Bounds	[0.000,1.000]
Smoothing	Savitzky–Golay of order 3 and window 7
Baseline correction	Lowest slope with noise 0.05
Profile subtraction	None
Peaks detection	Gauss (legacy) with sensitivity 0.1, separation 1 and threshold 0.1

Scan:

Wavelength	366 nm

Track 2:

Type	Reference
Vial ID	RA1819284-01
Description	Berberine Chloride(0.1 mg/ml)
Volume	2.0 µl

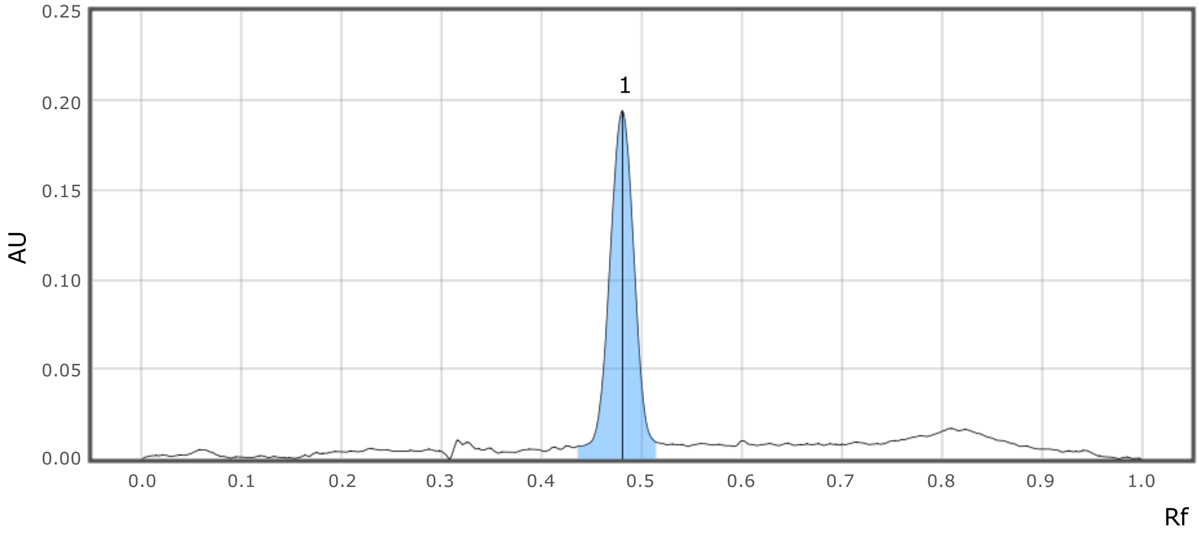

Peak #	Start		Max			End		Area		Manual peak	Substance Name
	Rf	H	Rf	H	%	Rf	H	A	%		
1	0.434	0.0066	0.481	0.1942	100.00	0.515	0.0090	0.00567	100.00	No	berberine chloride

Track 3:

Type	Reference
Vial ID	RA1819284-01
Description	Berberine Chloride(0.1 mg/ml)
Volume	3.0 µl

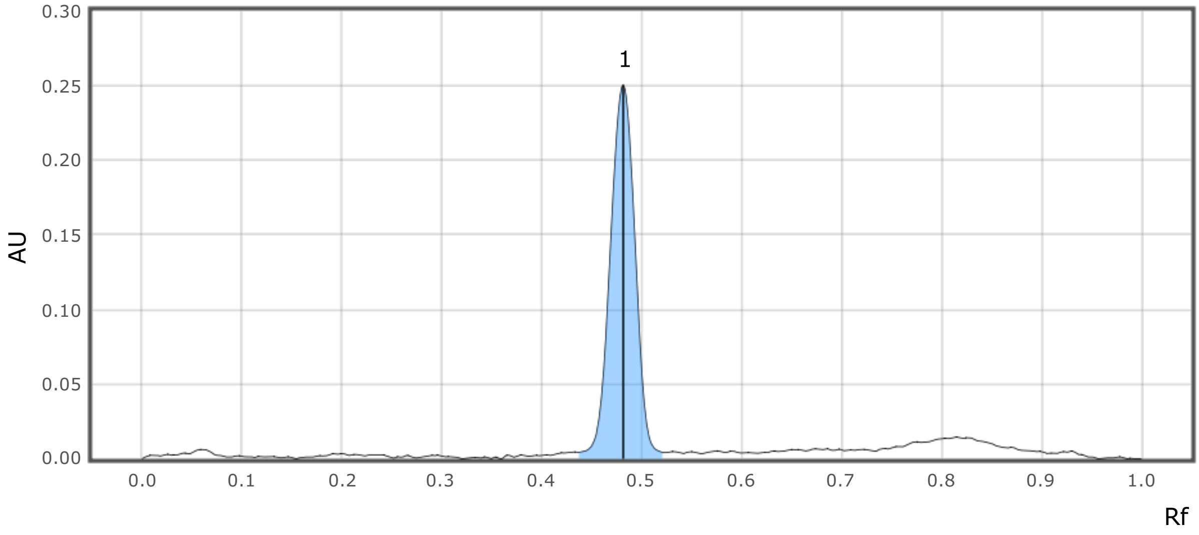

Peak #	Start		Max			End		Area		Manual peak	Substance Name
	Rf	H	Rf	H	%	Rf	H	A	%		
1	0.437	0.0042	0.481	0.2501	100.00	0.521	0.0041	0.00723	100.00	No	berberine chloride

Track 4:

Type	Reference
Vial ID	RA1819284-01
Description	Berberine Chloride(0.1 mg/ml)
Volume	4.0 µl

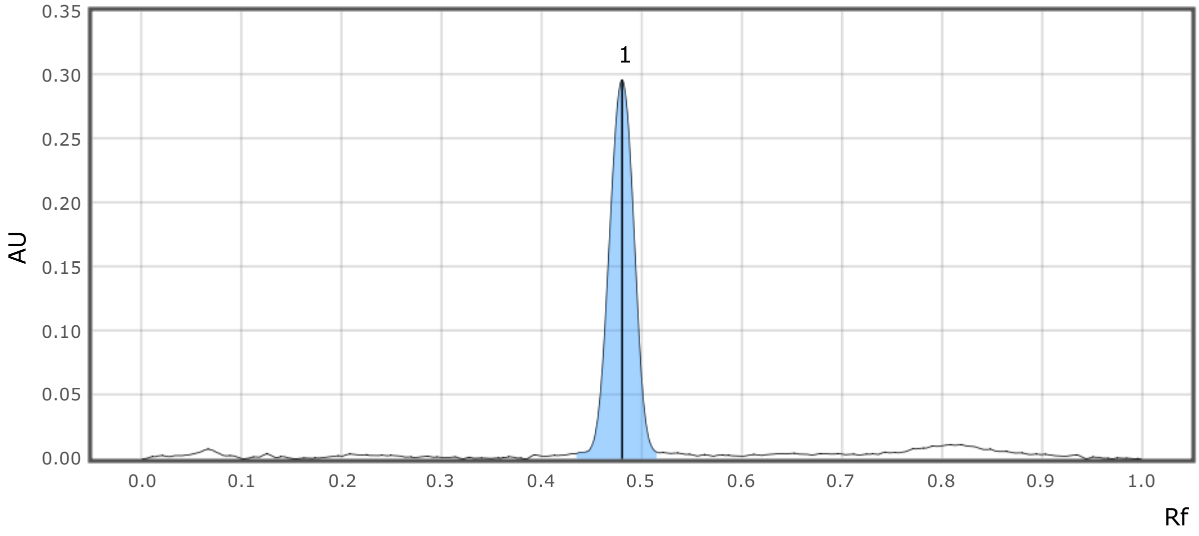

Peak #	Start		Max			End		Area		Manual peak	Substance Name
	Rf	H	Rf	H	%	Rf	H	A	%		
1	0.433	0.0041	0.480	0.2960	100.00	0.517	0.0047	0.00868	100.00	No	berberine chloride

Track 5:	
Type	Reference
Vial ID	RA1819284-01
Description	Berberine Chloride(0.1 mg/ml)
Volume	5.0 µl

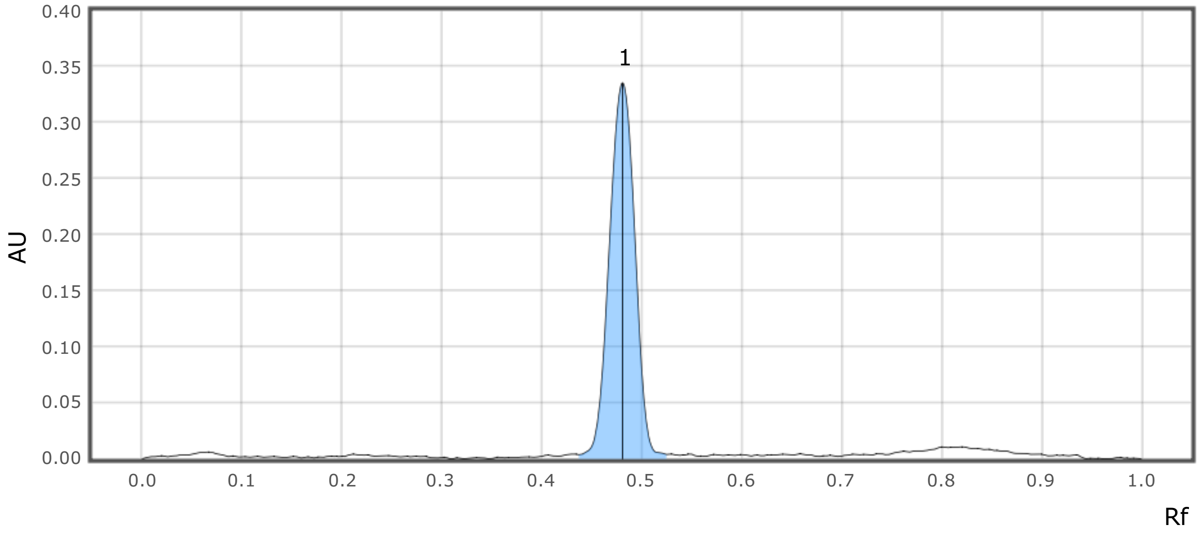

Peak #	Start		Max			End		Area		Manual peak	Substance Name
	Rf	H	Rf	H	%	Rf	H	A	%		
1	0.437	0.0036	0.481	0.3354	100.00	0.525	0.0038	0.00996	100.00	No	berberine chloride

Track 6:	
Type	Reference
Vial ID	RA1819284-01
Description	Berberine Chloride(0.1 mg/ml)
Volume	6.0 µl

101, A Wing, Aniket Apartment,
Navghar Road, Mulund (E),
Mumbai - 400 081, India.
Tel.: 091 22 21639928 - 31
E-mail : hptlc@anchrom.in
Web : www.anchrom.in

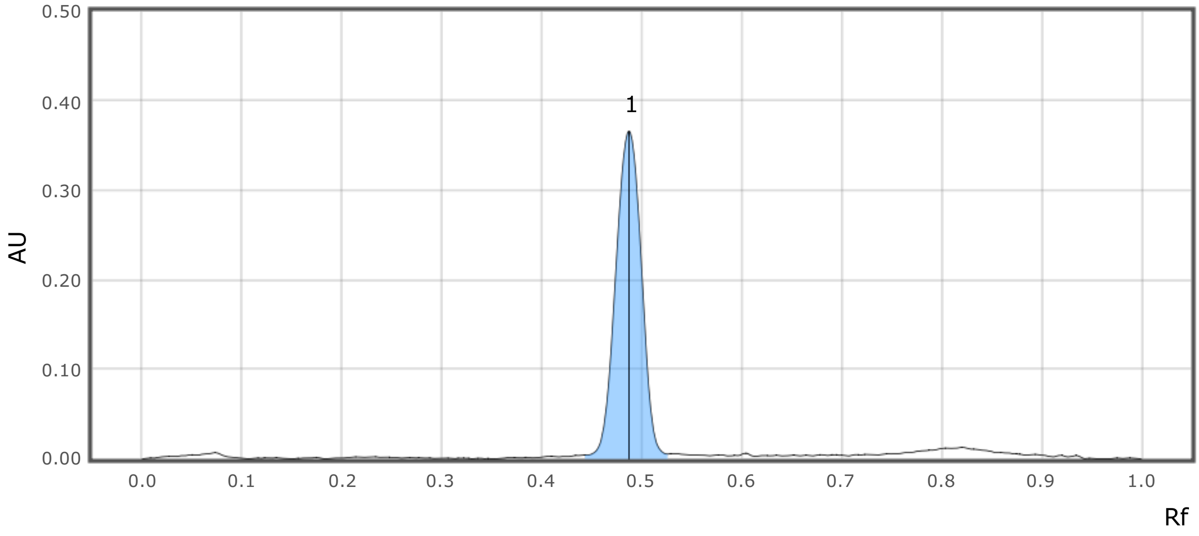

Peak #	Start		Max			End		Area		Manual peak	Substance Name
	Rf	H	Rf	H	%	Rf	H	A	%		
1	0.438	0.0037	0.487	0.3659	100.00	0.526	0.0053	0.01096	100.00	No	berberine chloride

Track 10:	
Type	Sample
Vial ID	SA1819284-01
Description	Berberis aristata(10 mg/ml)_1:100
Volume	5.0 µl

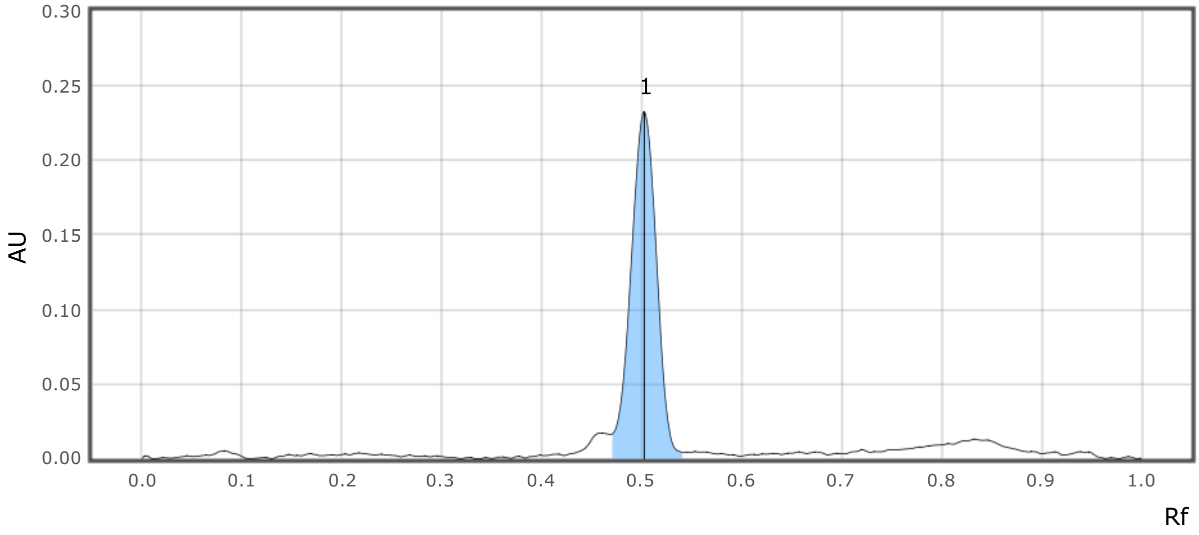

Peak #	Start		Max			End		Area		Manual peak	Substance Name
	Rf	H	Rf	H	%	Rf	H	A	%		
1	0.469	0.0162	0.502	0.2323	100.00	0.541	0.0042	0.00690	100.00	No	berberine chloride

Track 11:	
Type	Sample
Vial ID	SA1819284-01
Description	Berberis aristata(10 mg/ml)_1:100
Volume	5.0 µl

101, A Wing, Aniket Apartment,
Navghar Road, Mulund (E),
Mumbai - 400 081, India.
Tel.: 091 22 21639928 - 31
E-mail : hptlc@anchrom.in
Web : www.anchrom.in

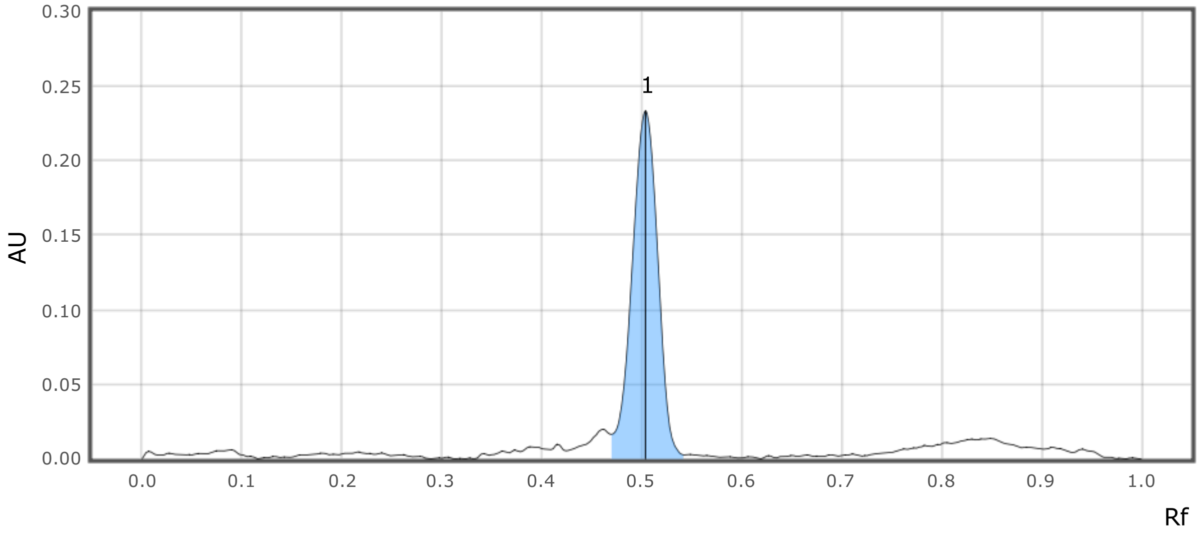

Peak #	Start		Max			End		Area		Manual peak	Substance Name
	Rf	H	Rf	H	%	Rf	H	A	%		
1	0.470	0.0164	0.504	0.2330	100.00	0.542	0.0026	0.00695	100.00	No	berberine chloride

Calibration results:

Height calibration for substance berberine chloride @ 366 nm:

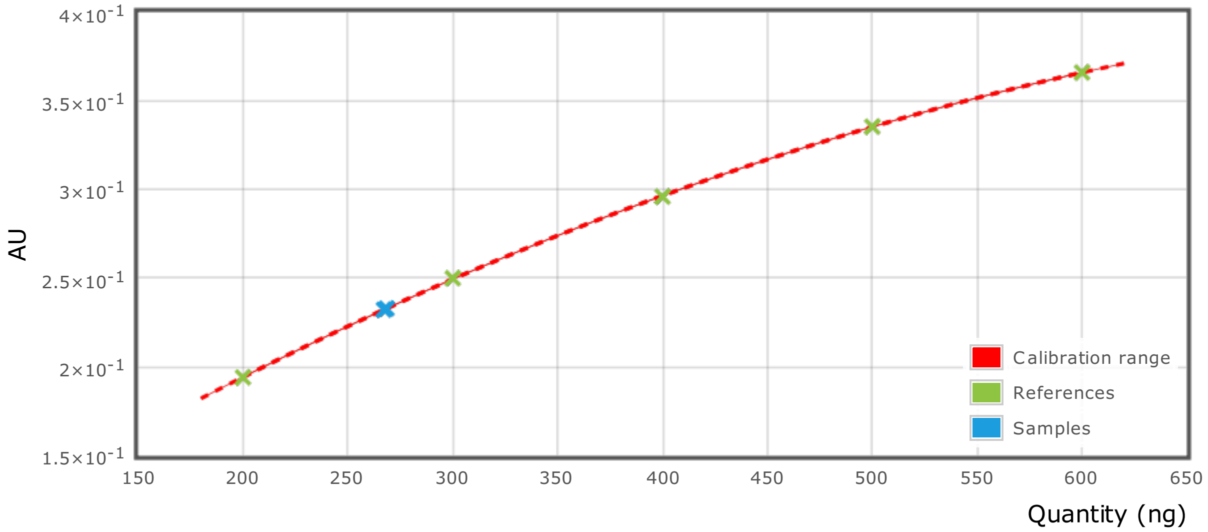

Regression mode	Polynomial
Range deviation	5.00 %
Related substances	Default
Number of references	5
Calibration function	$y=-4.097\times10^{-13}x^2+7.566\times10^{-7}x+5.943\times10^{-2}$
Coefficient of variation	CV 0.12 %
Correlation coefficient	R=99.998448 %

Results:

101, A Wing, Aniket Apartment,
Navghar Road, Mulund (E),
Mumbai - 400 081, India.
Tel.: 091 22 21639928 - 31
E-mail : hptlc@anchrom.in
Web : www.anchrom.in

berberine chloride	(2 sample assignments) @ 366 nm		
Sample 'SA1819284-01'	53.56 µg/ml	CV=0.34 %	(2 applications)
			53.56 µg in 1.000 mg
Volume: 5.0 µl	53.56 µg/ml	CV=0.34 %	(2 replicas)
Track 10	53.43 µg/ml	267.2 ng	
Track 11	53.69 µg/ml	268.4 ng	

A track marked with ⚠ means: this result is outside the regression range given by the reference assignments, but is included in the results because it is in the allowed range deviation.

Remarks

Conclusion: The Berberine chloride content in following sample is 53.5µg in 1 mg of solution which is approximately 5.4%.

E-signed

Level: 1 - Lab Analyst

Signed by	Lab_Sneha
Date	05-Mar-2019 11:34:51 UTC
Comment	final result

Level: 2 - Asst. Lab Manager

Signed by	Lab_saikat
Date	05-Mar-2019 11:35:07 UTC
Comment	OK
